# Azadistibiridines and stabilised-iminobismuthane: reactivity of small inorganic rings in heavy main group chemistry

**DOI:** 10.1039/d5sc03416g

**Published:** 2025-08-19

**Authors:** Prasenjit Palui, Matthias Bollenbeck, Daniel Meleschko, Philipp Brehm, Rosa M. Gomila, Gregor Schnakenburg, Antonio Frontera, Alessandro Bismuto

**Affiliations:** a Institute of Inorganic Chemistry, University of Bonn Gerhard-Domagk-Str. 1 53121 Bonn Germany bismuto@uni-bonn.de; b Departament de Química, Universitat de les Illes Balears Crta. de Valldemossa km 7.5 07122 Palma Baleares Spain

## Abstract

Small organic rings are important molecular entities that have long played a crucial role in organic synthesis and medicinal chemistry. Similarly, small inorganic rings have shown promise in promoting bond activation and, in some cases, catalysis, although their synthesis is significantly more challenging. Here, we present a detailed experimental and theoretical investigation of three-membered heterocycles, azadistibiridines (2a–2d), synthesised *via* cycloaddition of a distibene (Sb_2_Tbb_2_) with various azides. Additionally, for the first time, we were able to extend this protocol to dibismuthene (Bi_2_Tbb_2_), usually a more inert fragment, generating an iminobismuthane dimer (4a), showing divergent reactivity compared to that of distibene. Taking advantage of the strain imposed by these rings, we have been able to explore the reactivity of both classes of compounds, leading to the isolation of a ring-expanded product with GeBr_2_ (5) and a rare example of NHC-supported iminobismuthane (6). These findings represent a significant theoretical and practical advancement in main group chemistry where small inorganic rings have been used as tools to enhance the reactivity of heavy pnictogen multiple bonds.

## Abstract 2

This work showed the activation of heavy dipnictenes with azides to generate small inorganic rings. Their synthetic potential has been thoroughly analysed and leveraged to obtain a ring insertion reaction and access a stabilised imino-bismuthane.

## Graphical abstract

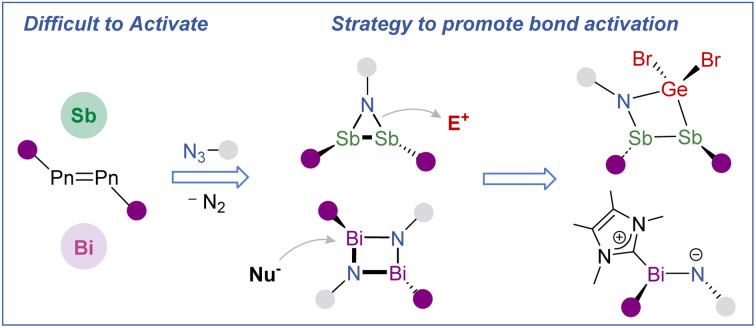

## Introduction

**Fig. 1:**
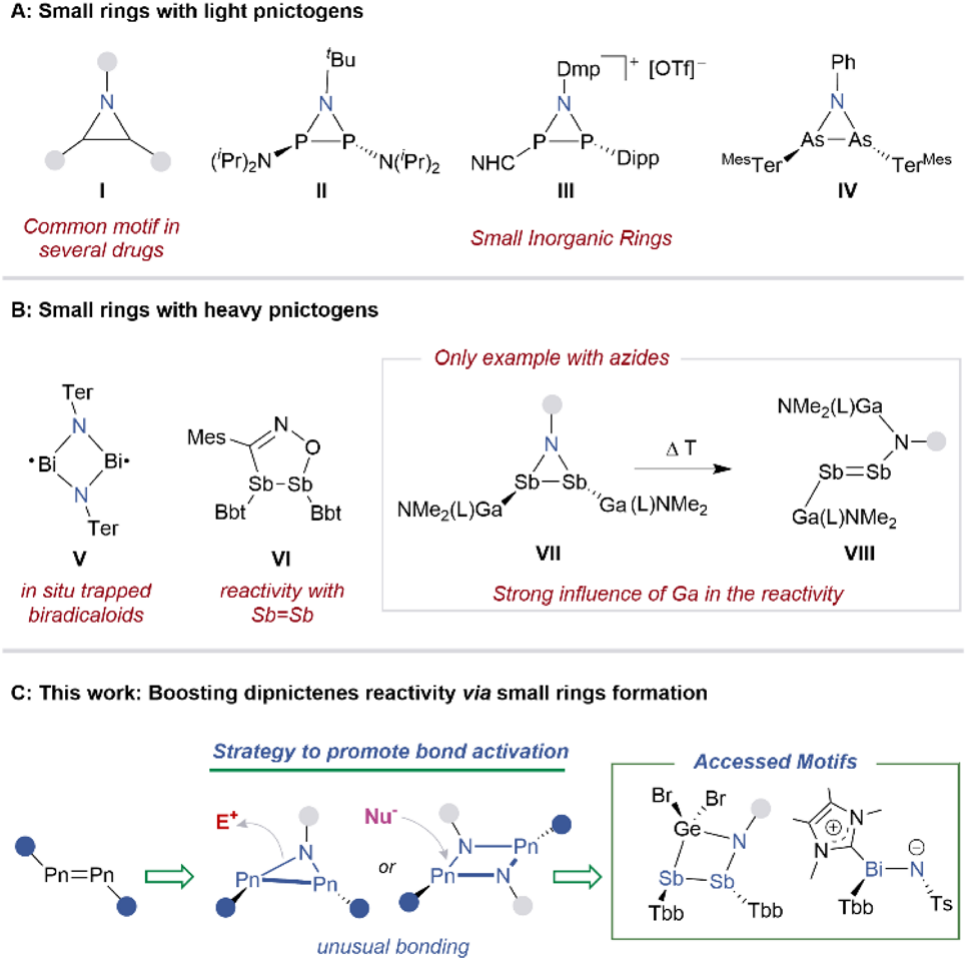
(A) Small rings with light pnictogens. (B) Small rings with heavy pnictogens. (C) This work: synthesis and reactivity of heavy azadipnictiridines; NHC = 4,5-dichloro 1,3-bis(Dipp)-imidazol-2-yl; Dipp = 2,6-diisopropylphenyl; Dmp = 2,5-dimethylphenyl; Ter = Ter^Mes^ = 2,6-bis(2,4,6-trimethylphenyl)-phenyl; Bbt = 2,6-bis[bis(trimethylsilyl)methyl]-4-[tris(trimethylsilyl)methyl]phenyl; Tbb = 2,6-[CH(SiMe_3_)_2_]_2_-4-^*t*^Bu-C_6_H_2_.

The electronic features and the reactivity of small rings have fascinated chemists for a long time.^1^ The geometry and the rigidity enforced by three- and four-membered rings lead to a completely new bonding scenario where the bond angles and the hybridisation stand out from the usual expected.^2–5^ Subsequently, such motifs readily release ring-strain through cycloaddition reactions or by reacting with nucleophiles or electrophiles, allowing for facile diversification.^6,7^ In particular, aziridines (I, Fig. 1A) serve as common building blocks in a variety of drugs as they can strongly influence the lipophilicity, hydrogen bonding, and metabolic stability.^8^

The curiosity behind molecular editing has driven the search for synthetic pathways to replace carbon atoms in strained rings with other main group elements, leading to the development of inorganic versions of these systems.^2^ This relatively small change allows for new electronic features and reactivity patterns that are not accessible in purely organic frameworks.^9,10^ Niecke was one of the pioneers in this field, replacing carbon with phosphorus and reporting the first example of an azadiphosphiridine by base induced coupling of an iminophosphorane and a phosphine (II, Fig. 1A).^11^ Since then, several other strategies have been used to synthesise similar moieties with heavier elements. The two most successful synthetic pathways are (1) metathesis reactions with alkali metal amides^12,13^ and (2) cycloaddition reactions of azides to the heavy pnictogen double bonds.^14,15^ Both present some drawbacks such as the difficult tunability of the amide substituents^13^ and the limited reactivity of heavy pnictogen double bonds.^16^ These two factors resulted in only few heavy azadipnictiridine rings available to-date. However, employing the salt metathesis approach, several groups have accessed a number of four-membered diazadipnictetidines starting from antimony or bismuth halide precursors.^12,13,17,18^ Thereafter, Axel Schulz and co-workers successfully reported a new domain for four-membered pnictogen rings with biradicaloid electronic structures, and explored their efficiency in small molecule activations (V, Fig. 1B).^18–20^ Thanks to an elaborate ligand design, researchers developed cycloaddition protocols as an alternative method to synthesise small inorganic rings.^21,22^ Weigand and co-workers utilised an N-heterocyclic carbene (NHC)-supported cationic diphosphene in cycloaddition reactions with azides, leading to the formation of azadiphosphiridine salts (III, Fig. 1A).^15^ With a similar strategy, Hering-Junghans, Werncke and co-workers reported the synthesis of an azadiarsacyclopropane upon reacting a diarsene radical anion with phenyl azide (IV, Fig. 1A).^23^

Unfortunately, descending the pnictogen group, the feasibility of dipolar cycloaddition reactions with dipnictenes decreases significantly with only two examples reported for antimony but none for bismuth.^24–26^ The first case was reported by Tokitoh, Sasamori, and co-workers who performed a formal [3 + 2]-cycloaddition between distibene and a nitrile oxide, resulting in a five-membered Sb_2_-heterocycle (VI, Fig. 1B).^24^ After several years, Stefan Schulz and co-workers synthesised a new gallyl substituted distibene and investigated the reaction with different azides to obtain azadistibiridines (VII, Fig. 1B).^26^ Unfortunately, the presence of the gallium centre hampered any further functionalisation due to a rearrangement occurring *via* an insertion of the amino group into the Ga–Sb bond (VIII, Fig. 1B). Additionally, despite the unique ligand design, it was not possible to promote activation of the dibismuthene double bond, highlighting the huge challenge implicit with such a motif.

Since ring strain has been successfully utilised in phosphorus chemistry to promote bond activation^9^ and even in catalysis,^27,28^ a natural development would be to extend it to antimony and bismuth chemistry. Thus, we envisaged that small heavy pnictogen rings (Pn = Sb, Bi) with a more inert ligand would allow for further functionalisation, unleashing the full potential of these unique structures (Fig. 1C). We thus wondered whether 2,6-[CH(SiMe_3_)_2_]_2_-4-^*t*^Bu-C_6_H_2_ (Tbb) would be the right compromise to selectively obtain azadipnictiridines with limited side reactivity. This would create a strong practical and theoretical advantage, providing a simple way to access heavy pnictogen rings and allowing us to exploit the unique reactivity arising from these strained structures.

## Results and discussion

### Design and characterisation of azadistibiridines

**Scheme 1:**
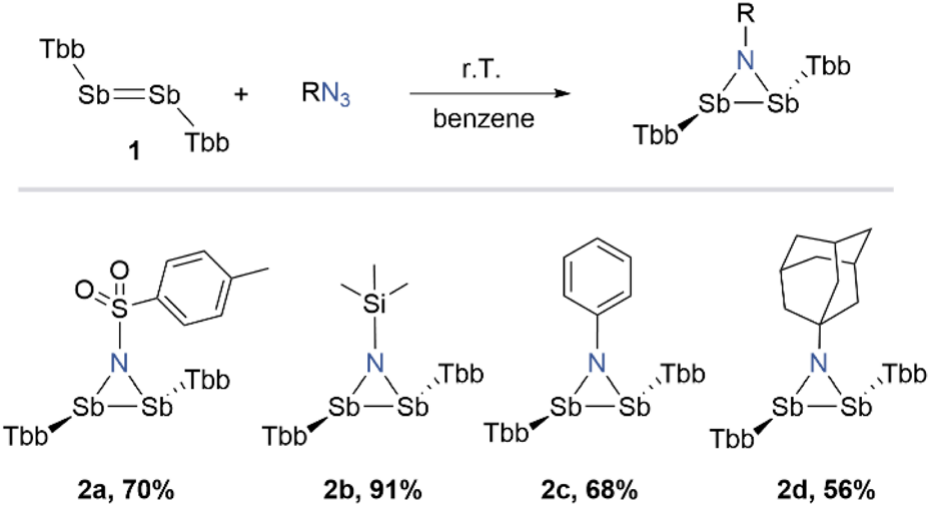
Synthesis of azadistibiridines (2a–2d).

We started our investigation by reacting Sb_2_Tbb_2_ (1) with tosyl azide (Ts-N_3_) as a benchmark substrate. Upon treatment of the yellowish-orange suspension of Sb_2_Tbb_2_ in benzene with a stock solution of tosyl azide at room temperature, an immediate gas evolution was observed with the suspension gradually turning to a clear yellow solution. ^1^H NMR spectroscopic analysis of the reaction aliquot confirmed the complete consumption of the starting materials within 2 hours and a very selective formation of the three-membered ring 2a (Scheme 1). After work-up and crystallisation from *n*-pentane at −30 °C, we could isolate product 2a as an air sensitive, yellow crystalline solid in 70% yield. Following the reaction outcome with tosyl azide, we wondered whether this approach could be extended to other classes of azides, leading to electronically diverse systems. When Sb_2_Tbb_2_ was allowed to react with trimethyl silyl-, phenyl-, and adamantyl-azide, a similar spontaneous reaction occurred with selective formation of the corresponding azadistibiridines (2b–2d) (Scheme 1). All compounds were isolated in ananalytically pure form as air-sensitive crystalline solids (2b yellow, 2c red, and 2d orange) in moderate to good yields.

Interestingly, none of the azadistibiridines reacted further with an additional equivalent of the azide to undergo bis-azidirination of the dipnictogen double bond. NMR spectroscopic studies revealed no other detectable intermediates than the final products. All isolated compounds were stable at 80 °C in benzene without any appreciable decomposition. This provides further support to our main hypothesis that a modified phenyl-based ligand system would lead to rings with a higher stability, thus allowing us to uncover their potential reactivity. Unfortunately, attempts to activate sterically hindered azide (*e.g.* Ar^Mes^N_3_, Ar^Mes^ = C_6_H_3_-2,6-Mes_2_, Mes = 2,4,6-trimethylphenyl) to make azadistibiridines were not successful, even after changing the ligand systems on the pnictogen centre to Bbt or Ar^Mes^.

**Fig. 2:**
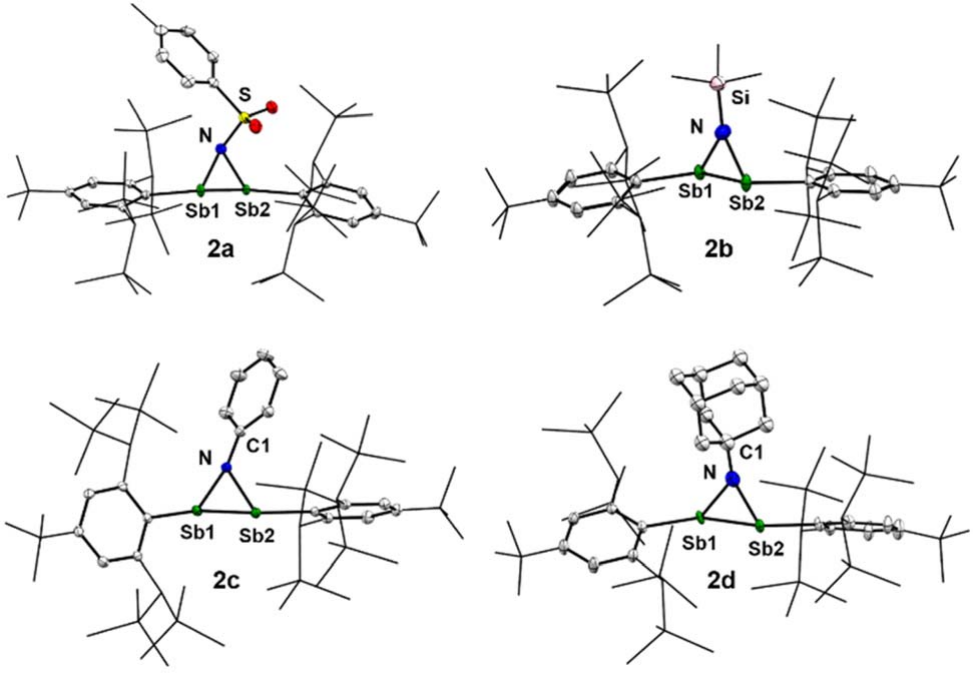
Single-crystal X-ray structures of azadistibiridines (2a–2d). Displacement ellipsoids are drawn at 30% probability level. Hydrogen atoms are omitted for clarity.

Compounds (2a–2d) were characterised by elemental analysis, NMR and UV/vis spectroscopies, and single-crystal X-ray diffraction (sc-XRD) analysis (Fig. 2). All azadistibiridines feature a three-membered NSb_2_-cyclic core, which is orthogonal to the *trans*-configured Tbb plane. The N–Sb bond lengths within each ring are nearly identical (2a 2.120(4) & 2.118(3), 2b 2.067(2) & 2.050(2), 2c 2.068(2) & 2.079(2), and 2d 2.069(4) & 2.070(4) Å), and consistent with those of Sb–N single bonds. These distances are also comparable to those reported for the few azadistibiridines in the literature (2.019(2)–2.114(1) Å).^26^ The Sb1–Sb2 bond distances (2a 2.8302(4), 2b 2.8130(3), 2c 2.8254(2), and 2d 2.8732(7) Å) are markedly elongated compared to that of free Sb_2_Tbb_2_ (d(Sb–Sb) = 2.667(1) Å),^29^ but similar to those observed for previously reported three-membered distibacycles (2.7882(2) to 2.8647(5) Å) and acyclic distibanes (∼2.85 Å) in the literature.^16,30,31^ The C^Tbb^–Sb–Sb–C^Tbb^ torsions (2a 169.9(2), 2b 171.8(1), 2c 175.1(1), and 2d 172.3(2)°) are comparable to those observed for methylenedistibiranes (∼172°),^16^ probably reflecting the isolobal relationship between the N(R) and the C(NHC) fragments.^32,33^ The torsion angles are also similar to that found in a distibene π-complex (173°) with platinum^34^ but significantly larger than those observed in previously reported three-membered (*η*^2^)-distiba-metallacycles (∼155–160°).^31^

All azadistibiridines exhibit notable variation in the degree of pyramidalisation at the nitrogen centre. This is evident from the sum of bond angles (Σ∡N) around the nitrogen (2a 325.4, 2b 357, 2c 345.9, and 2d 348.2°). In 2a, the nitrogen centre adopts a more pyramidal configuration, whereas in 2b, it approaches planarity and in 2c as well as 2d, it stays in the quasi-pyramidal state. This trend is attributed to the substituent effects on the nitrogen centre and, most likely, to the extent of p(N) → σ* donation.^35^

Despite adopting *C*_1_-symmetry in the solid-state, with the pyramidal nitrogen centre, compounds 2a, 2c, and 2d display a pseudo *C*_2_-symmetry in solution. This is evidenced by the ^1^H NMR spectrum at 298 K, where only a single set of symmetric signals for both diastereotopic Tbb substituents was observed. This can be attributed to a rapid pyramidal inversion of the nitrogen centre in solution, persisting even at 193 K on the NMR timescale (see SI, Section 7). This is consistent with previous computational studies which reported a relatively low energy barrier (∼5 kcal mol^−1^) for N-inversion in typical aziridines.^36^

To gain more information about the electronic structures, we analysed compounds 2a–2d using density functional theory (DFT) calculations. Single-crystal X-ray-derived structures served as a starting point for the structural optimisation, where applicable. Geometries were first optimised at the TPSS-D4/def2-TZVP level, which incorporates scalar relativistic effects *via* effective core potentials (ECPs) and single-point energy calculations were performed using the double-hybrid PWPB95 functional.^37^ To account for relativistic effects, we employed two different approaches: (1) the ECP-based approach using the def2-TZVP basis set, which incorporates scalar relativistic corrections. (2) The exact-two-component (x2c) approach using the fully uncontracted x2c-QZVPPall basis set, explicitly designed for relativistic calculations. Bond lengths and angles of the computed structures are generally in good agreement with solid-state structures. However, while the pyramidal geometry at the nitrogen atom of 2c and 2d is almost retained (Σ∡N = 345.5° for both), significant differences are found for 2a and 2b. Both angular sums Σ∡N are lower (2a 315.8 and 2b 343.8°) than those in the X-ray structures, suggesting different conformers due to packing effects in the crystal lattice. This points to a high flexibility and low N-inversion barrier, which is in line with the results from ^1^H-NMR spectroscopy.

**Fig. 3:**
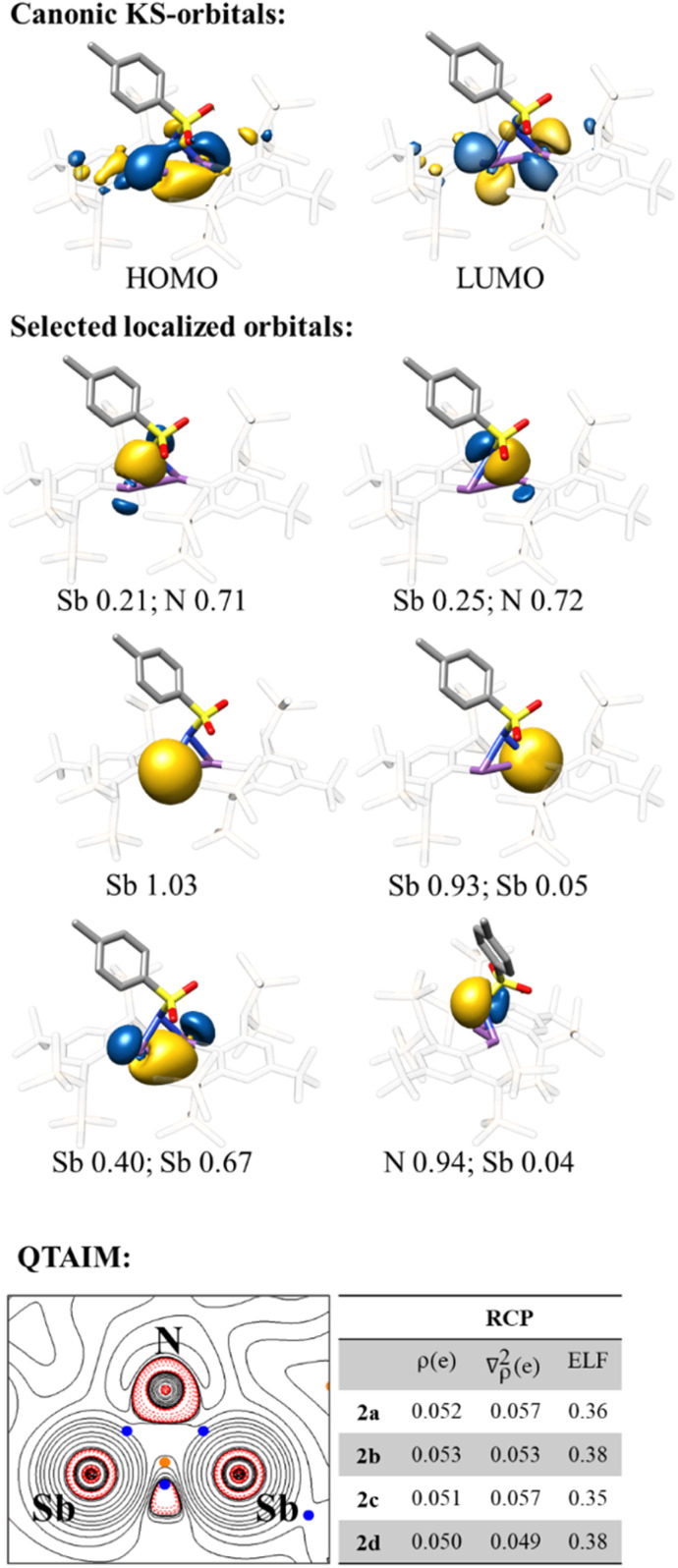
KS-frontier molecular orbitals, selected localised orbitals, Mulliken composition analysis and QTAIM analysis of compound 2a, representative of 2a–d. BCPs are in blue and RCP is in orange. Electron density *ρ* in *e*/*a*_0_^3^, Laplacian of the electron density ∇^2^*ρ* in *e*/*a*_0_^5^ and electron localisation function (ELF) value for 2a–d.

The highest occupied molecular orbital (HOMO) of all azadistibiridines presented is located in the Sb–Sb bond, with a slight bending outwards of the rings, a typical feature of small rings and of the previously reported azadistibiridines (Fig. 3).^26^ Similarly, in all cases the lowest unoccupied molecular orbital (LUMO) is the antibonding variation of the HOMO. HOMO–LUMO gaps are calculated to be between 4.8 and 5.2 eV for all the structures (2a–d). Lower lying DFT derived Kohn–Sham (KS) orbitals are strongly mixed and delocalised, and therefore intrinsic bond orbitals (IBOs) were computed for a more direct visualisation of the bonding situation. The Sb–N bond is less bent outwards, resulting in a more pronounced σ-bond character, in contrast to the Sb–Sb bond.

Mulliken orbital composition analysis clearly showed a bond polarisation to the nitrogen atom. The asymmetry of the ring, due to the N-substituent, leads to an asymmetric Mulliken population of the Sb–Sb IBO, which is most pronounced in 2c, and almost vanishes completely for the more symmetrical adamantyl substituent in 2d. Loewdin orbital population analysis of the IBO, identified as the N-lone pair, showed twice the amount of s-orbital character for 2a (sp^1.4^) compared to that of 2b–d (2b sp^4.6^, 2c sp^3.9^, and 2d sp^3.7^), which fits well with the structural differences previously discussed. QTAIM investigations revealed three bond critical points (BCP) and a ring critical point (RCP) for the cyclic motif. The angle between the Sb atoms and the bond critical point (BCP) is around 163° for 2a–d (see SI for further details). The RCP is significantly shifted from the geometric centre towards the BCP of the Sb–Sb bond. The bent Sb–Sb bond leads to non-negligible charge accumulation outside of the ring. Despite the clear effect of the N-substituent on the nitrogen lone pair character the effect on the ring system is small, according to RCP values, which are qualitatively stable among 2a–d (Fig. 3).

**Fig. 4:**
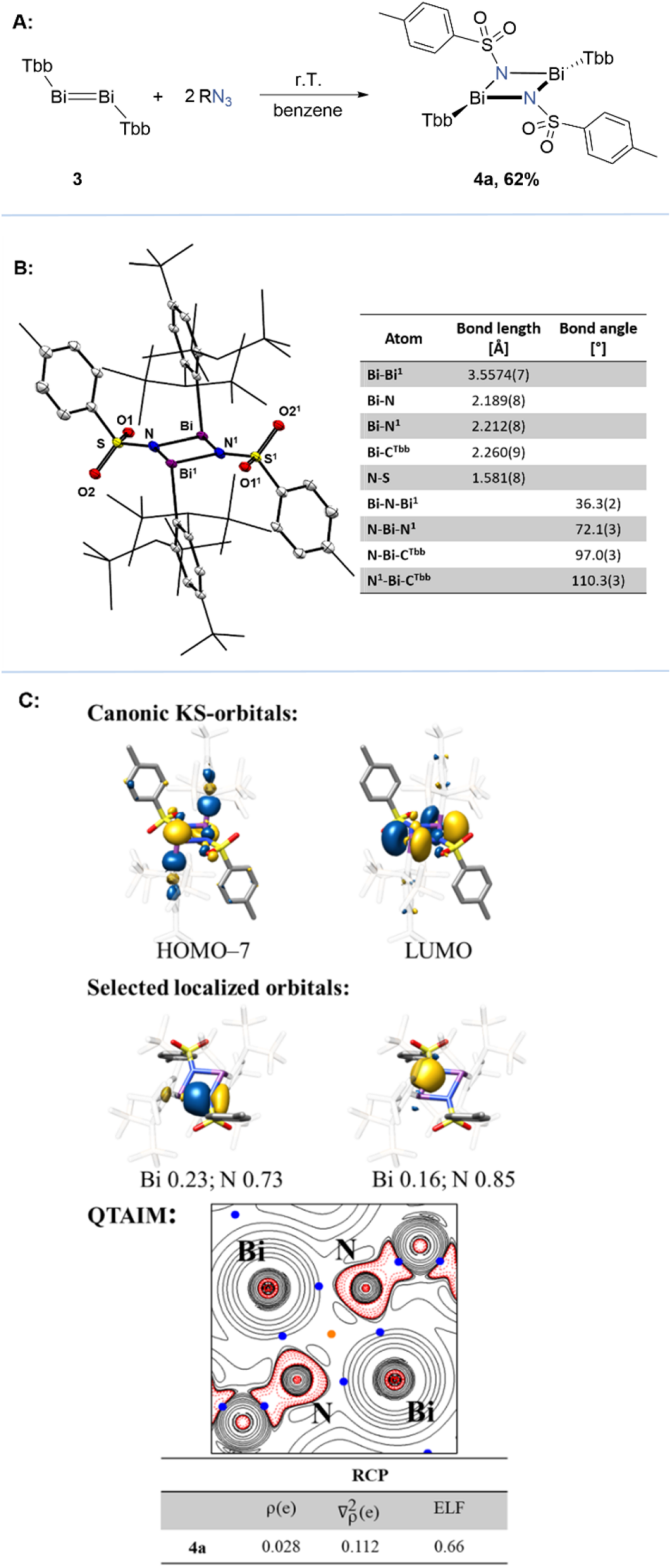
(A) Synthesis of iminobismuthane-dimer 4a; (B) single-crystal X-ray structures of compound 4a and a table of selected bond parameters. Displacement ellipsoids are drawn at 30% probability level. Hydrogen atoms are omitted for clarity (C) KS-frontier molecular orbitals, selected localised orbitals, and Mulliken and QTAIM analysis of 4a. BCPs are in blue and RCP is in orange. Electron density *ρ* in *e*/*a*_0_^3^, Laplacian of the electron density ∇^2^*ρ* in *e*/*a*_0_^5^ and electron localisation function (ELF) value for 4a.

Despite the great advances in bismuth chemistry,^38–41^ activating the “Bi=Bi” double bond remains an open challenge.^40–43^ Pronounced relativistic effects contribute to bond contraction, and elevating the energy of the π* orbital, further stabilizing the double bond.^44^ Driven by curiosity, we decided to apply our protocol to dibismuthene. Surprisingly, only tosyl azide showed promising reactivity, while all the other azides either remained unreactive even at elevated temperatures or showed formations of multiple products. Even modification of the ligand framework at the bismuth centre did not lead to further success. For instance, the bulky azide (Ar^Mes^–N_3_) was reacted with various dibismuthenes, including Bi_2_Tbb_2_, Bi_2_Bbt_2_, and Bi_2_Ar^Mes^_2_, but no reaction occurred, neither at elevated temperatures (up to 100 °C) nor under visible light irradiation (up to 451 nm). Exposure to UV light (367 nm) resulted in decomposition of the azide, while the dibismuthenes remained largely unaltered. However, upon treating a suspension of Bi_2_Tbb_2_ in benzene with a stock solution of tosyl azide at room temperature, a bright yellow suspension was formed overnight (Fig. 4A). After work-up, 4a was isolated as a yellow amorphous solid in an analytically pure form with a yield of 62%. Notably, the reaction of 3 with one equivalent of tosyl azide resulted in a selective formation of 4a, with nearly stoichiometric amounts of unreacted Bi_2_Tbb_2_. Single-crystal X-ray diffraction unambiguously confirmed product 4a as a diazadibismetidine, synthesised *via* “Bi=Bi” double bond cleavage by using an azide at room temperature. While few examples of such compounds are known,^12,13,17^ this stands as the first example of a dibismuthene reaction with a 1,3-dipolar reagent. In the solid-state molecular structure (Fig. 4B), 4a features a planar four-membered core (∡N–Bi–Bi^1^–N^1^ = 180°) with two distorted pyramidal Bi (Σ∡Bi = 279.4°) centres and two planar N (Σ∡N = 359.8°) centres. The Bi–N bond lengths (2.212(8) and 2.189(8) Å) are comparable to that of a typical single bond (Σ*r*(Bi–N)_cov._ = 2.20 Å)^45^ and fall within the range previously reported for few known diazadibismetidines (∼2.129 to 2.213 Å) in the literature.^12,13,17,19^ Beyond direct interactions, the electropositive Bi centres exhibit weak intramolecular secondary coordination with the O1 atom of the tosyl ligand's sulfonyl group, as evidenced by the O1–S–N angle, showing an inclination of O1 towards Bi (105.5(4)°). This Bi–O1 distance (2.90(1) Å) is significantly longer than a typical Bi–O single bond (Σ*r*(Bi–O)_cov._ = 2.14 Å)^45^ but considerably shorter than the sum of van der Waals radii for Bi and O (3.59 Å)^46^ and the Bi–O distances (∼3.1 Å) observed in Bi–O containing cluster compounds found in the CSD database.

Compound 4a was further analysed by DFT. Localised orbitals show four well defined Bi–N σ-bonds, which are polarised towards the N-atoms, according to Mulliken orbital composition analysis (Fig. 4C). No significant outwards bending of the bonds is observed, compared to 2a. As expected from the trigonal planar environment at the N-atoms, the N-lone pairs are p(N)-orbitals with a negligible amount of s(N)-orbital contribution. QTAIM analysis gave four BCP and one RCP for the central cyclic motive. The latter is located in the geometric centre of the four membered ring, as expected due to the high symmetry. From canonical KS-orbital analysis it can be expected that 4a reacts as a weak nucleophile; however, the dominant p(Bi)-orbital character of the LUMO suggests a significantly high Lewis acidity at the Bi centres.

**Scheme 2:**
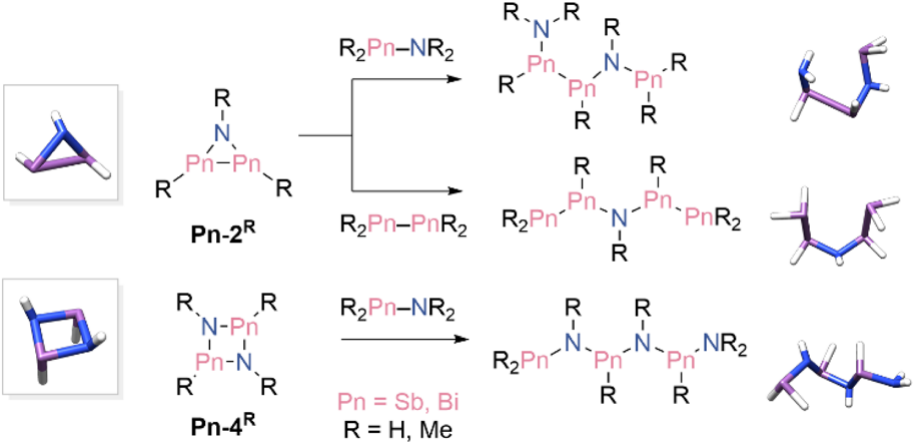
Lewis structure of the three-and four-membered cyclic model compounds Pn-2R and Pn-4R and their homodesmotic bond cleavage products from which RSEs were calculated. Computed structures are shown for R=H as representatives.

The resulting different ring sizes in the reaction of 1 and 3 with organic azides prompted us to investigate the ring strain energies (RSEs) of the final products, to better understand the origin of this phenomenon (Scheme 2). Values for the RSE were obtained through homodesmotic calculations. Remarkably, RSEs of model compounds (Scheme 2, R=H, Me) 2 and 4 were very close (Sb-2^H^ 19.2, Sb-2^Me^ 17.7, Bi-4^H^ 21.1, and Bi-4^Me^ 21.0 kcal mol^−1^) and consistent with previously reported values.^47,48^ Furthermore, no significant differences in the RSE of the not experimentally accessed azadibismiridines Bi-2^R^ and 1,3,2,4-diazadistibetidines Sb-4^R^ were found (Bi-2^H^ 18.4; Bi-2^Me^ 19.5; Sb-4^H^ 21.3; Sb-4^Me^ 20.4; values in kcal mol^−1^). Hence, it can be concluded that the reaction outcome is not affected by the ring strain of the cyclic products.

To further understand the different outcome between distibene and dibismuthene, we have also analysed the thermodynamics of the mechanism using three different levels of theory (see SI for further details). We identified two main pathways one leading to the three-membered ring and the other to the four-membered ring (See SI, Fig. S16 and S17). Unfortunately, even in this case, the difference between the two is not significant, suggesting that the determining factor may lie in the kinetics rather than thermodynamic parameters.

**Fig. 5:**
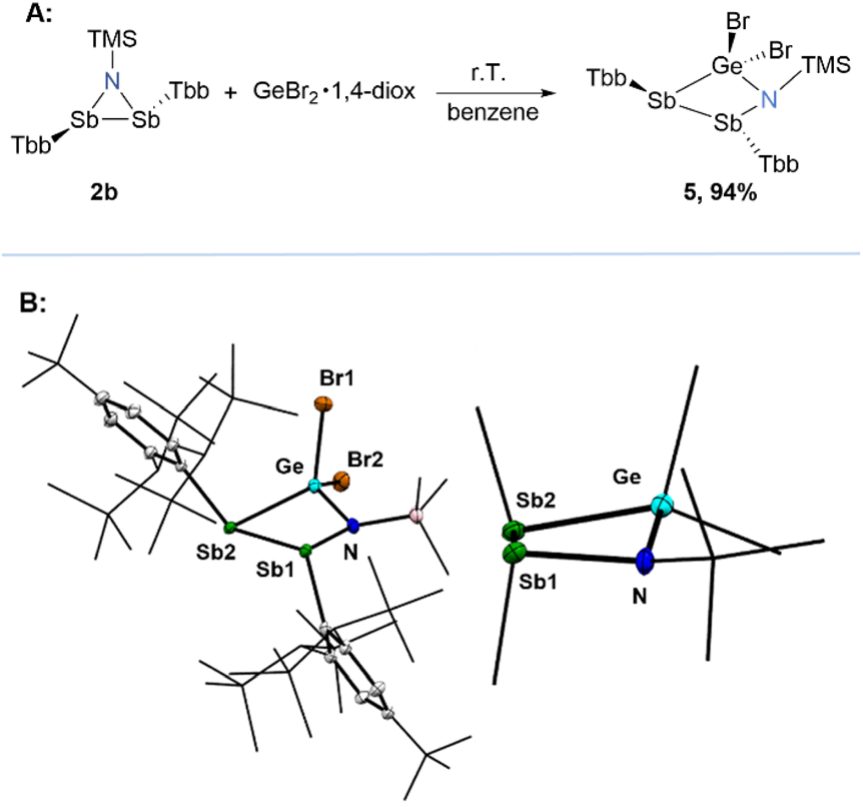
(A) GeBr_2_ insertion reaction into one of the N–Sb bonds of azadistibiridine 2b; (B) single-crystal X-ray structure of compound 5 and its condensed side-view. Displacement ellipsoids are drawn at 30% probability level. Hydrogen atoms are omitted for clarity.

We were then interested in exploring the chemical space and the potential reactivity of these unusual structures. Azadistibiridines are highly electron-rich, small, strained rings with multiple reactive sites due to their polarised Sb–N bonds, making them excellent candidates for ring expansion reactions. Among them, compound 2b, the least sterically hindered derivative, was selected as a model system to investigate its reactivity toward electrophiles. Several reactions were evaluated with electrophilic reagents such as MeI, CO_2_, small organic aldehydes, AlCl_3_, SbCl_3_, and SnBr_2_ which remained mostly unreactive or very unselective leading to formation of multiple products. However 2b readily reacts with GeBr_2_ at room temperature. When a yellow benzene solution of 2b was treated with a colourless suspension of GeBr_2_·1,4-dioxane in benzene, the reaction mixture gradually changed colour to orange-yellow overnight (Fig. 5A). After work-up, compound 5 was isolated in 94% yield as an air-sensitive yellow powder. It was completely characterised by elemental analysis, NMR and UV/vis spectroscopies, as well as single-crystal X-ray diffraction (sc-XRD) analysis. While several diaza-heterocycles have been studied in the literature,^49–52^ compound 5 represents a rare example of a four-membered “distiba”-heterocycle where germanium dibromide formally inserts into one of the Sb–N bonds. The solid-state structure (Fig. 5B) features a puckered four-membered ring with an interflap angle (∡N–Ge–Sb1–Sb2) of −148.7(2)°, and two trigonal pyramidal coordinated Sb centres (Σ∡Sb(1) = 288.5°; Σ∡Sb(2) = 287.9°), one bonded to germanium and the other to the nitrogen atom. The Sb–Ge distance of 2.5989(6) Å falls within the typical range for a single bond (Σ*r*(Sb–Ge)_cov._ = 2.61 Å),^45^ closely matching the Sb–Ge bond length (2.636(1) Å) reported for a (trimethyl)-germyl substituted distibane in the literature.^53^ The Sb–N bond distance (2.098(4) Å) is comparable to that of its precursor. Interestingly, the Sb1–Sb2 distance (2.9274(4) Å) is markedly elongated compared to that of its precursor (∼0.12 Å), probably due to the release of ring strain upon expansion. Furthermore, this distance is also elongated (∼0.08 Å) compared to those found in five-membered Sb_2_-heterocycles,^16,24^ but quite similar to those observed for a few structurally strained and sterically encumbered distibanes (∼2.95 Å).^54–57^

Compound 5 has a *C*_1_-symmetric structure with two heterotopic Tbb substituents both in the solid-state and in solution. However, in solution a hindered rotation of the C^Tbb^–Sb bond was observed for one of the Tbb substituents, as evidenced by ^1^H-NMR spectroscopy at room temperature, which featured one set of sharp signals corresponding to the freely rotating Tbb group and very broad resonances for the other faces. Upon cooling to 193 K all bond rotations were completely frozen out on the NMR time scale, giving two discrete sets of signals for two heterotopic Tbb groups.

**Fig. 6:**
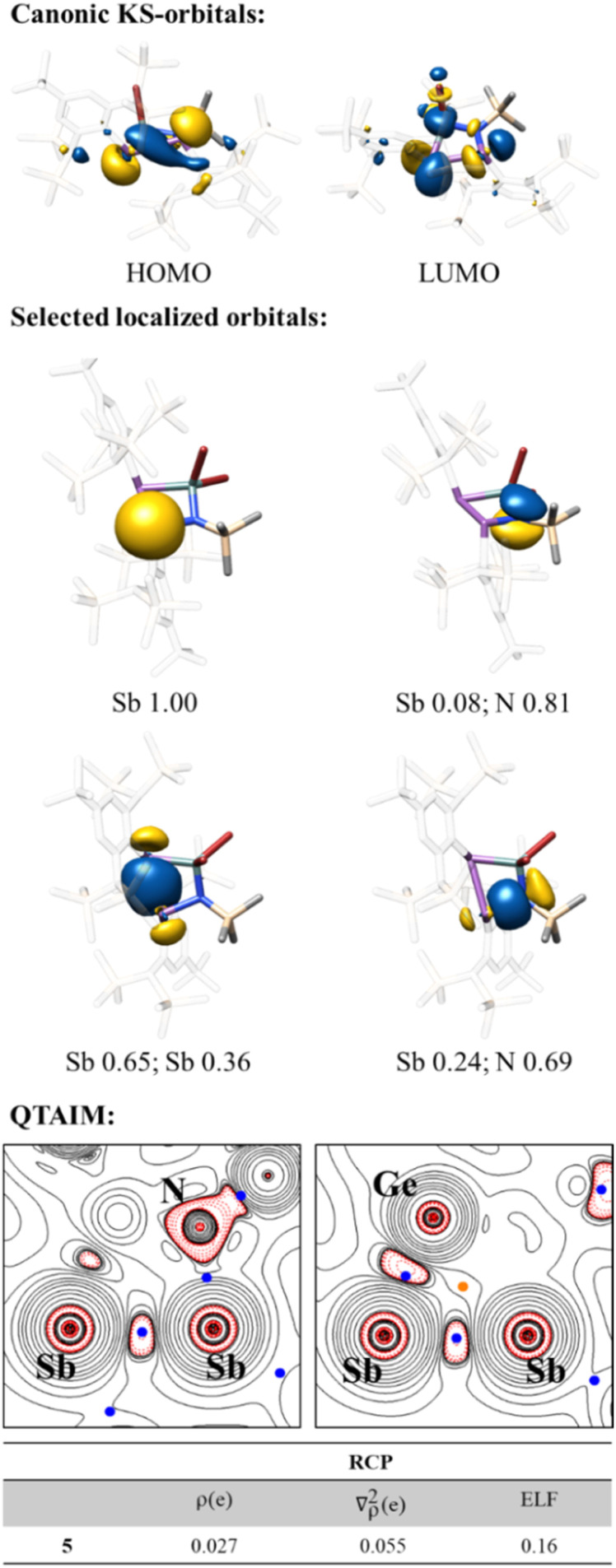
KS-frontier molecular orbitals, selected localised orbitals and Mulliken composition analysis and QTAIM analysis of 5. BCPs are in blue and RCP is in orange. Electron density *ρ* in *e*/*a*_0_^3^, Laplacian of the electron density ∇^2^*ρ* in *e*/*a*_0_^5^ and electron localisation function (ELF) value for 5.

Due to the unusual molecular structure of compound 5, we decided to further investigate the electronic structure by DFT calculations. Ring expansion by GeBr_2_ reduces the outward bending of the Sb–Sb bond, according to results from localised orbital analysis (Fig. 6). The asymmetry of the four-membered ring causes a Sb–Sb bond polarisation towards the nitrogen bound Sb centre, regardless of a strong polarisation of the Sb–Ge bond in favour of the Ge atom. The Sb-lone pairs retain a huge s(Sb)-orbital character (sp^0.4^), while the N-lone pair has slightly increased p(N)-orbital character (sp^5.5^) compared to 2b.

The RCP obtained from QTAIM analysis is significantly more shifted to the geometric centre compared to the situation in 2b. In line with the less outward bending of the Sb–Sb bond the charge accumulation between the Sb centres is more symmetric around the Sb–Sb axis. Remarkably, charge accumulation is also found along the Sb–Ge axis, but shifted towards the Ge atom in line with the polarisation of the Sb–Ge bond. Electron density and electron localisation function (ELF) values at the RCP are lower compared to those of 2b. The HOMO of 5 is dominated by the Sb lone pairs with a minor σ(Sb–Sb) contribution, while the LUMO has Sb–Sb and Sb–Ge antibonding character.

**Fig. 7:**
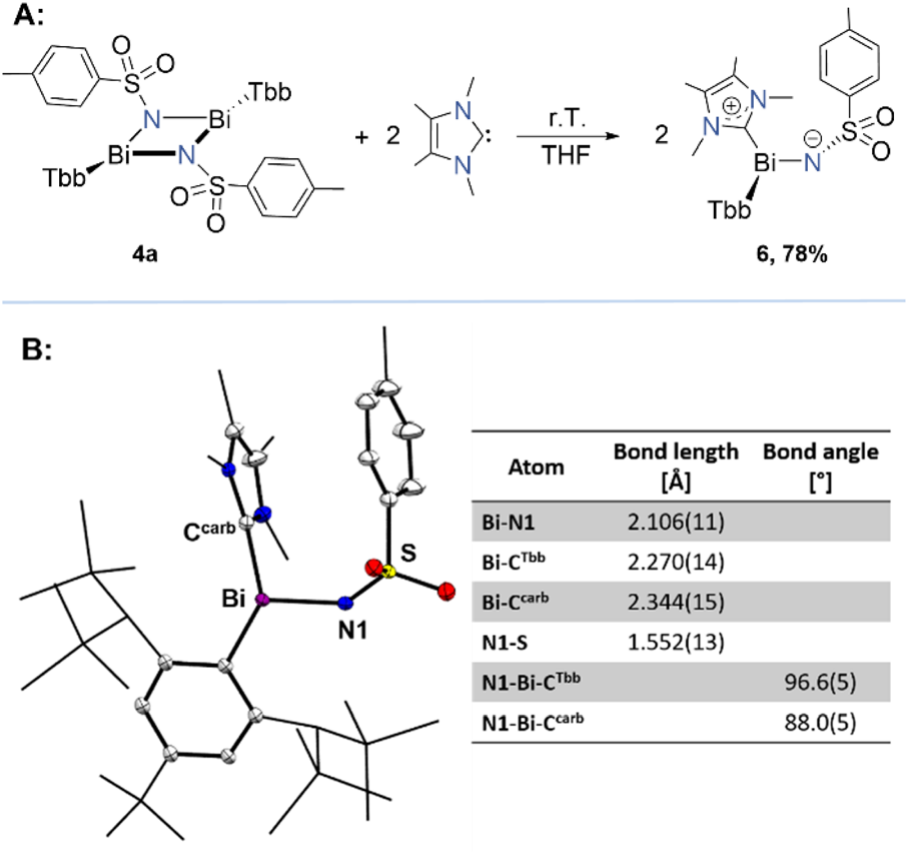
(A) Synthesis of 6*via* IMe_4_ addition to 4a; (B) single-crystal X-ray structure of compound 6 and a table for some selected bond parameters. Displacement ellipsoids are drawn at 30% probability level. Hydrogen atoms are omitted for clarity.

Next, we wanted to explore the potential divergent reactivity of our bismuth-based four-membered ring. Notably, we attempted the reaction of 4a with electrophiles such as CO_2_ or even GeBr_2_; however, no reaction was observed, even at elevated temperatures (100 °C). Our assessment of the frontier molecular orbitals of 4a shows that the HOMO is primarily ligand-based, while the LUMO corresponds to a p-like orbital of the Bi–N bond, with a greater contribution from the bismuth centre. Thus, we envisaged 4a as a perfect candidate to react with nucleophiles, potentially converting it into a monomer upon reaction with a suitable donor. However, it did not show any reactivity towards phosphine (PPh_3_) or an amine (DABCO) even at elevated temperature. Then we turn back to NHCs, assuming they might be a perfect match due to their strong donating ability as often reported in low-valent main group chemistry.^58,59^ To test this hypothesis, we performed the reaction of 4a with 1,3,4,5-tetramethylimidazol-2-ylidene (IMe_4_) (Fig. 7A). Upon addition of THF to a mixture of solids, we observed an immediate formation of a pale-yellow solution. ^1^H NMR analysis of the reaction aliquot confirmed the complete consumption of the starting materials and a very selective formation of a new product. We isolated compound 6 as an amorphous beige solid in 78% yield. It was completely characterised by elemental analysis, and NMR and UV/vis spectroscopies, as well as single-crystal X-ray diffraction (sc-XRD) analysis. The crystal structure confirmed the identity of 6 as a monomer and may be considered as a rare example of an NHC-supported iminobismuthane (Fig. 7B). The solid-state structure of compound 6 shows the following key structural features: (1) a *trans*-bent hetero-dipnictene core with the ligands (Tbb and Ts) adopting an anti-periplanar arrangement, as evidenced by the torsion angle (∡C^Tbb^–Bi–N–S = −168.6(7)°), (2) a trigonal pyramidal coordinated Bi centre (Σ∡Bi = 283°), connected to a di-coordinated V-shaped N centre *via* a short Bi–N bond (2.106 (11) Å), and (3) a carbene bound to the Bi centre (Bi–C^carb^ = 2.344(15) Å), oriented orthogonal to the C^Tbb^–Bi–N plane. The Bi–N bond length is considerably shorter (∼0.1 Å) than that of its precursor 4a but closely resembles those reported for Bi=N bonds in bismuth(iii)-phosphoraneiminato complexes (2.12 Å),^60^ and imino-λ^5^-bismuthanes (2.07–2.13 Å) as described by Suzuki and co-workers in the literature.^61,62^ Notably, it is even slightly shorter than the Bi–N bond (2.146(3) Å) recently reported for an iminobismuthane by Cornella and co-workers, where the Bi centre is tetra-coordinated and has additional Lewis-base stabilisation support from its own ligand sphere.^63^ The Bi–C^carb^ (2.344(15) Å) bond distance is comparable to those reported for NHC-stabilised Bi(iii) complexes by Gilliard Jr and co-workers, and Dutton and co-workers.^64–66^ It is worth highlighting that Bi–NHC complexes are relatively rare and cannot be synthesised by simple reaction of dibismuthene and a carbene. The *C*_1_-symmetric solid-state structure of complex 6 is also reflected in its solution behaviour. However, hindered rotations around C^Tbb^–Bi and C^carb^–Bi bonds cause slight broadening of some signals in the ^1^H and ^13^C{^1^H}-NMR spectra. Using low-temperature NMR measurements at 243 K, we were able to resolve both proton and carbon spectra. The ^13^C{^1^H}-NMR chemical shift of C^Tbb^Bi for the compound 4a appears as a distinct singlet at 220.4 ppm, which is significantly high field shifted to 175.9 ppm for 6, likely reflecting the increased electron density due to NHC coordination, while Bi remains in the +3-oxidation state.

**Fig. 8:**
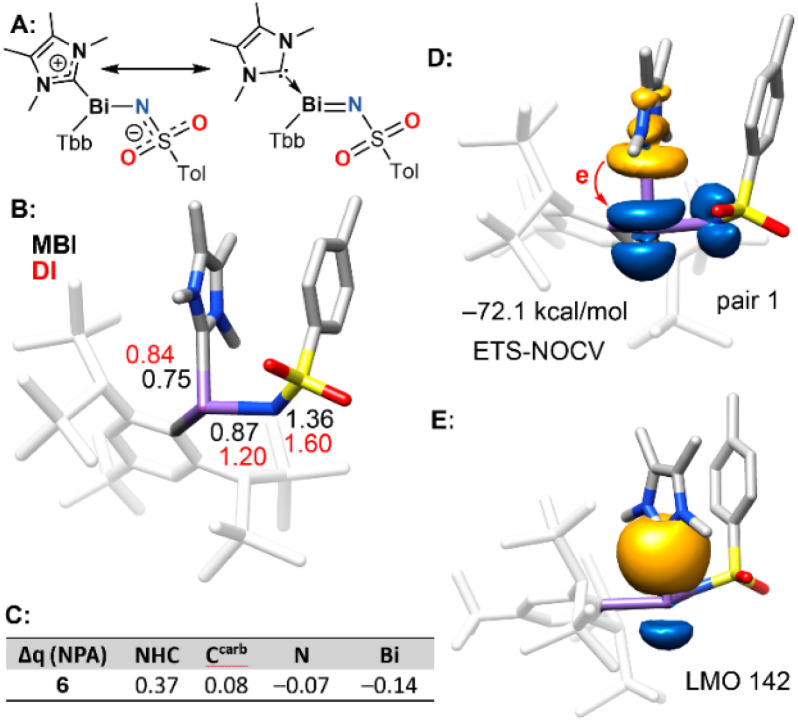
(A) Two possible resonance forms of 6. (B) Optimised geometry of 6 (Tbb moieties as lines), MBI values (in black), and DI values (in red). (C) Table with the Δ*q* values (NPA charges) of N, Bi, C^carb^ and the NHC fragment with respect to isolated Tbb–Bi=N-Ts and NHC. Values in *e*. (D) Plot of the isosurfaces of the largest NOCV pair density for the C^carb^ → Bi electron flow. (E) LMO number 142, corresponding to the σ(Bi–C^carb^) orbital. Level of theory: x2c-PW6B95-D4/x2c-QZVPPall//TPSS-D4/def2-TZVP.

Due to the peculiarity of this structure, we decided to further investigate the bonding scenario using DFT calculations, with a particular focus on the nature of the C^carb^–Bi bond using a combination of Extended Transition State-Natural Orbitals for Chemical Valence (ETS-NOCV), Mayer and delocalisation indices (MBI and DI), and the variation of the natural population analysis (NPA) charges (Δ*q*) in 6 with respect to isolated NHC and Tbb–Bi=N–Ts compounds. The bond orders, Δ*q* values and localised orbital analysis were employed to differentiate between the two possible resonance structures, one with a zwitterionic N–Bi single bond and another with a Bi=N double bond (Fig. 8A). The unique Lewis structure obtained from the NRT analysis is the one on the left, where both the Bi–C^carb^ and Bi–N bonds exhibit single bond character. This is corroborated by MBI and DI bond orders, which are close to 1 for both bonds. Notably, the DI values for Bi–C^carb^ and Bi–C^Tbb^ are very similar (0.84 and 0.85, respectively). Interestingly, both MBI and DI values suggest partial double bond character in the N–S bond (Fig. 8B), indicating that the partial negative charge on the nitrogen atom is delocalised into the tosyl group.

The charge analysis (Δ*q*, Fig. 8C) supports the zwitterionic Lewis structure, revealing an increase in the negative charge on the nitrogen atom and a corresponding increase in the positive charge on the NHC fragment, including the C^carb^ atom. Additionally, the Δ*q* value at the bismuth atom is negative, consistent with electron transfer from the NHC to the Bi atom. This interpretation is further confirmed by the ETS-NOCV analysis, which considers the NHC as one fragment and the iminobismuthane (Tbb–Bi=N–Ts) moiety as the other. The results showcased unidirectional electron donation from the NHC to the Bi atom (Fig. 8D). The ETS-NOCV deformation density isosurface corresponding to this charge transfer clearly shows electron flow from the NHC carbon lone pair into the π*(Bi=N) orbital of the Tbb–Bi=N–Ts fragment. The energy associated with this NOCV pair is −72.1 kcal mol^−1^ that is more consistent with the covalent nature of the Bi–C^carb^ bond rather than a dative bond. Additionally, the computed bond dissociation energy (BDE) for the Bi–C^carb^ bond in compound 6 is 46.5 kcal mol^−1^, which is comparable to the BDE of the reference covalent bond (CH_3_)_2_Bi–CH_3_ (50.2 kcal mol^−1^).^67^ According to the Haaland criterion and considering that similar bond energies do not necessarily imply similar bonding situations, the comparable bond strength supports classification of the Bi–C^carb^ interaction as a covalent bond rather than a dative bond.^68^ Additionally, we were also able to find the localised molecular orbital (LMO) corresponding to the σ(Bi–C^carb^) bond (Fig. 8E). The electronic transitions of all the compounds have been analysed and correlated with the experimental data, including the composition and transitions of each band (see SI, Table S5).

## Conclusions

In conclusion, we have reported the synthesis and characterisation of small heterocyclic rings containing Sb_2_-or Bi_2_ units, synthesised *via* cycloaddition reactions of a distibene or a dibismuthene with organic azides. Interestingly, the reaction with dibismuthene led to an unprecedented reactivity with the formation of a four-membered ring in strong contrast to the distibene. The structures have been thoroughly investigated by computational methods which have guided the reactivity studies. Thanks to the ring strain, we have been able to promote ring expansion of the azadistibiridine 2b by germanium dibromide insertion in the Sb–N bond. With a similar strategy, starting from the bismuth complex 4a but using a nucleophile instead (IMe_4_), we have been able to promote formation of the first NHC-stabilised hetero-dipnictene. Analysis of the electronic features suggested its best representation to be a zwitterion. These two examples represent just a small glimpse of the possibilities offered by small inorganic rings, and how we can use their unique properties to create new and complex molecular structures that would otherwise be difficult or impossible to achieve.

## Author contributions

P. P. and A. B. conceived the idea behind the manuscript. P. P., M. B., and D. M. performed the experimental work and spectroscopic studies. P. B., R. M. G., and A. F. performed the theoretical calculations. G. S. performed the crystallographic measurements and solved the structures. The manuscript was written through the contributions of all authors. All authors have approved the final version of the manuscript.

## Conflicts of interest

There are no conflicts to declare.

## Supplementary Material

SC-016-D5SC03416G-s001

SC-016-D5SC03416G-s002

SC-016-D5SC03416G-s003

## Data Availability

All the experimental procedures, the spectroscopic data, the computational studies, and detailed crystallographic information have been included as part of the SI. See DOI: https://doi.org/10.1039/d5sc03416g. 2440170 (2a), 2440171 (2b), 2440172 (2c), 2440173 (2d·(Et_2_O)), 2440174 (4a), 2440175 (5·(Et_2_O)_0.5_), and 2440176 (6) contain the supplementary crystallographic data for this paper.^69–75^
