# Supplementary material for: Azadistibiridines and stabilised-iminobismuthane: reactivity of small inorganic rings in heavy main group chemistry
Source: Chem Sci. 2025 Aug 19;16(36):16894–903. doi: 10.1039/d5sc03416g (PMC12378412; doi:10.1039/d5sc03416g)
Supplement: SC-016-D5SC03416G-s002 [file SC-016-D5SC03416G-s002.pdf]

Supplementary Information (SI) for Chemical Science.
This journal is © The Royal Society of Chemistry 2025

Azadistibiridines and Stabilised-iminobismuthane: Reactivity of Small Inorganic Rings in Heavy Main Group Chemistry

Prasenjit Palui, Matthias Bollenbeck, Daniel Meleschko, Phillipp Brehm, Rosa M. Gomila, Gregor Schnakenburg, Antonio Frontera, and Alessandro Bismuto*

*bismuto@uni-bonn.de

Table of Contents

1. General information ... 1
2. Syntheses of Azadistibiridines, *Cyclo*-dibismadiazene, and NHC-supported iminobismuthane .. 2
2.1. Synthesis of complex **2a** ... 2
2.2. Synthesis of complex **2b** ... 3
2.3. Synthesis of complex **2c** ... 4
2.4. Synthesis of complex **2d** ... 5
2.5. Synthesis of complex **4a** ... 6
2.6. Synthesis of compound **5** ... 7
2.7. Synthesis of compound **6** ... 8
3. UV/Vis Spectroscopic Studies ... 10
4. Computational methods ... 16
4.1. Additional material: Azadistibiridines **2a**, **2b**, **2c** and **2d** ... 17
4.2. Additional material: Canonical Orbitals of compound **5** ... 21
4.3. Additional material: Outwards bond bending in selected three-membered rings ... 21
5. Mechanistic study, ETS-NOCV and TD-DFT results ... 23
5.1. Mechanistic studies ... 23
5.2. Canonical and localised molecular orbitals of compound **6** ... 26
5.3. ETS-NOCV analysis ... 28
5.4. TD-DFT results ... 30
6. Single crystal X-ray diffraction analysis ... 31
6.1. General ... 31
7. NMR spectra ... 37
8. References ... 48

1. General information

All experiments were carried out in a glovebox (UNILAB, MBraun) under an argon atmosphere or using standard Schlenk techniques. *n*-Hexane was collected from a solvent purification system (MBraun MB-SPS7), degassed through freeze-pump-thaw cycles and stored over molecular sieves (3 Å) prior to use. Benzene, tetrahydrofuran, diethyl ether and *n*-pentane were dried over sodium and were distilled and stored over molecular sieves (3 Å) prior to use. Acetonitrile was distilled upon stirring over CaH_2 and stored over molecular sieves (3 Å) prior to use.

Unless otherwise specified, all reagents were used as received from commercial suppliers (ABCR, AcrosOrganics, Alfa Aesar, BLDpharm, Sigma Aldrich, Thermo Fisher Scientific). All pnictogen halides PnX_3 were purified by sublimation under reduced pressure and stored under Ar atmosphere prior to use. Bi_2Tbb_2,[1] Sb_2Tbb_2,[1] $Bi_2Ar^{Mes}{}_2$,[2] Bi_2Bbt_2,[3] Ar^{Mes}-N_3,[4] $GeBr_2$•1,4-dioxane,[5] and IMe_4[6] were synthesised according to literature.

^{1}H and ^{13}C{^{1}H} NMR spectra were recorded in C_6D_6 or THF-d_8 at 298 K (otherwise specified) using a Bruker Avance I 300 MHz, Bruker Avance I 400 MHz, Bruker Avance III HD Ascend 500 MHz and Bruker Avance III HD Ascend 700 MHz. ^{1}H NMR was reported as follows: chemical shift, multiplicity (s = singlet, d = doublet, and t = triplet), coupling constant (*J* values) in Hz and integration. Chemical shifts (δ) were reported with respect to the corresponding solvent residual peak at 7.16 ppm for C_6D_6 and 3.58 ppm for THF-d_8. ^{13}C NMR spectra (^{1}H-broadband decoupled) were reported in ppm using the central peak of C_6D_6 (128.08 ppm) or THF-d_8 (67.57 ppm). UV/Vis spectra were recorded with a JASCO V-750 UV-Visible Spectrophotometer at room temperature using a 10 × 10 mm quartz sample cell sealed with a J. Young valve.

2. Syntheses of Azadistibiridines, *Cyclo*-dibismadiazene, and NHC-supported iminobismuthane

2.1. Synthesis of complex 2a

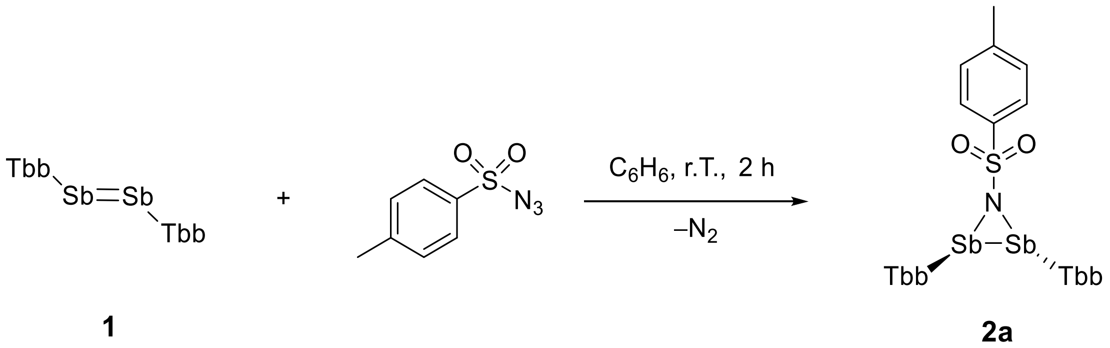

A yellow-orange suspension of Sb_2Tbb_2 (500 mg, 0.438 mmol, 1.00 equiv.) in benzene (25.0 mL) was treated with a solution of tosyl azide (0.85 mL, 0.460 mmol, 1.05 equiv.) in benzene (c = 0.543 M) while stirring at room temperature for 2 h. Over time, the yellow-orange suspension gradually changed into a yellow solution. Analysis of an aliquot of the reaction mixture by ^{1}H NMR spectroscopy confirmed the complete consumption of Sb_2Tbb_2 and selective formation of complex **2a**. The solvent was evaporated under reduced pressure, followed by crystallisation of the yellow crude product from *n*-pentane (4.0 mL) at –30 °C. Yellow crystals were isolated by filtration at –30 °C and dried at room temperature under reduced pressure to give complex **2a** as a yellow crystalline solid (400 mg, 70% yield).

Compound **2a** is an air-sensitive yellow crystalline solid. It can be stored under argon atmosphere at –30 °C for several months. It shows very good solubility in benzene, THF, *n*-pentane and *n*-hexane and insoluble in MeCN at room temperature. A sample solution of **2a** in C_6D_6 does not show any appreciable decomposition even after 5 hours at 85 °C.

^{1}H NMR (500 MHz, C_6D_6): δ 8.02 (m, 2H, Ts), 7.00 (s, 4H, $C^{3,5}$-*H*, Tbb), 6.84 (m, 2H, Ts), 2.95 (s, 4H, Dsi-*H*, Tbb), 1.91 (s, 3H, C^4-*Me*, Ts), 1.28 (s, 18H, C^4-C*Me*$_3$, Tbb), 0.32 (s, 36H, Si*Me*$_3$, Tbb), 0.26 (s, 36H, Si*Me*$_3$, Tbb).

^{13}C{^{1}H} NMR (126 MHz, C_6D_6): δ 151.4, 151.1, 147.8, 142.7, 141.5, 128.4, 127.7, 122.8, 34.5, 34.2, 31.2, 21.1, 1.4, 1.2.

Elemental analysis: Calcd. in % for $C_{55}H_{105}NO_2SSb_2Si_8$ (1312.70 g/mol): C 50.32, H 8.06, N 1.07, S 2.44; $C_{55}H_{105}NO_2SSb_2Si_8$•0.5 C_5H_{12} (1348.77 g/mol): C 51.20, H 8.30, N 1.04, S 2.43; found: C 50.71, H 7.91, N 1.13, S 2.43.

2.2. Synthesis of complex 2b

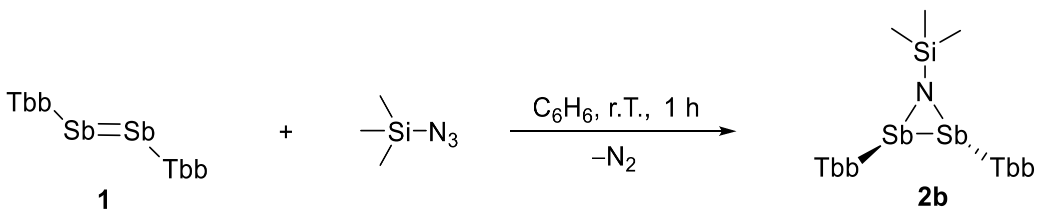

A yellow-orange suspension of Sb_2Tbb_2 (500 mg, 0.438 mmol, 1.00 equiv.) in benzene (25.0 mL) was treated with a stock solution (0.423 M in *n*-hexane) of trimethylsilyl azide (1.15 mL, 0.482 mmol, 1.10 equiv.) at room temperature and stirred for 1 h. Over time, the yellow-orange suspension gradually changed into a clear yellow solution. Analysis of an aliquot of the reaction mixture by ^{1}H NMR spectroscopy confirmed the complete consumption of Sb_2Tbb_2 and a very selective formation of complex **2b**. The reaction mixture was worked-up by evaporating the solvent under reduced pressure, followed by washing the yellow crude product with a mixture of MeCN/Et_2O (4:1, *v/v*) (2 x 10.0 mL). Drying the residue under reduced pressure gave complex **2b** as a yellow amorphous solid (490 mg, 91% yield).

Compound **2b** is an air-sensitive yellow amorphous solid. It can be stored under argon atmosphere at –30 °C for several months. It shows very good solubility in THF, DCM, benzene, *n*-hexane and Et_2O but insoluble in MeCN at room temperature. A sample solution of **2b** in C_6D_6 does not show any appreciable decomposition even after 5 hours at 85 °C.

^{1}H NMR (500 MHz, C_6D_6): δ 6.91 (s, 4H, $C^{3,5}$-*H*, Tbb), 2.89 (s, 4H, Dsi-*H*, Tbb), 1.29 (s, 18H, C^4-C*Me*$_3$, Tbb), 0.49 (s, 9H, N-Si*Me*$_3$), 0.32 (s, 72H, Si*Me*$_3$, Tbb).

^{1}H NMR (400 MHz, THF-d_8): δ 6.76 (s, 4H, $C^{3,5}$-*H*, Tbb), 2.68 (s, 4H, Dsi-*H*, Tbb), 1.27 (s, 18H, C^4-C*Me*$_3$, Tbb), 0.23 (s, 9H, N-Si*Me*$_3$), 0.17 (s, 36H, Si*Me*$_3$, Tbb), 0.16 (s, 36H, Si*Me*$_3$, Tbb).

^{1}H NMR (300 MHz, THF-d_8, 193 K): δ 6.70 (s, 4H, $C^{3,5}$-*H*, Tbb), 2.63 (s, 4H, Dsi-*H*, Tbb), 1.26 (s, 18H, C^4-C*Me*$_3$, Tbb), 0.17 (s, 9H, N-Si*Me*$_3$), 0.16 (s, 36H, Si*Me*$_3$, Tbb), 0.14 (s, 36H, Si*Me*$_3$, Tbb).

^{13}C{^{1}H} NMR (126 MHz, C_6D_6): δ 150.4, 150.1, 148.5, 122.7, 34.4, 32.7, 31.3, 6.9, 1.6, 1.4.

Elemental analysis: Calcd. in % for $C_{51}H_{107}NSb_2Si_9$ (1230.69 g/mol): C 49.77, H 8.76, N 1.14; found: C 49.86, H 8.76, N 1.17.

Note: Due to overlapping of the signals corresponding to Si*Me*$_3$ from the Tbb groups only one signal corresponding to two diastereotopic Si*Me*$_3$ groups is observed in ^{1}H NMR spectrum.

2.3. Synthesis of complex 2c

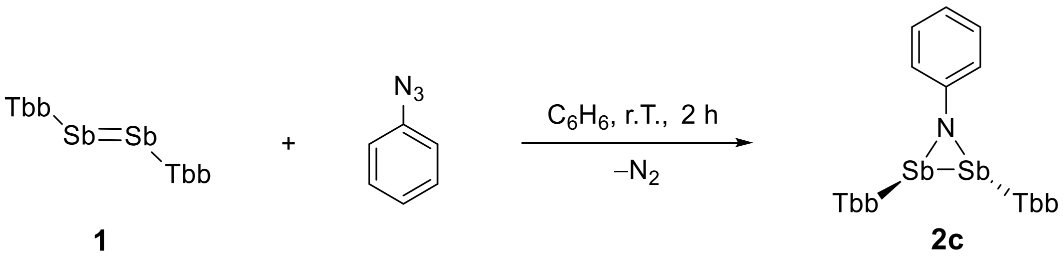

A yellow-orange suspension of Sb_2Tbb_2 (500 mg, 0.438 mmol, 1.00 equiv.) in benzene (20.0 mL) was treated with a stock solution (0.5 M in *tert*-butylmethyl ether) of phenyl azide (0.96 mL, 0.048 mmol, 1.10 equiv.) at room temperature and stirred for 2 h. Over time, the yellow-orange suspension gradually changed into a red solution. Analysis of an aliquot of the reaction mixture by ^{1}H NMR spectroscopy confirmed the complete consumption of Sb_2Tbb_2 and selective formation of complex **2c**. The reaction mixture was worked-up by evaporating the solvent under reduced pressure, followed by crystallisation of the red crude product from Et_2O (4.0 mL) at 4 °C. Dark-red crystals were isolated by filtration and dried at room temperature under reduced pressure to give complex **2c** as a dark-red crystalline solid (365 mg, 68% yield).

Compound **2c** is an air-sensitive red crystalline solid. It can be stored under argon atmosphere at –30 °C for several months. It is insoluble in MeCN, poor soluble *n*-hexane and Et_2O, moderately soluble in benzene, and well soluble in THF at room temperature. A sample solution of **2c** in THF-d_8 does not show any appreciable decomposition even after 5 hours at 65 °C.

^{1}H NMR (500 MHz, THF-d_8): δ 6.92 (m, 2H, Ph), 6.87 (m, 2H, Ph), 6.77 (s, 4H, $C^{3,5}$-*H*, Tbb), 6.62 (m, 1H, Ph), 2.50 (s, 4H, Dsi-*H*, Tbb), 1.25 (s, 18H, C^4-C*Me*$_3$, Tbb), 0.05 (s, 36H, Si*Me*$_3$, Tbb), 0.03 (s, 36H, Si*Me*$_3$, Tbb).

^{1}H NMR (500 MHz, THF-d_8, 193 K): δ 6.94 (t, J_{H-H} = 7.6 Hz, 2H, Ph), 6.78 (d, J_{H-H} = 7.7 Hz, 2H, Ph), 6.72 (s, 4H, $C^{3,5}$-*H*, Tbb), 6.62 (t, J_{H-H} = 7.6 Hz, 1H, Ph), 2.45 (br s, $\Delta\nu_{1/2}$ = 9.3 Hz, 4H, Dsi-*H*, Tbb), 1.23 (s, 18H, C^4-C*Me*$_3$, Tbb), 0.05 (s, 36H, Si*Me*$_3$, Tbb), 0.01 (s, 36H, Si*Me*$_3$, Tbb).

$^{13}C\{^1H\}$ NMR (126 MHz, THF-d_8): δ 151.0, 149.8, 149.5, 144.4, 129.4, 125.0, 122.0, 118.6, 33.6, 31.2, 30.1, 0.0, −0.2.

Elemental analysis: Calcd. in % for $C_{54}H_{103}NSb_2Si_8$ (1234.61 g/mol): C 52.53, H 8.41, N 1.13; found: C 52.60, H 8.47, N 1.15.

2.4. Synthesis of complex 2d

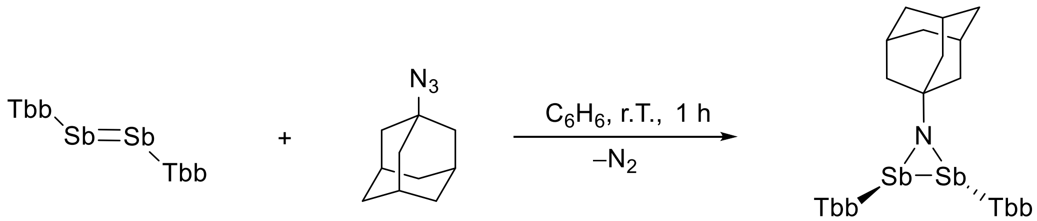

1 **2d**

A yellow-orange suspension of Sb_2Tbb_2 (100 mg, 0.0875 mmol, 1.00 equiv.) in benzene (3.0 mL) was treated with a solution of adamantyl azide (16.3 mg, 0.0919 mmol, 1.05 equiv.) in benzene (3.0 mL) at room temperature and stirred for 1 h. Over time, the yellow-orange suspension gradually changed into a clear orange solution. Analysis of an aliquot of the reaction mixture by ^{1}H NMR spectroscopy confirmed the complete consumption of Sb_2Tbb_2 and selective formation of complex **2d**. The reaction mixture was worked-up by evaporating the solvent under reduced pressure, followed by crystallisation of the orange crude product from Et_2O (1.0 mL) at –30 °C for 6 weeks. Yellowish-orange crystals were isolated by filtration at –30 °C and dried at room temperature under reduced pressure to give complex **2d** as a yellowish-orange crystalline solid (63 mg, 56% yield).

Compound **2d** is an air-sensitive yellowish-orange crystalline solid. It can be stored under argon atmosphere at –30 °C for several months. It is well soluble in benzene, THF, *n*-pentane and *n*-hexane but insoluble in MeCN at room temperature. A sample solution of **2d** in C_6D_6 does not show any appreciable decomposition even after 5 hours at 85 °C.

^{1}H NMR (500 MHz, C_6D_6): δ 6.90 (s, 4H, $C^{3,5}$-*H*, Tbb), 2.94 (s, 4H, Dsi-*H*, Tbb), 2.16-2.06 (m, 6H, Ad), 1.95-1.90 (m, 3H, Ad), 1.77-1.72 (m, 3H, Ad), 1.67-1.61 (m, 3H, Ad), 1.31 (s, 18H, C^4-C*Me*$_3$, Tbb), 0.34 (s, 36H, Si*Me*$_3$, Tbb), 0.31 (s, 36H, Si*Me*$_3$, Tbb).

^{13}C{^{1}H} NMR (126 MHz, C_6D_6): δ 151.3, 150.0, 149.8, 122.6, 55.6, 53.4, 37.0, 34.4, 32.6, 31.7, 31.3, 1.43, 1.35.

Elemental analysis: Calcd. in % for $C_{58}H_{113}NSb_2Si_8$ (1292.75 g/mol): C 53.89, H 8.81, N 1.08; found: C 53.57, H 8.68, N 1.11.

2.5. Synthesis of complex 4a

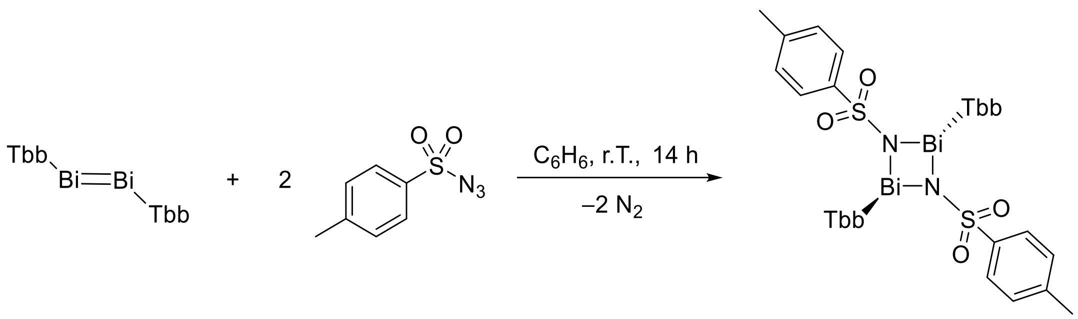

3 4a

A purple suspension of Bi_2Tbb_2 (150 mg, 0.114 mmol, 1.00 equiv.) in benzene (5.0 mL) was treated with a stock solution (0.543 M in *n*-hexane) of tosyl azide (0.52 mL, 0.285 mmol, 2.50 equiv.) at room temperature and stirred for 14 h. Over time, the suspension first turned in to a red solution within 5 minutes and then transformed into a yellow suspension overnight. Analysis of an aliquot of the reaction mixture by ^1H NMR spectroscopy confirmed the complete consumption of Bi_2Tbb_2 and selective formation of complex **4a**. The reaction mixture was worked-up by removing the solvent under reduced pressure, followed by washing the yellow crude product first with a mixture of *n*-hexane/benzene (4:1, *v/v*) (1 x 2.5 mL) and finally by *n*-hexane (2.5 mL) at room temperature. Drying the residue under reduced pressure gave complex **4a** as a yellow powder (119 mg, 62% yield).

Note: This reaction can also be performed using toluene as the solvent with the same outcome.

Compound **4a** is an air-sensitive yellow powder. It can be stored under argon atmosphere at –30 °C for several months. It is poor soluble in aliphatic solvents, moderately soluble in benzene, and very well soluble in THF at room temperature. A sample solution of **4a** in THF-d_8 does not show any appreciable decomposition even after 5 hours at 65 °C.

^{1}H NMR (500 MHz, THF-d_8): δ 7.52 (s, 4H, $C^{3,5}$-*H*, Tbb), 7.19 (d, $^3J_{H\text{-}H}$ = 8.2 Hz, 4H, Ts), 6.95 (d, $^3J_{H\text{-}H}$ = 8.0 Hz, 4H, Ts), 2.94 (s, 4H, Dsi-*H*, Tbb), 2.27 (s, 6H, C^4-*Me*, Ts), 1.40 (s, 18H, C^4-C*Me*$_3$, Tbb), 0.10 (s, 72H, Si*Me*$_3$, Tbb).

^{13}C{^{1}H} NMR (126 MHz, THF-d_8): δ 220.4, 153.8, 150.8, 142.6, 142.0, 131.1, 130.0, 128.1, 35.8, 31.8, 29.6, 21.4, 1.9.

Elemental analysis: Calcd. in % for $C_{62}H_{112}Bi_2N_2O_4S_2Si_8$ (1656.34 g/mol): C 44.96, H 6.82, N 1.69, S 3.87; found: C 44.64, H 6.67, N 1.75, S 3.93.

2.6. Synthesis of compound 5

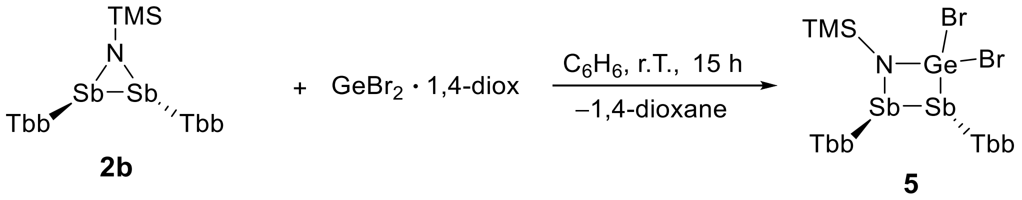

A colourless suspension of $GeBr_2$•1,4-dioxane (82 mg, 0.255 mmol, 1.25 equiv.) in benzene (5.0 mL) was treated with a yellow solution of complex **2b** (250 mg, 0.203 mmol, 1.00 equiv.) in benzene (5.0 mL) at room temperature and stirred for 15 h. Over time, the yellow suspension gradually changed into a yellow-orange solution. Analysis of an aliquot of the reaction mixture by ^{1}H NMR spectroscopy confirmed the complete consumption of complex **2b** and a selective formation of compound **5**. The reaction mixture was worked-up by evaporating the solvent under reduced pressure, followed by extracting the remaining yellow-orange crude product with *n*-pentane (20.0 mL) at room temperature. Volatiles were removed under reduced pressure and the yellow-orange residue was washed with a mixture of MeCN/Et_2O (4:1, *v/v*) (2 × 2.5 mL). Drying under reduced pressure gave compound **5** as a fine yellow powder (282 mg, 94% yield).

Compound **5** is an air-sensitive yellow amorphous solid. It can be stored under argon atmosphere at –30 °C for several months. It is moderately soluble in aliphatic solvents and Et_2O, very good soluble in THF and benzene but insoluble in MeCN at room temperature. A sample solution of **5** in C_6D_6 does not show any appreciable decomposition even after 4 hours at 85 °C.

^{1}H NMR (500 MHz, C_6D_6): δ 7.07 (s, 2H, $C^{3,5}$-*H*, Tbb), 7.07-6.93 (very br s, 2H, $C^{3,5}$-*H*, Tbb), 2.79 (br s, $\Delta\nu_{1/2}$ = 23 Hz, 2H, Dsi-*H*, Tbb), 2.58 (s, 2H, Dsi-*H*, Tbb), 1.32 (s, 9H, C^4-C*Me*$_3$, Tbb), 1.31 (s, 9H, C^4-C*Me*$_3$, Tbb), 0.36 (s, 18H, Si*Me*$_3$), 0.34 (s, 18H, Si*Me*$_3$), 0.29 (br s, 54H, Si*Me*$_3$), 0.28 (s, 9H, Si*Me*$_3$).

^{13}C{^{1}H} NMR (126 MHz, C_6D_6): δ 151.51, 151.47, 150.4, 149.6, 135.4, 123.8, 122.9, 36.3, 34.2, 34.2, 30.9, 30.8, 3.5, 1.8, 1.47, 1.42, 1.0.

Note: The **^{13}C{^{1}H}** NMR spectrum at 298 K of compound **5** shows 17 carbon signals instead of 29 according to the C_1–symmetric solid-state structure. This is due to overlapping of the signals and hindered rotation of the Tbb groups.

^{1}H NMR (500 MHz, THF-d_8): δ 6.94 (s, 2H), 6.92 (br s, 2H), 2.60 (br s, $\Delta\nu_{1/2}$ = 21 Hz, 2H), 2.36 (s, 2H), 1.33 (s, 9H), 1.32 (s, 9H), 0.23 (s, 18H), 0.24-0.21 (br s, 54H), 0.15 (s, 18H), 0.05 (s, 9H).

^{1}H NMR (300 MHz, THF-d_8, 193 K): δ 6.98 (d, $^4J_{H-H}$ = 2 Hz, 1H), 6.93 (d, $^4J_{H-H}$ = 2 Hz, 1H), 6.85 (d, $^4J_{H-H}$ = 2 Hz, 1H), 6.77 (d, $^4J_{H-H}$ = 2 Hz, 1H), 2.71 (s, 1H), 2.44 (s, 1H), 2.35 (s, 1H), 2.30 (s, 1H), 1.33 (s, 9H), 1.32 (s, 9H), 0.38 (s, 9H), 0.29 (s, 9H), 0.27 (s, 9H), 0.22 (s, 9H), 0.15 (s, 9H), 0.14 (s, 9H), 0.06 (s, 9H), 0.04 (s, 18H).

^{13}C{^{1}H} NMR (75 MHz, THF-d_8, 193 K): δ 152.3, 152.2, 152.0, 151.0, 150.2, 150.1, 144.2, 135.2, 124.8, 124.0, 123.8, 123.5, 37.5, 35.7, 35.3, 35.1, 34.1, 32.6, 31.5, 3.7, 3.2, 2.5, 2.4, 2.0, 1.7, 1.3, 0.9.

Note: The **^{13}C{^{1}H}** NMR spectrum at 193 K of compound **5** shows 27 carbon signals instead of 29 according to the C_1−symmetric solid-state structure. This is due to overlapping of the signals corresponding to Si*Me*$_3$ and C*Me*$_3$ carbons from the Tbb groups.

Elemental analysis: Calcd. in % for $C_{51}H_{107}Br_2GeNSb_2Si_9$ (1463.11 gm/mol): C 41.87, H 7.37, N 0.96; $C_{51}H_{107}Br_2GeNSb_2Si_9$•0.5 Et_2O (1500.17 g/mol): C 42.43, H 7.52, N 0.93; found: C 42.63, H 7.57, N 0.98.

2.7. Synthesis of compound 6

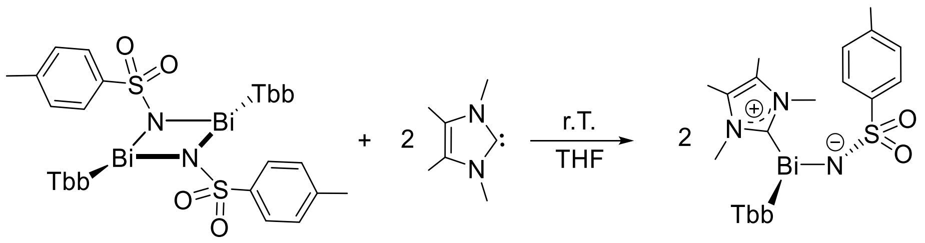

4a **6**

A mixture of solids **4a** (100 mg, 0.06 mmol, 1.00 equiv.) and IMe_4 (16 mg, 0.126 mmol, 2.1 equiv.) was treated with THF (3.0 mL) at room temperature and stirred for 5 minutes. An aliquot of the resulting pale-yellow solution was analysed by ^{1}H NMR spectroscopy, which revealed the complete consumption of complex **4a** and a very selective formation of compound **6**. The reaction mixture was worked-up by evaporating the solvent to dryness under reduced pressure, followed by a washing of the remaining yellow crude product with precooled (−30 °C) THF/*n*-pentane mixture (1:9, *v/v*) (3.0 mL). Drying the residue under reduced pressure gave compound **6** as beige powder (90 mg, 78% yield).

Compound **6** is an extremely air-sensitive, amorphous solid. It can be stored under argon atmosphere at –30 °C for several weeks. It is poorly soluble in *n*-pentane and Et_2O, moderately soluble in benzene but very well soluble in THF at room temperature. A solid sample of **6**, when kept at room temperature and exposed to sunlight for 2 hours, does not show any sign of decomposition. However, a sample solution of **6** in THF-d_8 starts to deteriorate unselectively at room temperature overnight.

^{1}H NMR (500 MHz, THF-d_8,): δ 7.49 (d, $^3J_{H-H}$ = 7.8 Hz, 2H, Ts), 6.99 (s, 2H, $C^{3,5}$-*H*, Tbb), 6.93 (d, $^3J_{H-H}$ = 7.9 Hz, 2H, Ts), 3.66 (s, 6H, N-Me_2, IMe_4), 3.04 (s, 2H, Dsi-*H*, Tbb), 2.27 (s, 3H, C^4-*Me*, Ts), 2.10 (s, 6H, $C^{4,5}$-Me_2, IMe_4), 1.26 (s, 9H, C^4-CMe_3, Tbb), 0.08 (br s, $\Delta\nu_{1/2}$ = 53.2 Hz, 18H, SiMe_3, Tbb), –0.11 (br s, $\Delta\nu_{1/2}$ = 45.7 Hz, 18H, SiMe_3, Tbb).

^{13}C{^{1}H} NMR (126 MHz, THF-d_8): δ 176.8, 163.3, 152.8, 150.4, 150.2, 137.4, 128.5, 128.1, 126.5, 126.4, 36.4, 35.1, 31.6, 30.1, 21.4, 8.7, 1.3, 0.9.

^{1}H NMR (500 MHz, THF-d_8, 243 K): δ 7.42 (d, $^3J_{H-H}$ = 8 Hz, 2H), 6.94-6.93 (s + d, 4H), 3.04 (br. s,$\Delta\nu_{1/2}$ = 38 Hz, 2H), 2.26 (s, 3H), 2.07 (br s, $\Delta\nu_{1/2}$ = 12 Hz, 6H), 1.73 (s, 9H), 1.25 (s, 9H), 0.09 (s, 18H), –0.13 (s, 18H).

^{13}C{^{1}H} NMR (126 MHz, THF-d_8, 243 K): δ 175.9, 162.3, 152.5, 150.0, 149.5, 137.2, 128.6, 128.3, 126.2, 126.1, 36.2, 35.1, 31.7, 29.6, 21.4, 8.7, 1.2, 0.7.

Elemental analysis: Calcd. in % for $C_{38}H_{68}BiN_3O_2SSi_4$ (951.39 g/mol): C 47.92, H 7.20, N 4.41, S 3.37; found: C 48.17, H 7.15, N 4.43, S 3.42.

Note: The signals at 176.8 and 163.3 ppm in the **^{13}C{^{1}H}** NMR spectrum at 298 K are extremely low in intensity due to hindered rotation of the Tbb and the IMe_4 groups. The signal for the methyl groups on the nitrogen atoms from the IMe_4 in the **^{1}H** NMR spectrum at 243 K is overlapping with the NMR solvent peak at 3.58 ppm.

3. UV/Vis Spectroscopic Studies

All UV/vis measurements were conducted according to the standard operating procedure reported below.

All stock solutions were prepared in an Argon filled glovebox and immediately diluted and placed in a cuvette sealed with a J. Young valve. UV/Vis-measurements were carried out in THF at room temperature. All spectra were recorded in a range from 800-300 nm in data intervals of 1 nm. The UV/Vis bandwidth was set to 1.0 nm.

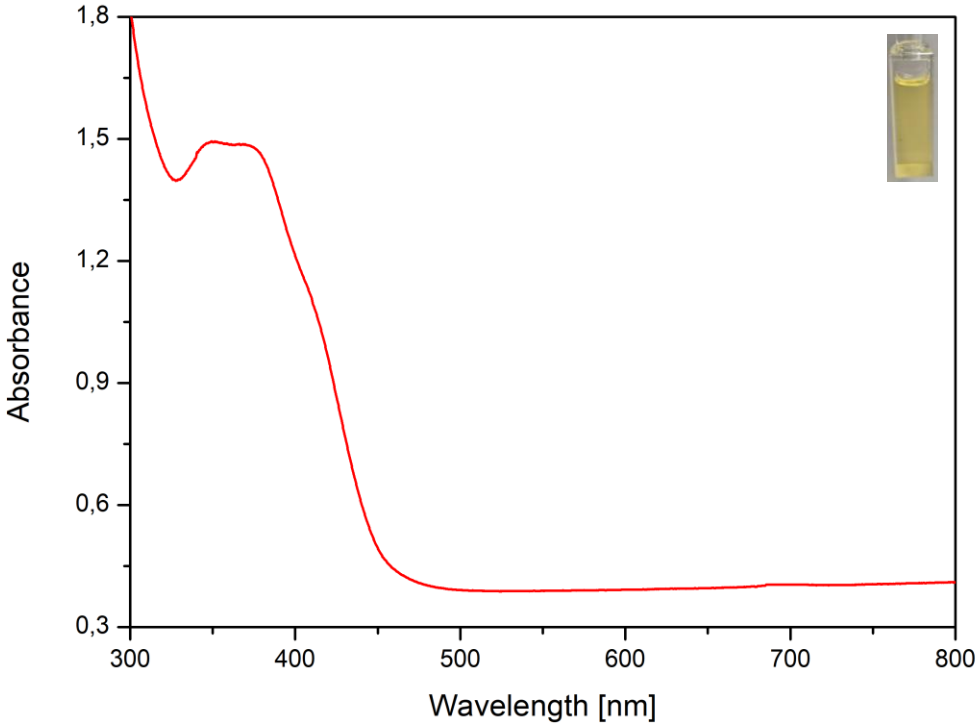

Figure S1. UV/Vis spectra of complex **2a** at a concentration of *c* = 100 μM in THF at room temperature.

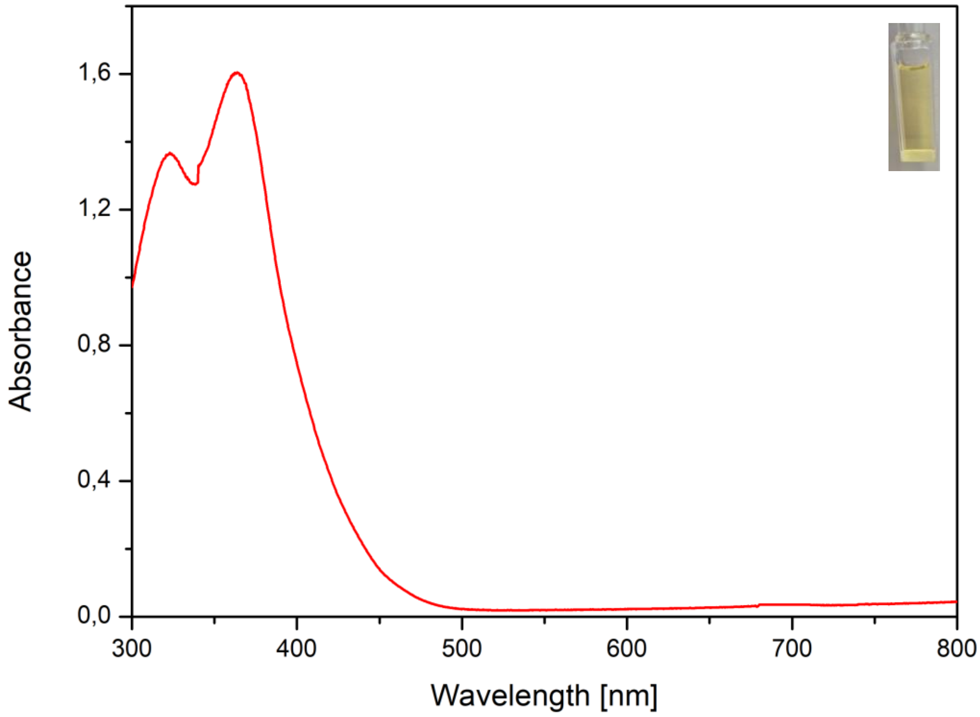

Figure S2. UV/Vis spectra of complex **2b** at a concentration of *c* = 100 µM in THF at room temperature.

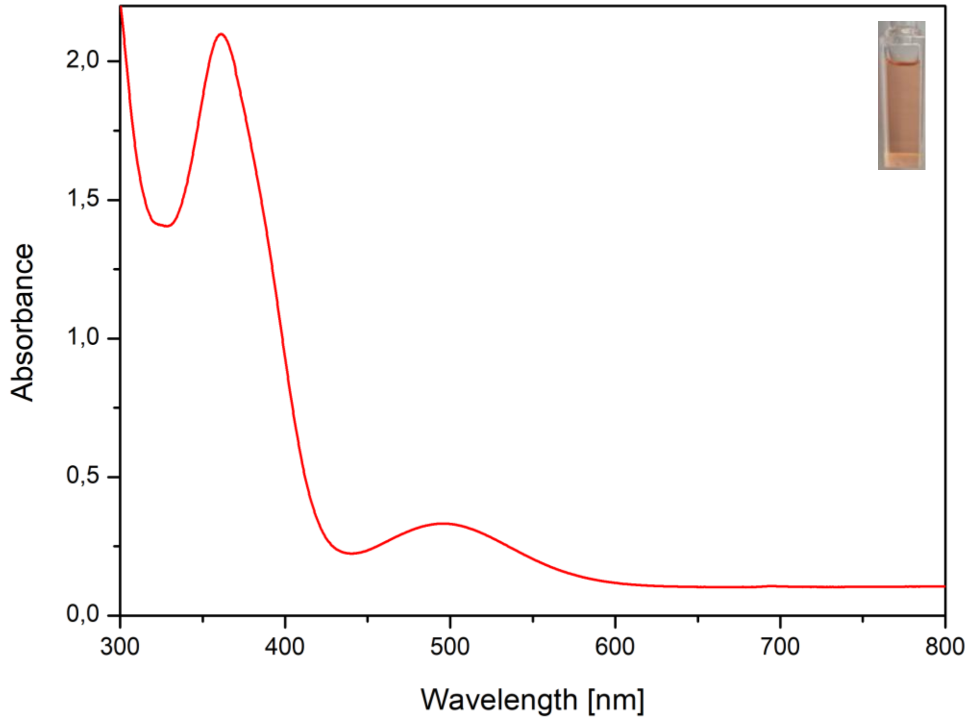

Figure S3. UV/Vis spectra of complex **2c** at a concentration of *c* = 173 µM in THF at room temperature.

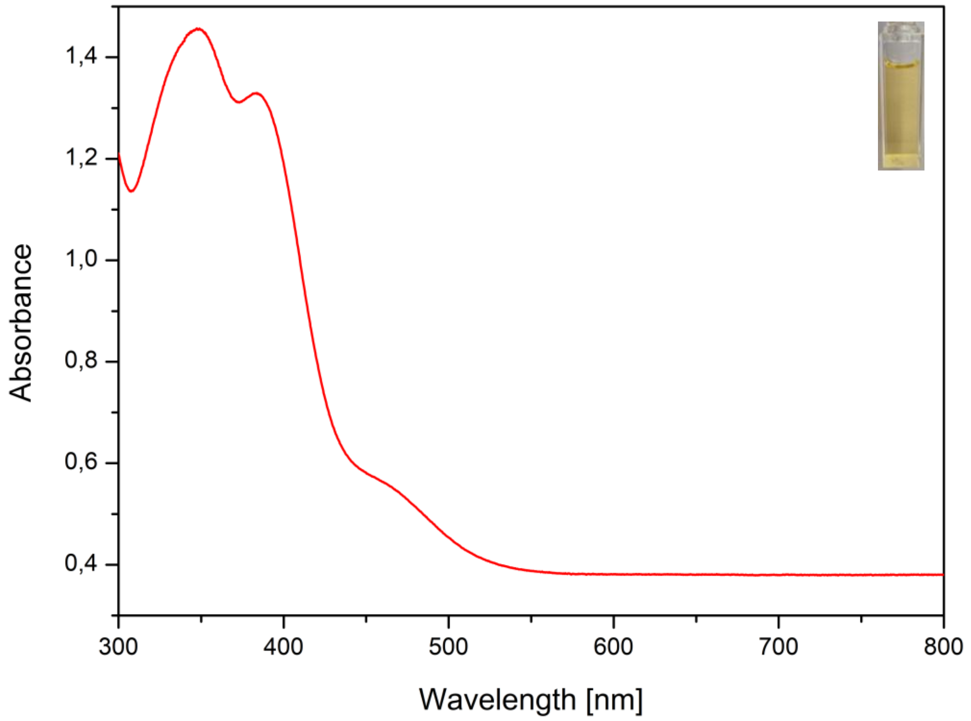

Figure S4. UV/Vis spectra of complex **2d** at a concentration of *c* = 100 μM in THF at room temperature.

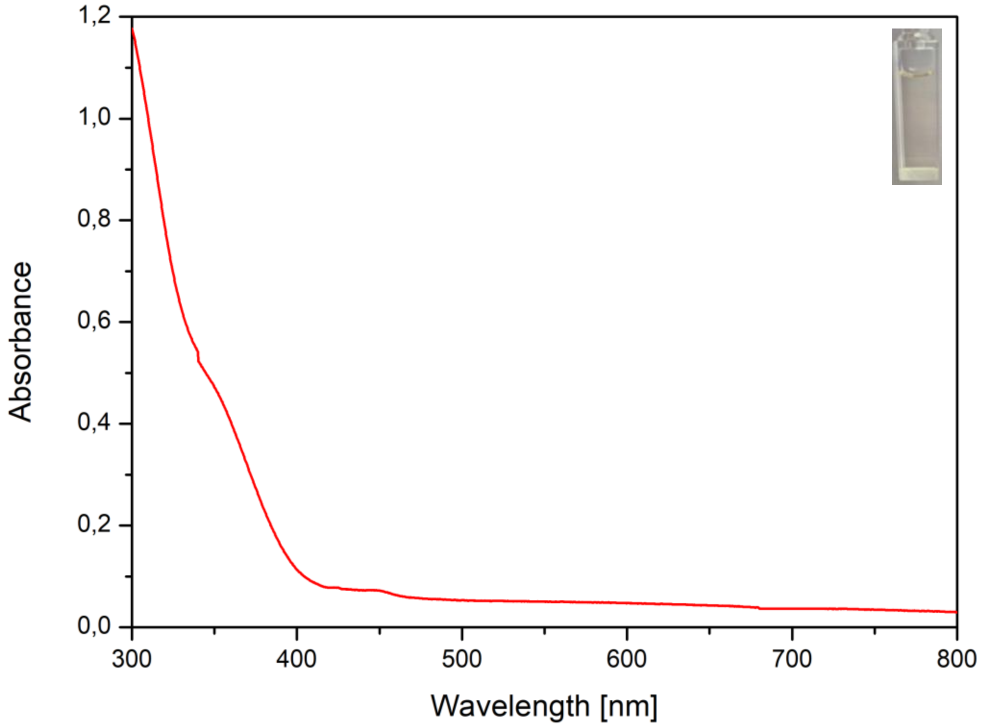

Figure S5. UV/Vis spectra of complex **4a** at a concentration of *c* = 100 μM in THF at room temperature.

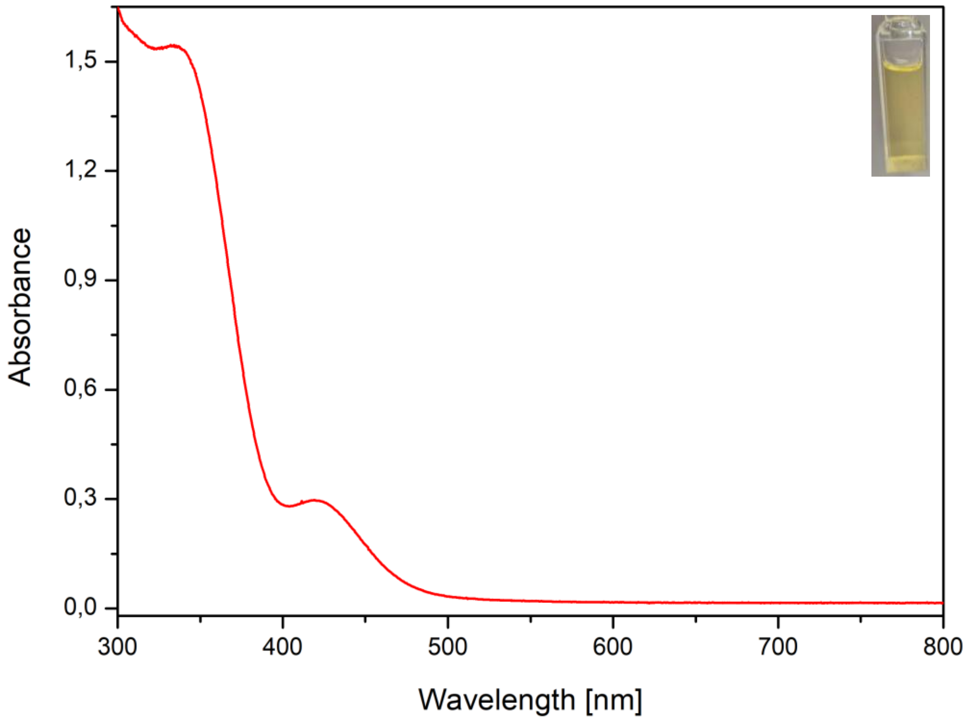

***Figure S6*.** UV/Vis spectra of compound **5** at a concentration of *c* = 100 μM in THF at room temperature.

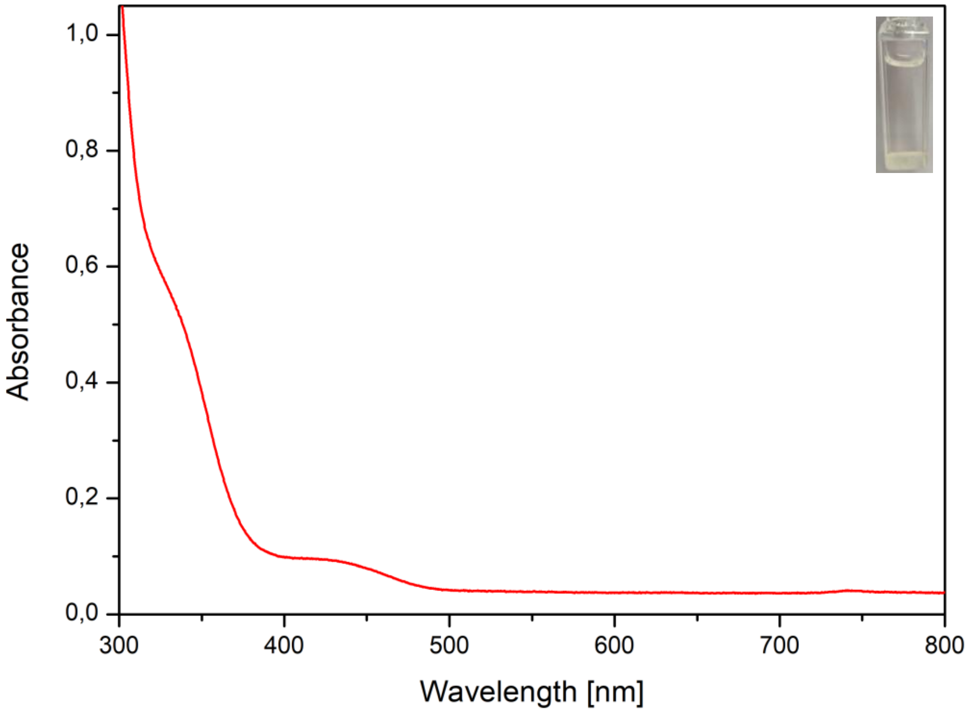

***Figure S7*.** UV/Vis spectra of compound **6** at a concentration of *c* = 166 μM in THF at room temperature.

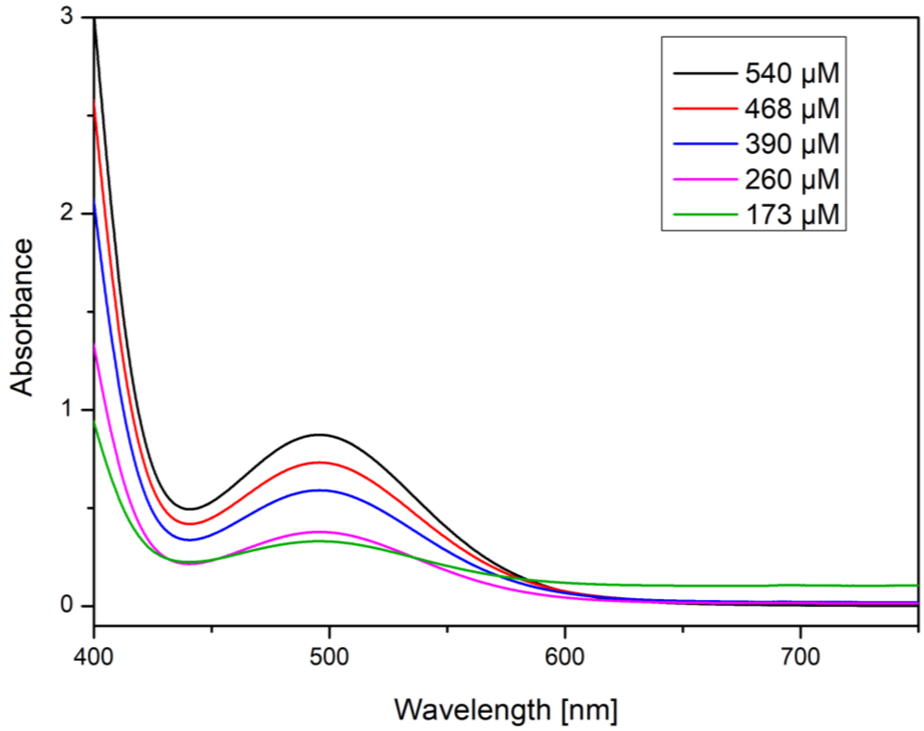

Figure S8. UV/Vis spectra of complex **2c** at different concentration in THF at room temperature.

Molar absorption coefficient ε was obtained by plotting the absorbance A against sample concentration c as the slope k of the linear regression analysis as in:

$$A = k \cdot c + b$$

Where b denotes the y-axis intercept. An overview of calculated ε-values is given in the table below.

Table S1. Calculated molar absorption coefficient for complex **2c**.

**Complex**	**ε ($M^{-1}\cdot cm^{-1}$)**
**2c**	1518

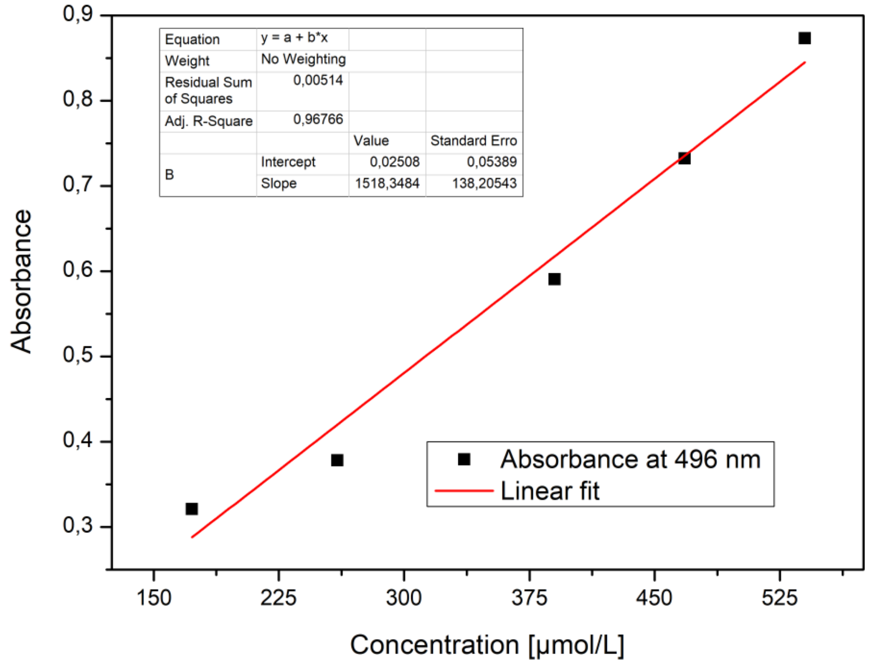

Figure S9. Absorbance of **2c** in THF (496 nm) plotted against respective sample concentration.

4. Computational methods

Theoretical analysis was conducted using the ORCA 6.0.1 software.[7] Initial molecular structures were obtained from XRD where possible or build with the free software Avogadro[8] before optimisation on the meta-GGA TPSS[9] in combination with the dispersion correction term D4[10–13] and applying the def2-TZVP[14] basis set. For cost efficient calculations the RI approximation[15] was applied using automatic selected auxiliary basis sets.[16] The optimised structures are characterised by frequency analysis (no imaginary frequency) and with thermal corrections (at 298.15 K and 1 atm) according to the modified ideal gas-rigid rotor-harmonic oscillator model. Single point energies were computed using the double hybrid PWPB95[17] using the 'exact two component' (X2C) Hamiltonian[18] module implemented in ORCA in combination with the dispersion correction term D4,[10] as a basis set x2c-QZVPPall was chosen.[19] For cost efficient calculations the RI approximation[15,20] was applied using automatic selected auxiliary basis sets.[16] The X2C treatment and the x2c-QZVPPall basis set were chosen to address the relativistic effects for all electrons. Localised orbitals, e.g., intrinsic bond orbitals (IBO),[21] were also computed on the same level. In the case of Bi containing compounds, localised orbitals were computed using the Pipek-Mezey localisation method[22] due to software limitations. Mulliken orbitals composition values were directly taken from these localisation output. Hybridisation values were obtained from Löwdin orbital analysis of the localised orbitals. Ring strain energies were computed on the TPSS-D4/def2-TZVP level of theory and corrected by their zero-point energy (ZPE). This allows direct comparison to the 1,2-distibirane-3-ylidene reported previously.[23]

Quantum theory of molecules in atoms (QTAIM)[24] analysis was performed using the software MultiWFN 3.8.[25,26] Critical points (CP) were searched until the Poincare-Hopf relationship was satisfied and all given values were taken directly from the MultiWFN output. The natural atomic charges (NPA) and the Natural Resonance Theory (NRT) were computed using the NBO 7.0 program.[27] The chemical bonding analysis has been also performed by combining the Extended Transition State (ETS)[28] method with the Natural Orbitals for Chemical Valence (NOCV) theory,[29] that is the ETS-NOCV method introduced by Ziegler et al.[30] Both NBO 7.0 and ETS-NOCV calculations were performed at the x2c-PW6B95-D4/x2c-QZVPPall/TPSS-D4/def2-TZVP level of theory. Mayer bond indices[31] were computed using Orca 6.0.1 and delocalisation indices using MultiWFN at the same level of theory. TD-DFT calculations were performed at the TPSS-D4-SOC-CPCM(THF)[32]/ZORA-TZVPP level of theory using 30 excited states using ORCA 6.0.1 program.

Structures and orbitals were visualised with USCF Chimera.[33] Orbital surfaces were plotted with an isosurface value of 0.04 a.u.

4.1. Additional material: Azadistibiridines 2a, 2b, 2c and 2d

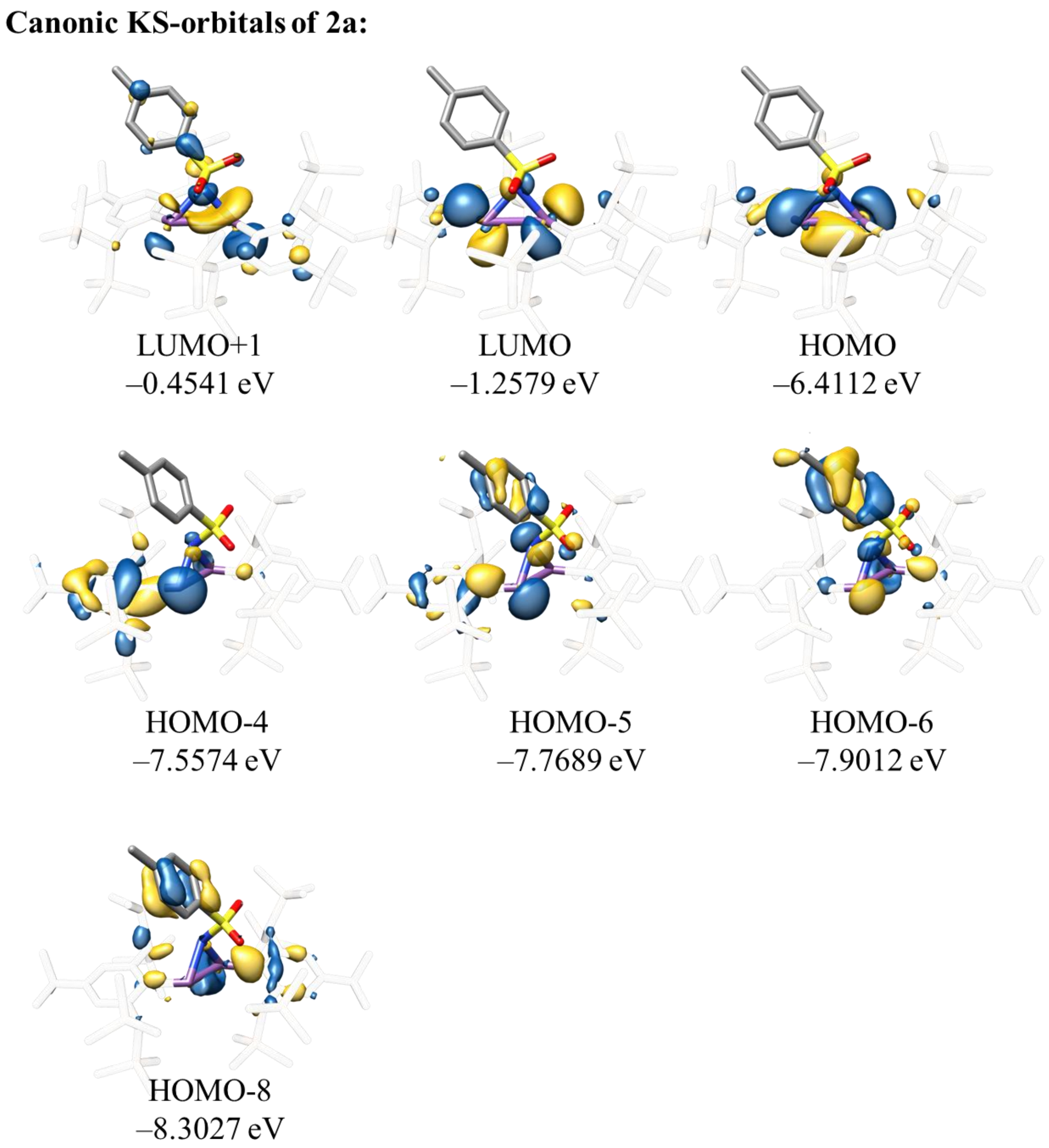

Figure S10. Selection of the frontier molecular orbitals of **2a** together with their absolute orbital energies (in eV) obtained from PWPB95/x2c-QZVPPall DFT calculations. Frontier molecular orbitals which showed negligible contribution on the central azadistibiridine motif have been omitted.

Selected localized orbitals:

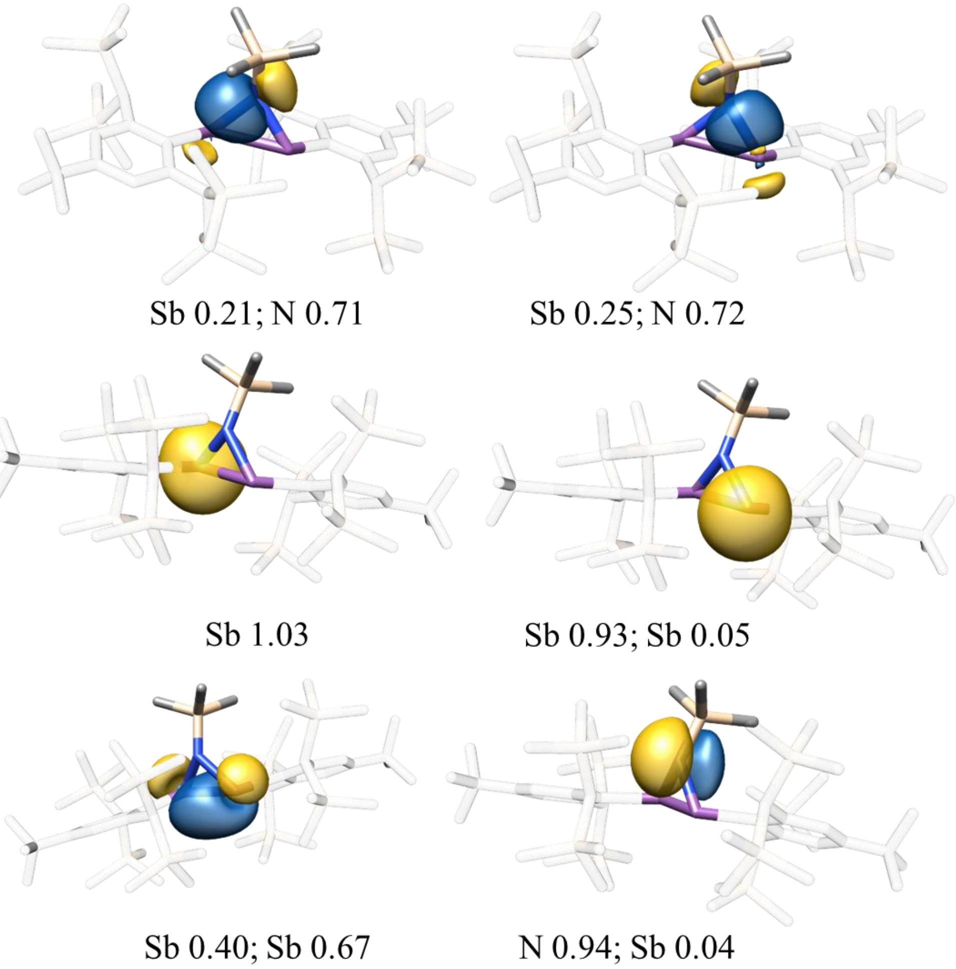

QTAIM:

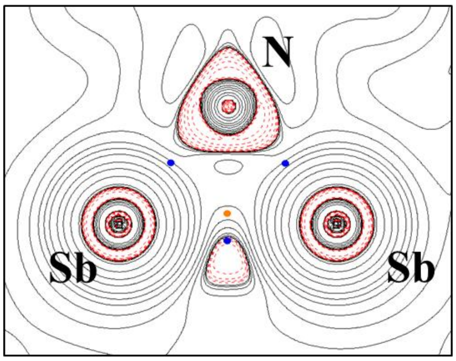

Figure S11. Selected localised orbitals with Mulliken composition analysis values and 2D plot Laplacian of the electron density in the Sb, Sb, N plane of **2b**. Bond critical points in blue, ring critical points in orange.

Selected localized orbitals:

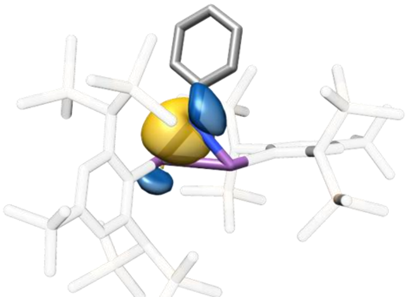

N 0.74; Sb 0.25

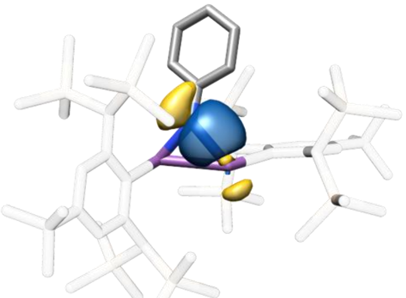

N 0.74; Sb 0.23

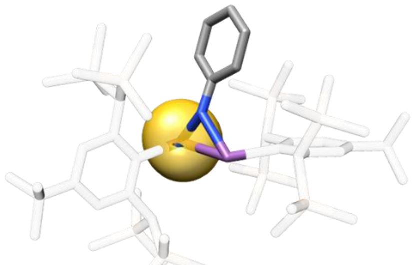

Sb 0.95

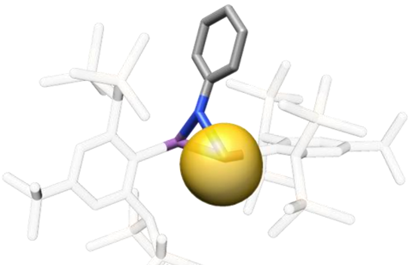

Sb 1.07

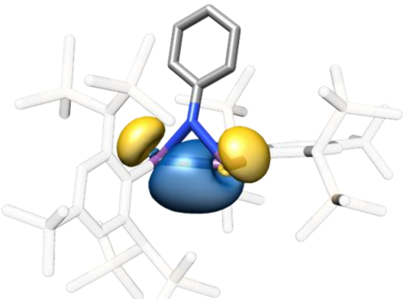

Sb 0.37; Sb 0.65

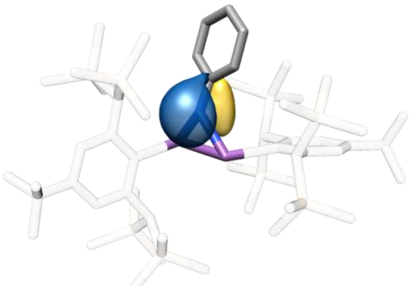

N 0.87; Sb 0.07

QTAIM:

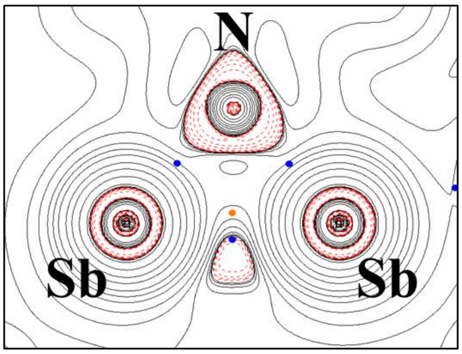

Figure S12. Selected localised orbitals with Mulliken composition analysis values and 2D plot Laplacian of the electron density in the Sb, Sb, N plane of **2c**. Bond critical points in blue, ring critical points in orange.

Selected localized orbitals:

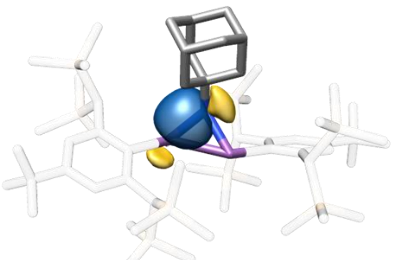

N 0.70; Sb 0.30

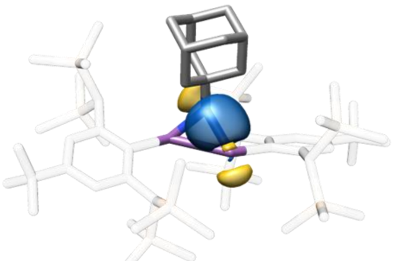

N 0.70; Sb 0.25

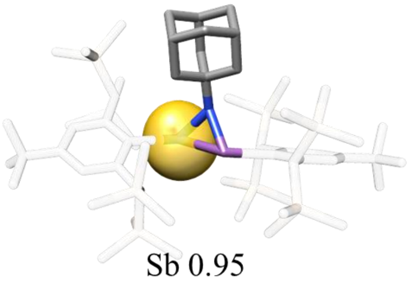

Sb 0.95

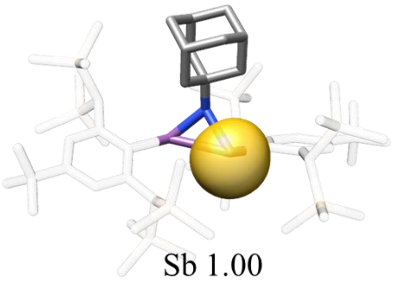

Sb 1.00

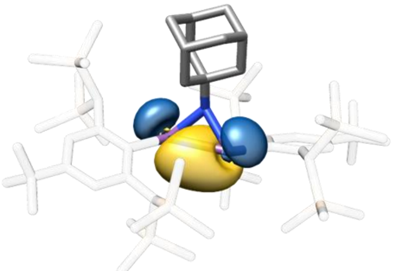

Sb 0.53; Sb 055

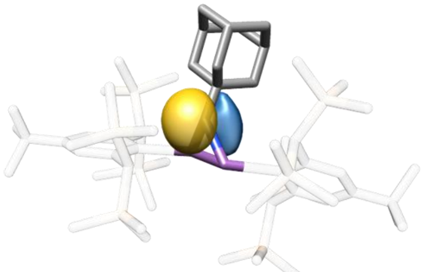

N 0.85; Sb 0.09

QTAIM:

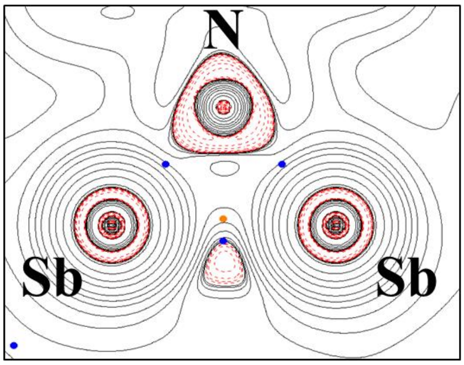

Figure S13. Selected localised orbitals with Mulliken composition analysis values and 2D plot Laplacian of the electron density in the Sb, Sb, N plane of **2d**. Bond critical points in blue, ring critical points in orange.

4.2. Additional material: Canonical Orbitals of compound 5

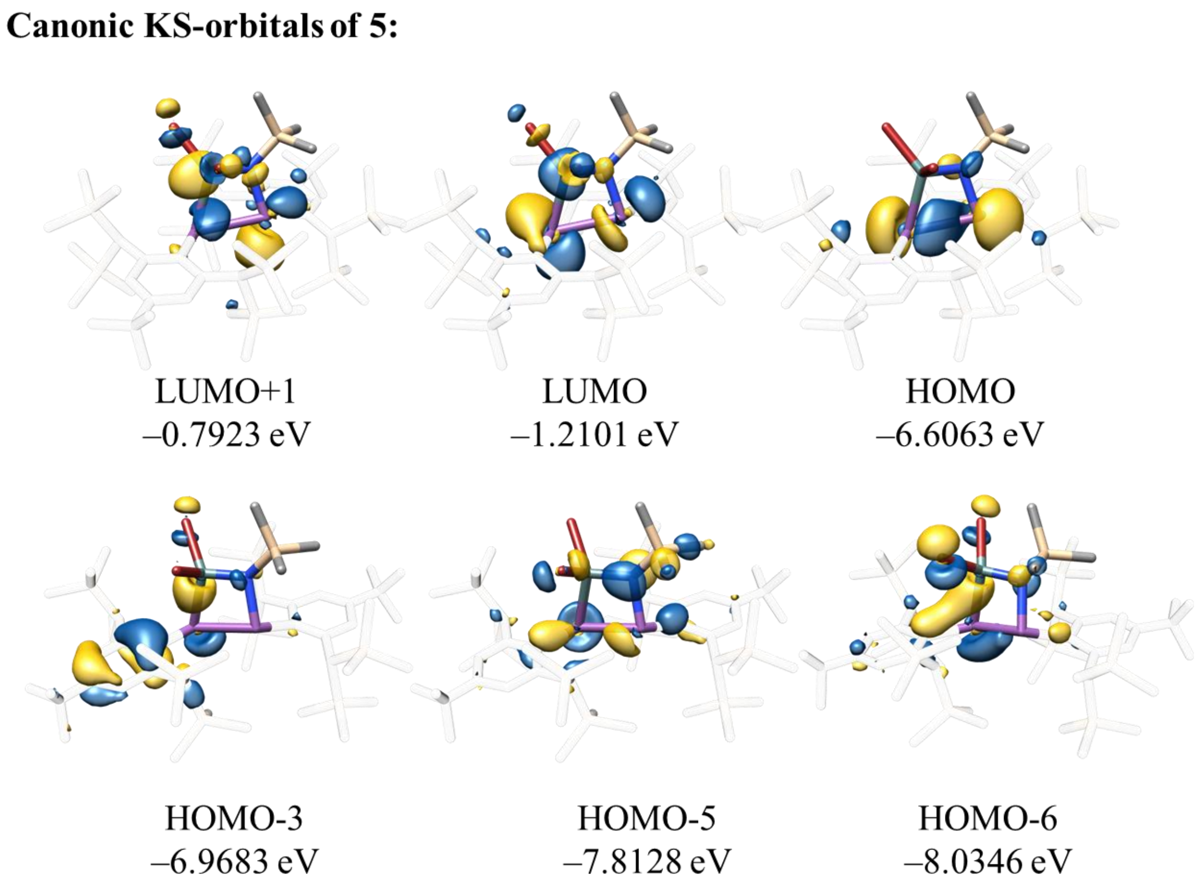

Figure S14. Broader selection of the frontier molecular orbitals of compound **5** together with their absolute orbital energies (in eV) obtained from PWPB95/x2c-QZVPPall DFT calculations. Frontier molecular orbitals which showed negligible contribution on the central 1,2,3,4-azadistibagermetidine (**5**) motif have been omitted.

4.3. Additional material: Outwards bond bending in selected three-membered rings

We have recognised the potential of the angle between two bonding atoms and its bond critical point (BCP) for comparing the degree of 'banana bonding'. Since the investigated azadistibiridines **2a-d** exhibit significant outwards bond bending of around 163°, mentioned in the main body of the publication, we investigated some selected simple three membered rings for a direct comparison. The selected rings are presented below:

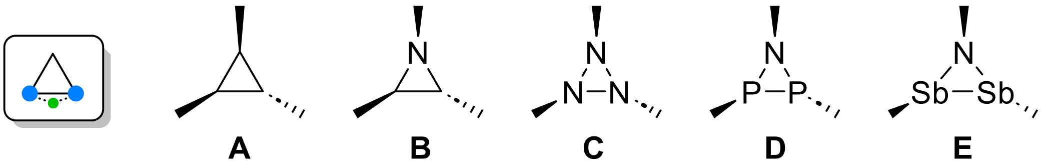

Figure S15. Selected three membered rings for comparison of the degree of outwards bond bending by the Atom-BCP-Atom angle. Considered atoms in the angle are at the blue circled position and their BCP is marked in green.

Table S2. Atom-BCP-Atom angle in ° in three membered rings **A-E**.

**Three membered ring**	**Atom-BCP-Atom angle in °**
trans-1,2,3-trimethylcyclopropane **A**	173.0
trans-1,2,3-trimethylaziridine **B**	170.5
1,2,3-trimethyltriaziridine **C**	174.5
trans-1,2,3-trimethylazadiphosphiridine **D**	168.4
trans-1,2,3-trimethylazadistibiridine **E**	167.8

The model azadistibiridine systems **E** indeed possess the smallest atom-BCP-atom angle, *i.e.* Sb-BCP-Sb, of the investigated systems clearly showing significant bond bending, even larger than that of cyclopropane **A**, which is a commonly used example for banana bonding in small ring systems. Remarkably, the azadiphosphiridine's (**D**) angle is very close to that of **E**. In the real systems **2a-d** the steric bulk seems to favor the bond bending even more. An effect from the N-substituent seems to be negligible as almost no variation for the angle in **2a-d** is observed.

5. Mechanistic study, ETS-NOCV and TD-DFT results

5.1. Mechanistic studies

We have investigated the mechanism of formation of **2a** and **4a**, as well as the hypothetical **Bi-2a** and **Sb-4a**. The results for Sb are presented in ***Figure S16***, while those for Bi are provided in ***Figure S17***. The energies were computed in toluene at three different levels of theory. Geometries were first optimised at the TPSS-D4/def2-TZVP level, which incorporates scalar relativistic effects via effective core potentials (ECPs). Subsequently, single-point energy calculations were performed using the meta-hybrid PW6B95 functional, which has been shown to provide comparable accuracy than the double-hybrid PWPB95 functional,[34] but with significantly lower computational cost, which is critical given the size of some intermediates (up to 176 atoms). To account for relativistic effects, we employed two different approaches: (i) ECP-based approach using the def2-TZVP basis set, which incorporates scalar relativistic corrections. (ii) Exact-two-component (x2c) approach using the fully uncontracted x2c-def2-TZVPPAll basis set, explicitly designed for relativistic calculations. The results from both approaches are identical (with energies shown in black and red), demonstrating that the def2-ECP auxiliary basis set with scalar relativistic corrections is a reliable and computationally efficient alternative for studying these systems.

Regarding the mechanism, the first step involves a [3+2] cycloaddition reaction between the azide and Sb_2Tbb_2 (**1**), leading to the formation of a transient intermediate (**INT1**, see ***Figure S16*)**, which is 21.3 kcal/mol more stable than the starting reactants. From this intermediate, two possible reaction pathways can be considered. The first pathway (route "a") involves the elimination of N_2, yielding **2a**, which is 47.0 kcal/mol more stable than **INT1**. This reaction is highly exothermic, consistent with experimental observation. The second pathway involves a second [3+2] cycloaddition with another molecule of azide, forming intermediate **INT2**, which is energetically unfavorable by 12.5 kcal/mol. **INT2** can then evolve into **INT3** through the elimination of two equivalents of N_2, either in a single step or in two consecutive steps, producing two equivalents of Ts–N=Sb–Tbb (**INT3**). This step is highly exothermic (–78.1 kcal/mol). A final [2+2] cycloaddition between two **INT3** units would lead to the formation of the hypothetical four-membered ring **Sb-4a**, which was not observed experimentally.

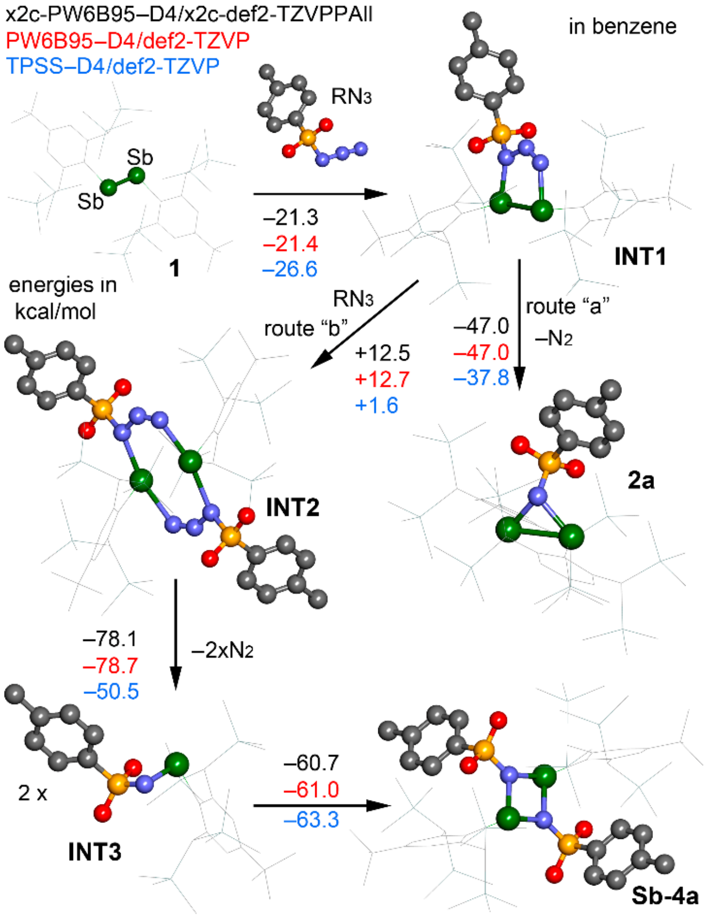

Figure S16. Proposed mechanistic route to **2a** and **Sb-4a**. The molecules are represented in ball and stick. The Tbb groups are represented as grey lines. Energies are in kcal/mol.

A similar analysis for Bi is provided in ***Figure S17***, where the pathway from Bi_2Tbb_2 (**3**) to hypothetical **Bi-2a** through the [3+2] cycloaddition with azide follows a nearly identical energy profile compared to Sb. The key difference appears in the second [3+2] cycloaddition reaction (route "b"), where the formation of **INT2** is only 6.9 kcal/mol less favorable using the PW6B95 functional, suggesting that this intermediate could form readily at room temperature. Furthermore, this second cycloaddition is energetically favorable for Bi using the TPSS-D4 functional, whereas it remains unfavorable for Sb. The subsequent elimination of two N_2 molecules leads to two equivalents of Ts–N=Bi–Tbb (**INT3**), a highly exothermic step. Finally, the [2+2] homo-dimerisation results in the formation of **4a**, which is 62.8 kcal/mol more stable than **INT3**. This mechanistic proposal highlights that the four-membered ring is thermodynamically more stable than the three-membered ring, regardless of the existence of **INT3** or the validity of the proposed mechanism. Most notably, the second [3+2] cycloaddition with azide is significantly more favorable for Bi than Sb (by 5.7 kcal/mol) across all three levels of theory. This provides a plausible explanation for why the four-membered ring is observed exclusively for Bi. However, the fact that there are not significant differences regarding the

formation of **2a** versus **Bi-2a** and **4a** versus **Sb-4a** (similar stabilisation), strongly suggest that kinetic effects are determinant to explain the different behavior of Sb and Bi.

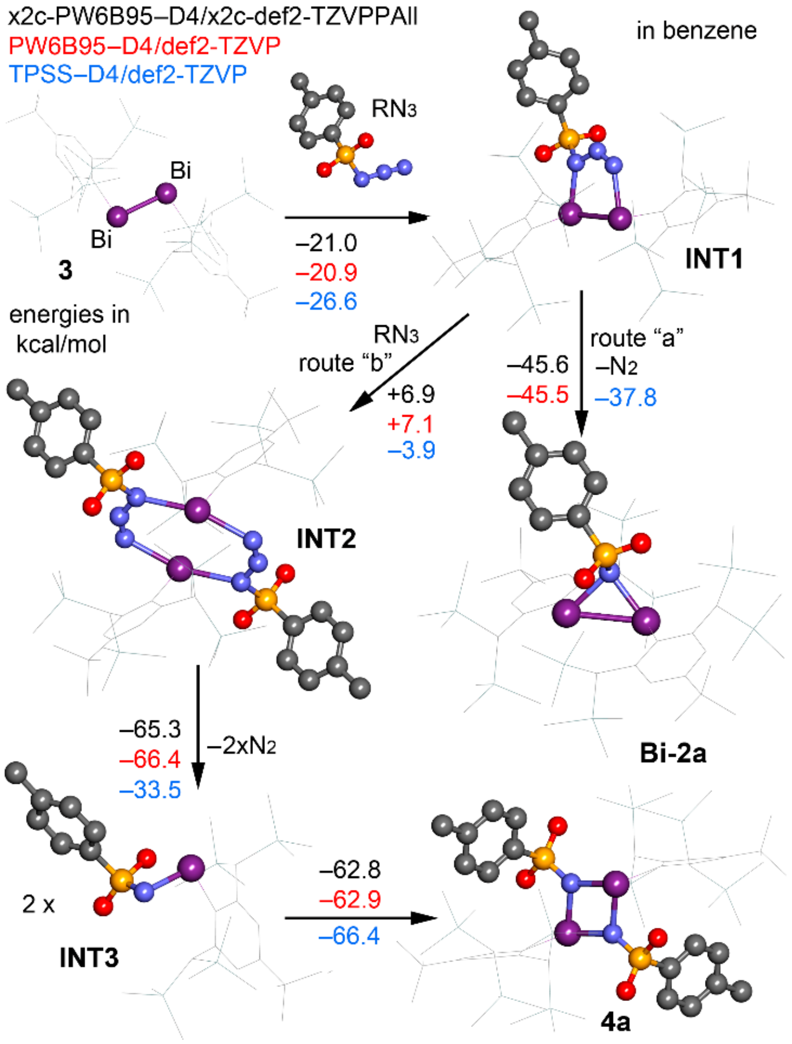

Figure S17. Proposed mechanistic route to **Bi-2a** and **4a**. The molecules are represented in ball and stick. The Tbb groups are represented as grey lines. Energies are in kcal/mol.

5.2. Canonical and localised molecular orbitals of compound 6

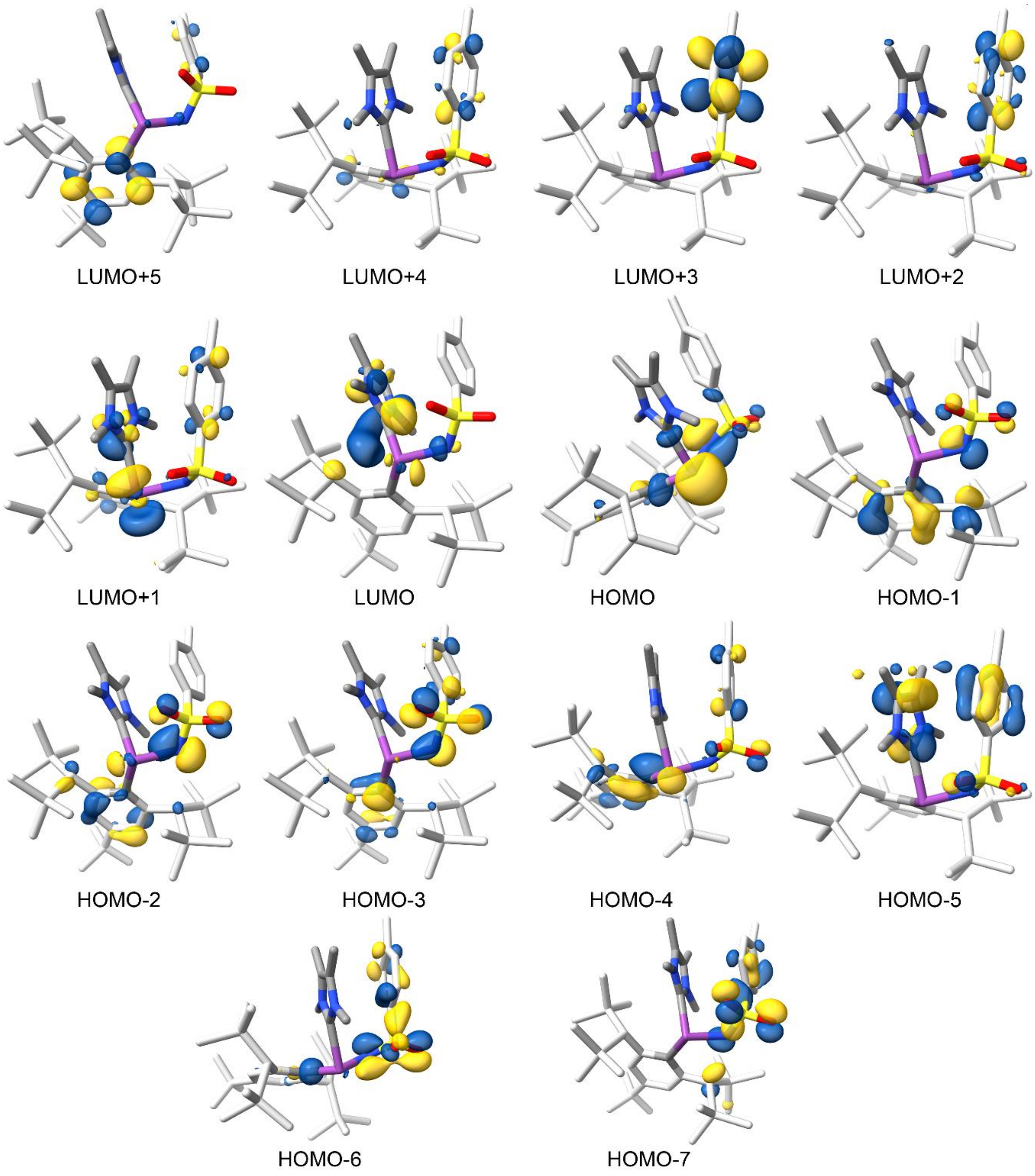

Figure S18. Canonical orbitals of compound **6** from HOMO-7 to LUMO+5.

Selected localized orbitals

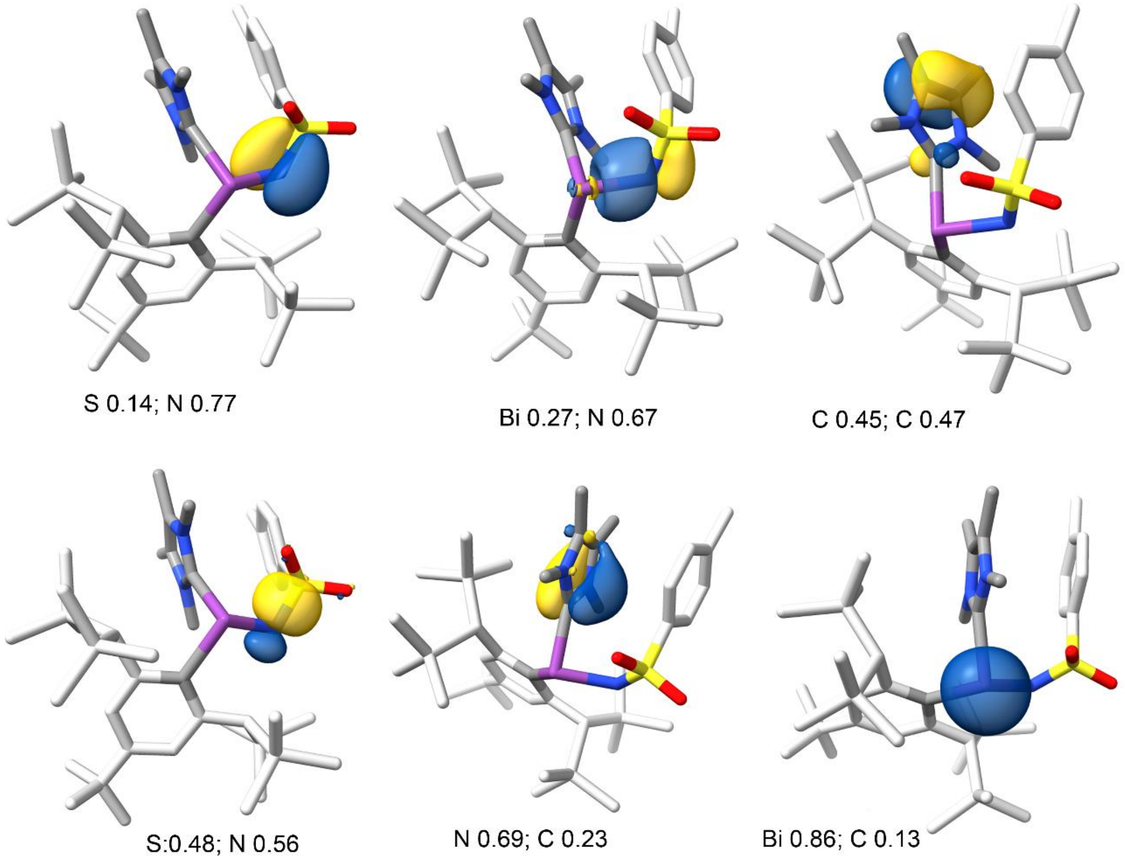

Figure S19. Selected localised orbitals of compound **6** with Mulliken composition analysis values.

5.3. ETS-NOCV analysis

The interaction between the Tbb–Bi=N–Ts fragment and the NHC unit in compound **6** was further investigated using the Energy Decomposition Analysis–Natural Orbitals for Chemical Valence (ETS-NOCV) method. This approach provides valuable insight into electron density redistribution during bond formation by identifying the principal channels of electron flow and quantifying the extent of charge transfer through the corresponding eigenvectors and eigenvalues of the deformation density matrix. The results reveal a dominating pair with unidirectional electron donation from the NHC to the Bi atom (1.64 e, see ***Table S3***).

Table S3. ETS-NOCV analysis of **6** with indication of the most important pair. Electron transfer energies (eigenvalue) are in a.u. and pair energies in kcal/mol.

**Compound**	**Electron flow**	**Orbital composition**	**e transfer**	**Pair energy kcal/mol**	**Total pair energies kcal/mol**
**6**	NHC-->Bi	Donor: 62.2% s, 37.1% p, 0.7% d<br>Acceptor: 21.3% s, 77.0% p, 1.5% d	1.64	-72.1	-101.2

A total of 1,289 NOCV pairs and 2,758 orbitals were identified. This number reduces to 158 pairs when only those with a charge transfer greater than 0.001 e are considered. In ***Table S4***, we report the 15 pairs with interaction energies equal to or greater than 0.5 kcal/mol in absolute value. Among these, Pair 1, described in greater detail in ***Table S3*** and the main text, clearly dominates. The remaining pairs, while not negligible, each contribute less than 10% of the energy of Pair 1 and can be considered residual. Therefore, our bonding analysis between Bi and the NHC ligand in compound **6** focuses primarily on this dominant interaction.

Table S4. NOCV pairs with interaction energies equal to or greater than 0.5 kcal/mol in absolute value for compound **6.**

Pair	Energy of the Pair (kcal/mol)	Donor NOCV	Eigenvalue (a.u.)	NOCV Energy (eV)	Acceptor NOCV	Eigenvalue (a.u.)	NOCV Energy (eV)
1	–72.1	1	0.838	–4.770	2578	–0.838	–1.041
2	–7.3	2	0.270	–4.620	2577	–0.270	–3.447
3	–5.2	3	0.167	–2.079	2576	–0.167	–0.726
4	–2.3	4	0.118	–2.704	2575	–0.118	–1.848
5	–1.5	5	0.095	–1.425	2574	–0.095	–0.750
6	–1.1	6	0.080	–2.455	2573	–0.080	–1.841
7	–0.8	7	0.074	–2.401	2572	–0.074	–1.917
8	–0.7	8	0.066	–2.140	2571	–0.066	–1.658
9	–0.7	9	0.061	–1.954	2570	–0.061	–1.454
10	–0.6	10	0.058	–2.910	2569	–0.058	–2.425
11	–0.6	11	0.056	–2.930	2568	–0.056	–2.500
12	–0.6	12	0.052	–2.279	2567	–0.052	–1.809
13	–0.5	13	0.051	–1.876	2566	–0.051	–1.433
14	–0.5	14	0.048	–1.435	2565	–0.048	–1.007
15	–0.5	15	0.045	–2.607	2564	–0.045	–2.119

5.4. TD-DFT results

Table S5. Theoretical TD-DFT Absorption spectrum (λ and oscillator strength) via transition electric dipole moments (in a.u.) in THF for compounds **2a-d**, **4a**, **5** and **6**. The orbital composition and the main excited state corresponding to the band are indicated. For compounds **2a** and **2d**, values corresponding to the shoulder bands have also been included using SOC-TD-DFT calculations with the all-electron basis set (ZORA-def2-TZVP); these values are presented in italics.

**Compounds**	λ_{exp} (nm)	λ_{calc} (nm) and oscillator strength	Excitation	orbital (composition)
**2a**	348	343.7 (0.0395)	$S_0 \rightarrow S_9$	H-5→L (11.3%)<br>H-2→L+1 (53.9%)<br>H-1→L+1(25.6%)
	374	389.5 (0.0778)	$S_0 \rightarrow S_5$	H-4→L (64.0 %)
	413 shoulder	434.9 (0.0321)<br>*433.7*<br>*(0.0871)*	$S_0 \rightarrow S_1$<br>*$S_0 \rightarrow S_1$*	H-1→L (63.4 %)<br>H→L (36.6%)<br>*H-1→L (56.4 %)*<br>*H→L (40.6%)*
**2b**	323	317.6 (0.0275)	$S_0 \rightarrow S_{17}$	H-3→L+1 (81.3 %)<br>H→L+2 (11.7%)
	363	353.0 (0.0947)	$S_0 \rightarrow S_{10}$	H-7→L (13.1 %)<br>H-5→L+1 (70.6%)
**2c**	361	366.9 (0.0235)	$S_0 \rightarrow S_{10}$	H-2→L+1 (83.4 %)
	496	488.6 (0.0045)	$S_0 \rightarrow S_3$	H-2→L (100.0 %)
**2d**	347	353.8 (0.0530)	$S_0 \rightarrow S_8$	H-5→L (25.7%)<br>H-1→L (21.9%)<br>H-1→L+1(19.0%)
	384	378.5 (0.0581)	$S_0 \rightarrow S_6$	H-5→L (33.9 %)<br>H →L+1 (60.9 %)
	468 shoulder	483.5 (0.0129)<br>*481.3 (0.0195)*	$S_0 \rightarrow S_1$<br>*$S_0 \rightarrow S_1$*	H-1→L (23.5 %)<br>H →L (76.5 %)<br>*H-1→L (20.2 %)*<br>*H →L (78.4 %)*
**4a**	353 shoulder	330.2 (0.0397)	$S_0 \rightarrow S_{15}$	H-2→L+1 (66.4 %)<br>H-2 →L+2 (29.4%)
**5**	337	323.9 (0.0198)	$S_0 \rightarrow S_{15}$	H-5→L+1 (53.7%)<br>H→L+2(27.0%)
	425	422.3 (0.0289)	$S_0 \rightarrow S_5$	H-3→L (79.8%)<br>H→L+1(16.1%)
**6**	336 shoulder	342.6 (0.0169)	$S_0 \rightarrow S_2$	H-1→L (69.8%)<br>H→L (30.2%)

6. Single crystal X-ray diffraction analysis

6.1. General

Suitable single crystals of the compounds **2a**, **2b**, **2c**, **2d**, **4a**, **5** and **6** for X-ray diffraction studies were grown as follows: clear greenish yellow planks of **2a**, Clear yellow prisms of **2b**, clear red planks of **2c**, clear orange plate-shaped crystals of **2d•(Et_2O)**, and clear yellowish-orange block shaped crystals of **5•(Et_2O)$_{0.5}$** were obtained *via* slow evaporation of their saturated solutions in MeCN/Et_2O (v/v) mixture at −30 °C, respectively. Yellow block shaped crystals of **4a** were grown upon slow diffusion of *n*-hexane into its saturated solution in toluene at −30 °C and clear yellow plate shaped crystals of **6** were obtained upon slow evaporation of its saturated solution in THF/*n*-dodecane (v/v) mixture at room temperature.
The crystals were protected with Fomblin® Y inside the glove box before mounting on the goniometer.
The data collections of **2a**, **4a**, and **5** were performed on a STOE IPDS-2T 2-circle diffractometer equipped with a low-temperature device (100(2) K, Oxford Cryostream 700er series, Oxford Cryosystems) by using Mo-*K*α-radiation (λ = 0.71073 Å, ASTIX++-optics) and an image-plate (34 cm diameter) detector system. Intensities were measured by fine-slicing ω-scans and corrected for background, polarisation and Lorentz effects. A semi-empirical absorption correction by scaling of reflection intensities with a subsequent spherical absorption correction was performed with LANA.[35]
Data collections of **2b**, **2c**, **2d,** and **6** were performed on BRUKER D8-VENTURE diffractometer using Mo-K_α(λ = 0.71073 Å, mirror optics) using a Photon III CE14 detector. The diffractometer was equipped with low–temperature devices Cryostream 800 series (Oxford Cryosystems), 100 K. Intensities were measured by fine-slicing φ- and ω-scans and corrected for background, polarisation and Lorentz effects.
The structures were solved by intrinsic phasing methods and refined by the least-squares procedure implemented in the SHELX program suite.[36] Hydrogen atoms were placed in geometrically calculated positions and included in the refinement process using a riding model on the bound carbon atom. Absorption corrections were performed by the empirical method implemented in SADABS 2016/2.[37]OLEX^2 and Mercury were used for data finalisation and visualisation.[38,39]

Special treatments/procedures (side occupancies and masked solvent contributions) are given below.

CCDC numbers: CCDC- 2440170 (**2a**), CCDC- 2440171 (**2b**), CCDC- 2440172 (**2c**), CCDC-2440173 (**2d•(Et$_2$O)**), CCDC-2440174 (**4a**), CCDC-2440175 (**5•(Et$_2$O)$_{0.5}$**), and CCDC-2440176 (**6**) contain the supplementary crystallographic data for this paper, which can be obtained free of charge from the Cambridge Crystallographic Data Centre *via* http://www.ccdc.cam.ac.uk/data request/cif.

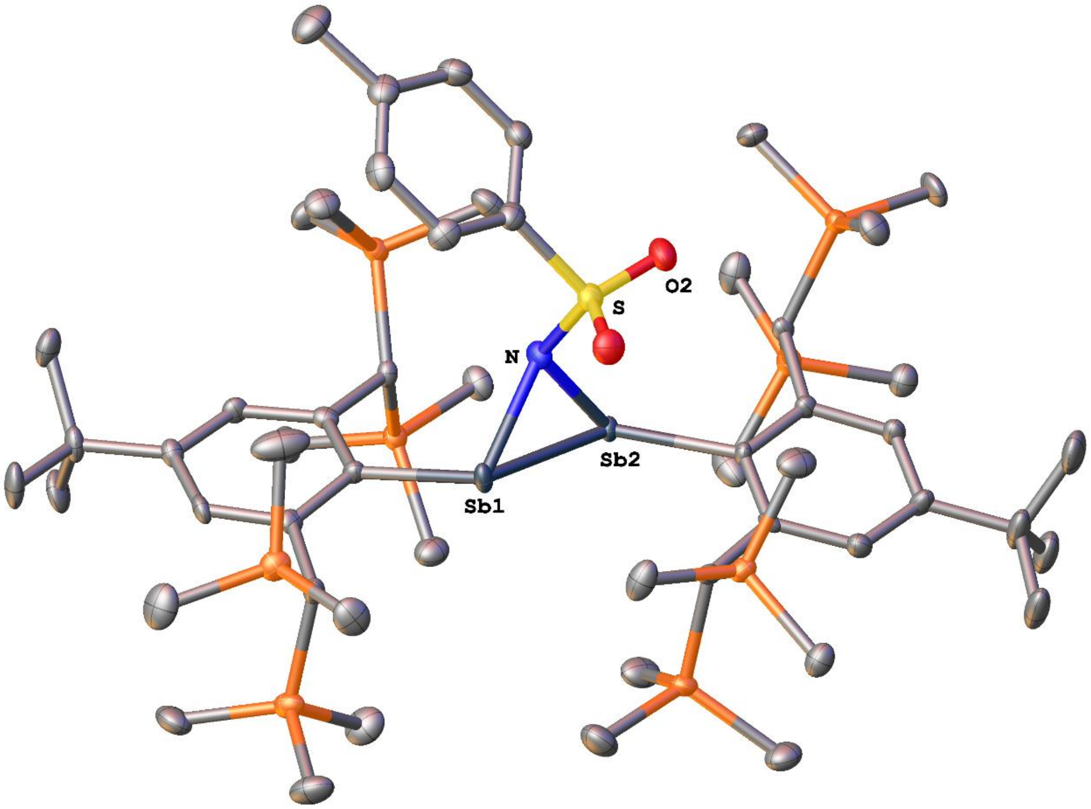

Figure S20. Solid state structure of **2a**. Hydrogen atoms are omitted for clarity. Thermal ellipsoids are set to 50% probability level.

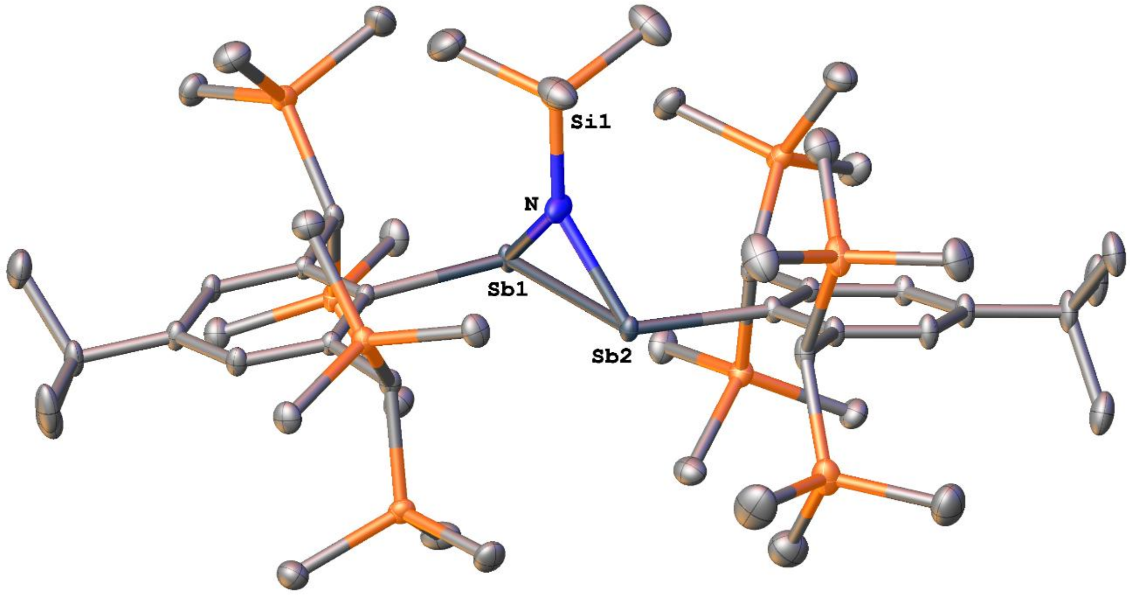

***Figure S21*.** Solid state structure of **2b**. Two side occupancies of the Sb_2-moiety (occupations of 3.3% and 1.7%) as well as hydrogen atoms are omitted for clarity. Thermal ellipsoids are set to 50% probability level.

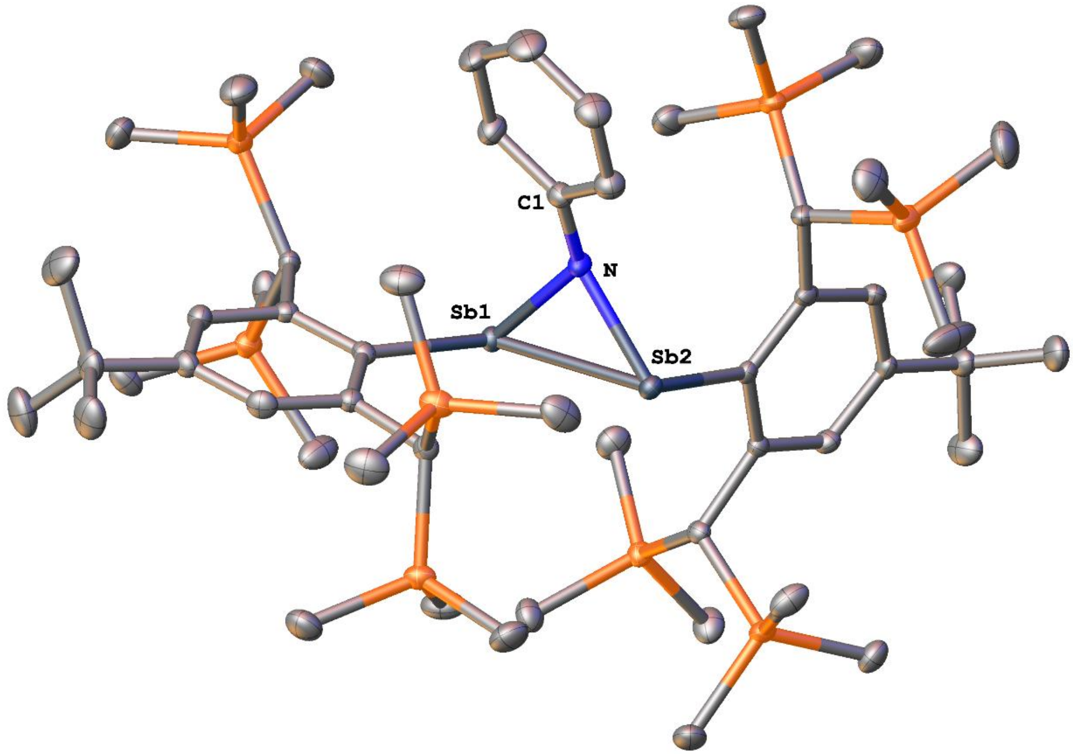

***Figure S22*.** Solid state structure of **2c**. Hydrogen atoms are omitted for clarity. Thermal ellipsoids are set to 50% probability level.

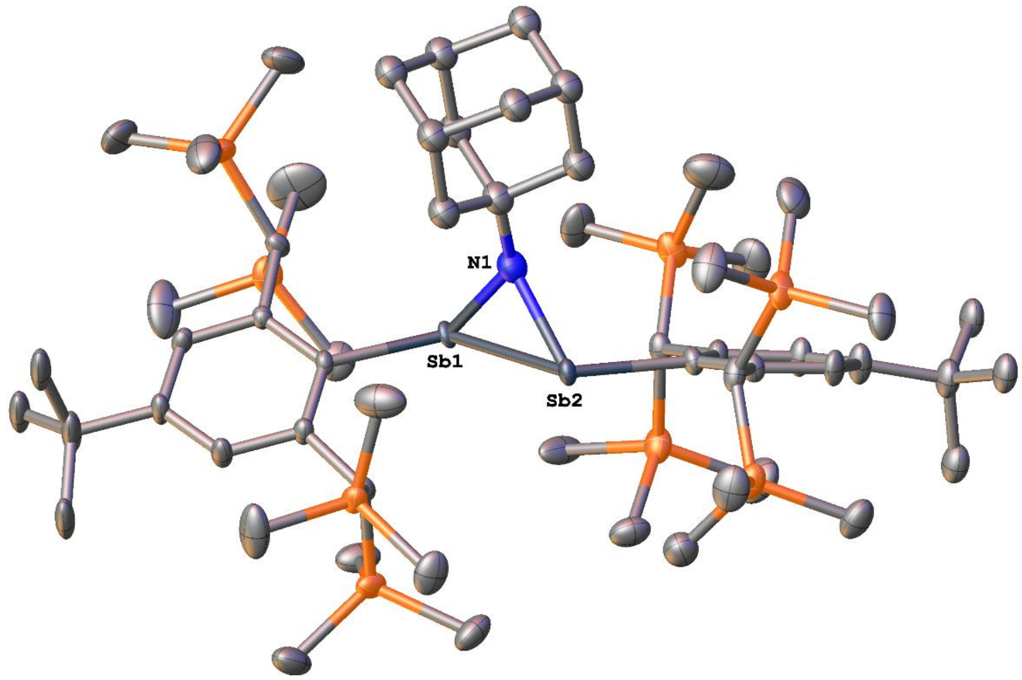

Figure S23. Solid state structure of **2d**. A side occupancy of the Sb_2NAd-moiety (occupation 18%), the solvent molecule as well as Hydrogen atoms are omitted for clarity. Thermal ellipsoids are set to 50% probability level.

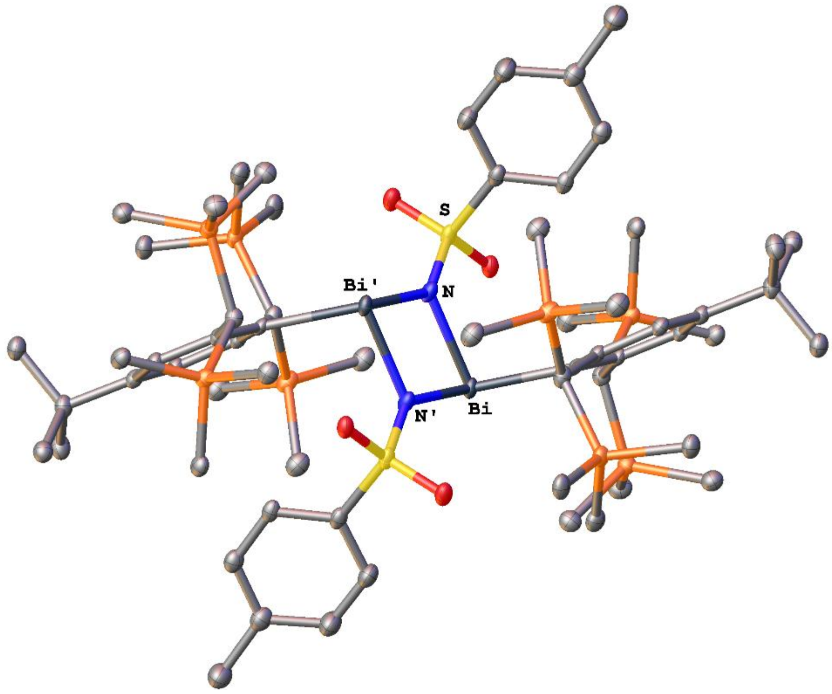

Figure S24. Solid state structure of one of the two independent molecules of **4a**. A crystallographic center of inversion in the 4-membered ring renders the two molecular parts identical. Hydrogen atoms are omitted for clarity. Thermal ellipsoids are set to 30% probability level.

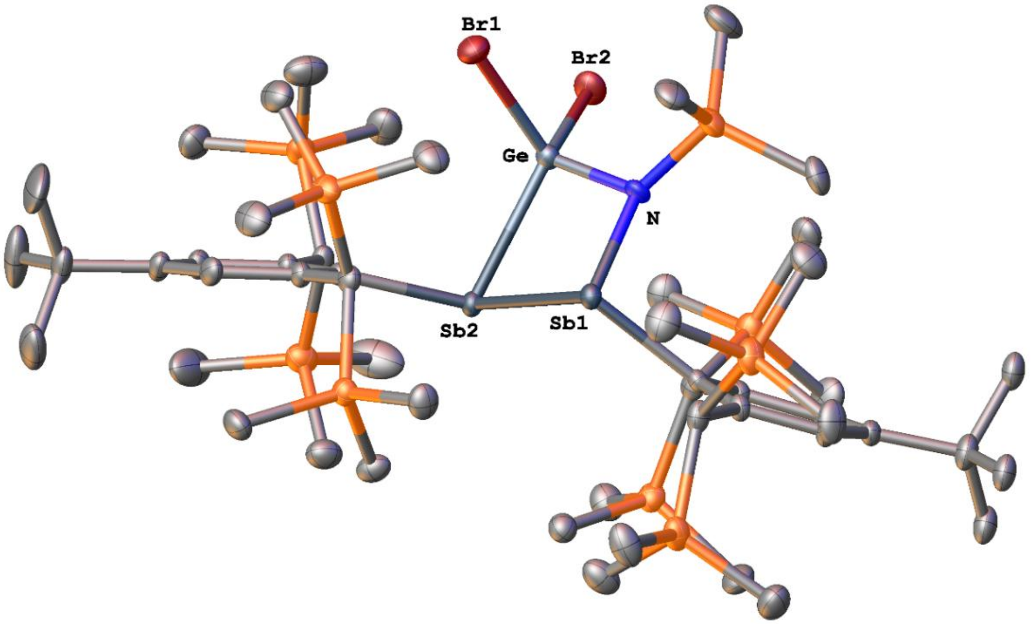

Figure S25. Solid state structure of **5**. A side occupancy of the *tert*-butyl group (33% occupancy) in one of the Tbb ligands, the co-crystallised solvent molecule as well as Hydrogen atoms are omitted for clarity. Thermal ellipsoids are set to 50% probability level.

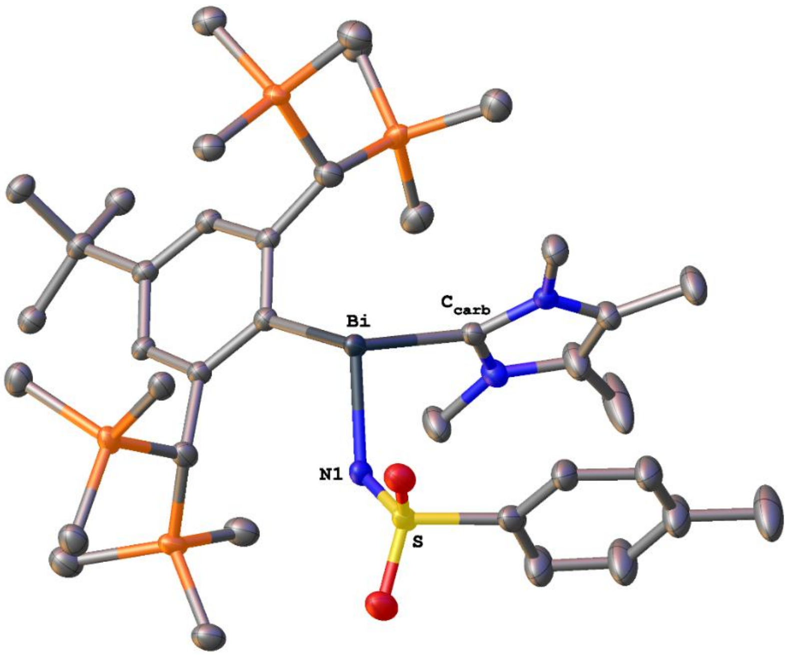

Figure S26. Solid state structure of **6**. Hydrogen atoms are omitted for clarity. Thermal ellipsoids are set to 50% probability level.

Table S6: Selected bond lengths /Å and angles /°.

entry	Sb1-Sb2	Sb1-N & Sb2-N	Sb1-C_{ipso}	Sb2-C_{ipso}	Bi-N	Bi-C_{ipso}	Bi-Bi	C_{ipso}-Sb1-Sb2-C_{ipso}
**2a**	2.8302(4)	2.120(4) & 2.118(3)	2.164(4)	2.168(4)				169.9(2)
**2b**	2.8130(3)	2.067(2) & 2.050(2)	2.1833(19)	2.189(2)				171.8(1)
**2c**	2.8254(2)	2.068(2) & 2.079(2)	2.186(2)	2.167(2)				175.1(1)
**2d**	2.8732(7)	2.069(4) & 2.070(4)	2.187(6)	2.199(5)				172.3(2)
**4a**					2.189(8) 2.212(8)	2.260(9)	3.5574(7)	
**5**	2.9274(4)	2.098(4) Sb2-Ge: 2.5989(6)	2.168(4)	2.179(4)	Ge-N: 1.810(4)			153.7(2)
**6**					2.106(11)	2.270(14)	Bi-C_{carb}: 2.344(15)	

7. NMR spectra

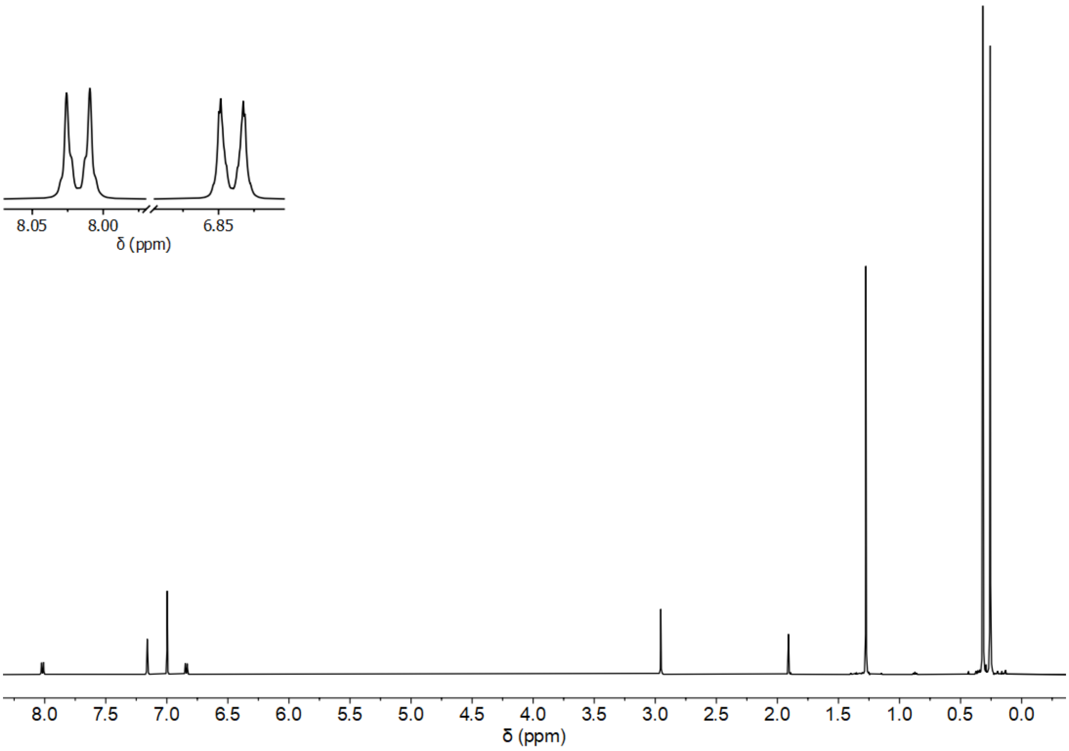

^{1}H NMR (500 MHz) spectrum of complex 2a in C_6D_6.

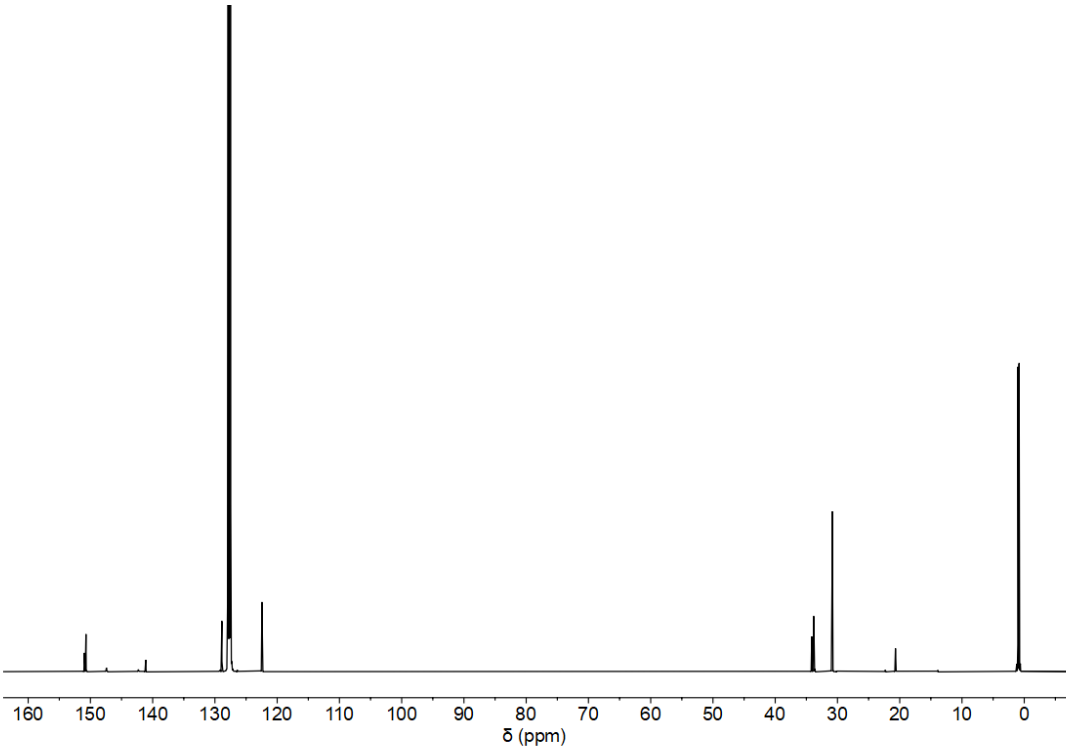

$^{13}C\{^1H\}$ NMR (126 MHz) spectrum of complex 2a in C_6D_6.

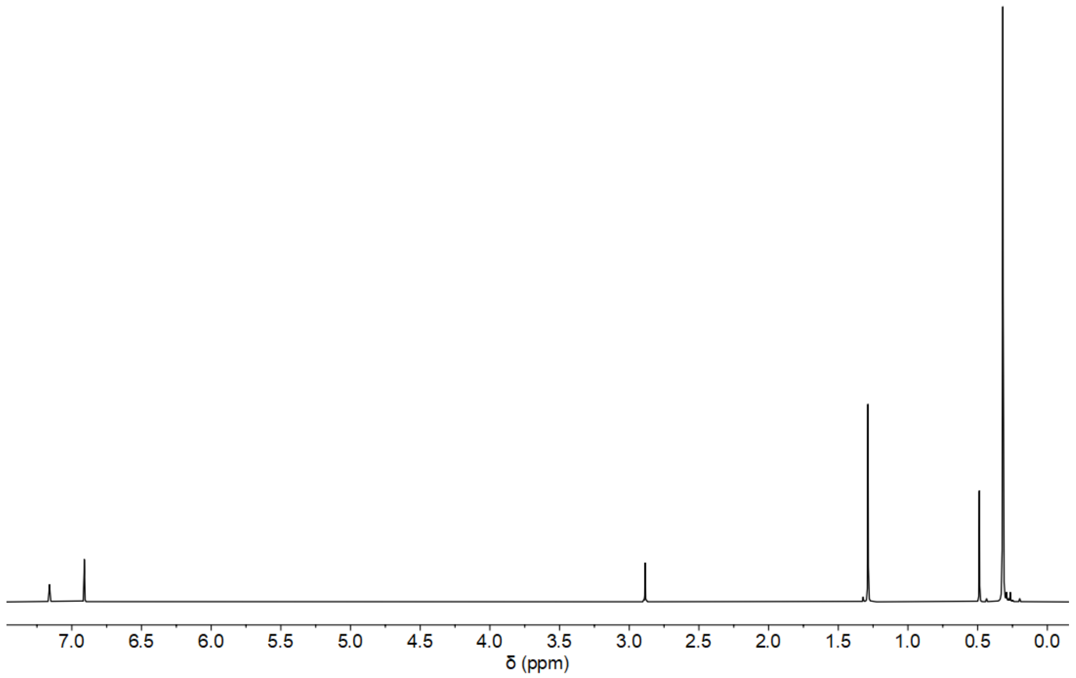

^{1}H NMR (500 MHz) spectrum of complex 2b in C_6D_6.

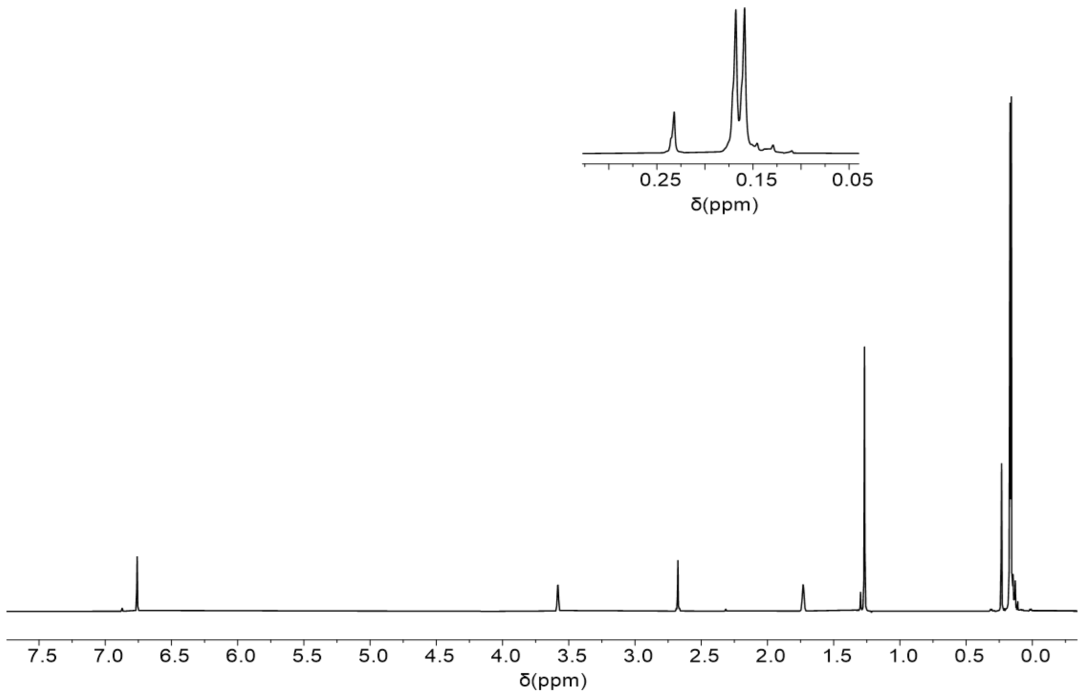

^{1}H NMR (400 MHz) spectrum of complex 2b in THF-d_8.

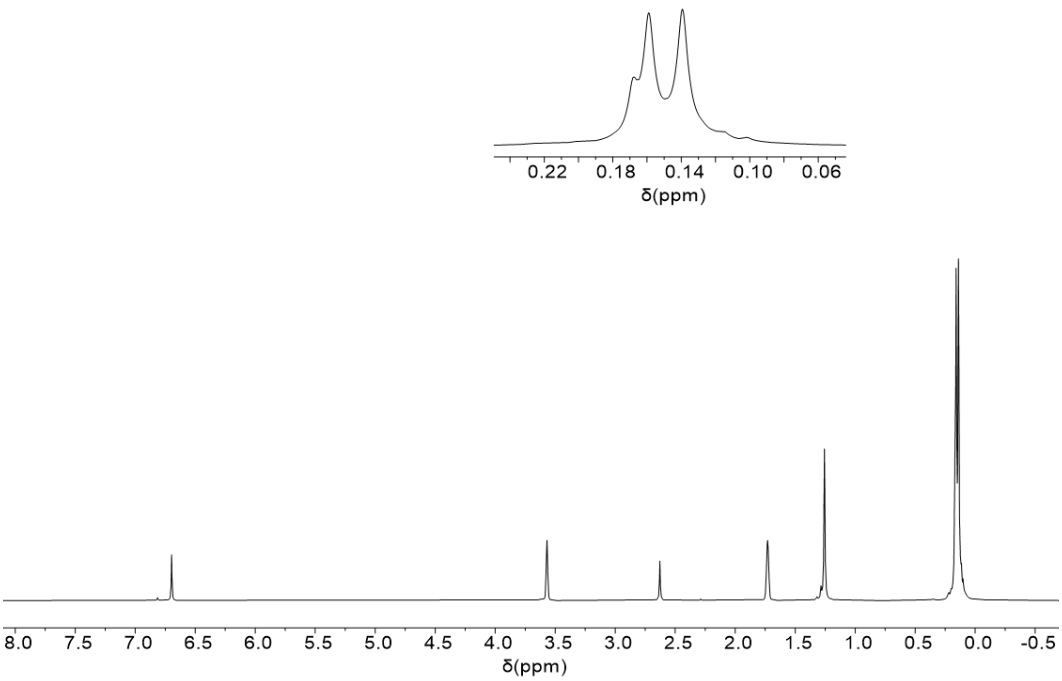

^{1}H NMR (300 MHz) spectrum of complex 2b in THF-d_8 at 193 K.

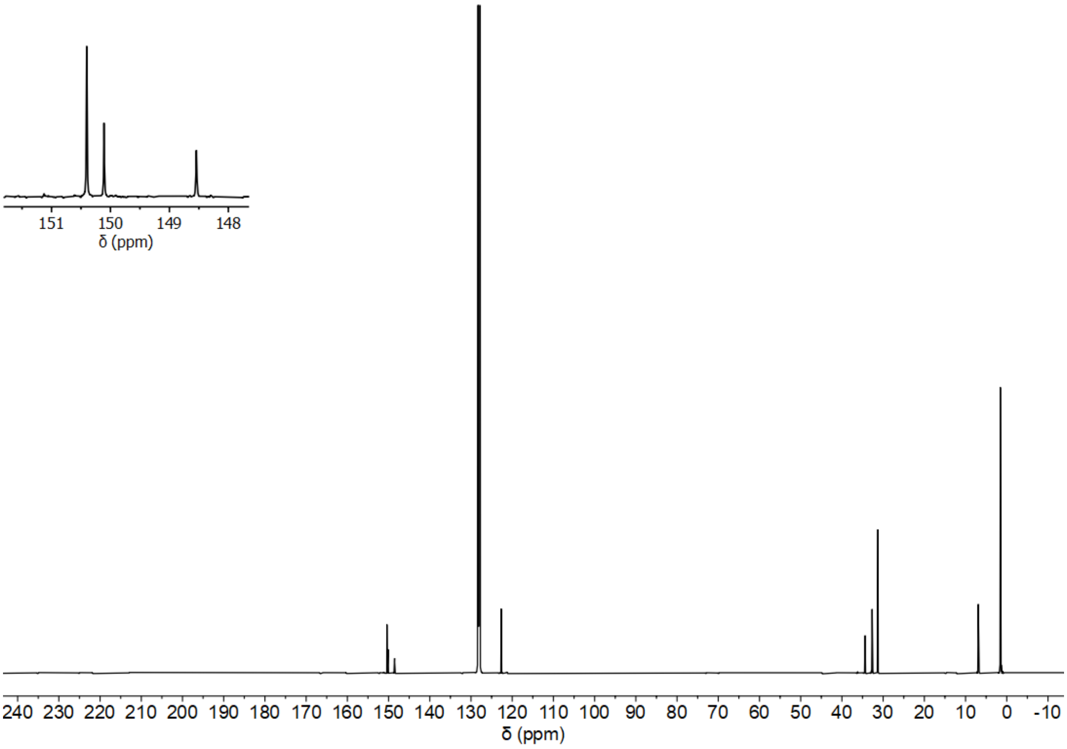

$^{13}C\{^{1}H\}$ NMR (126 MHz) spectrum of complex 2b in C_6D_6.

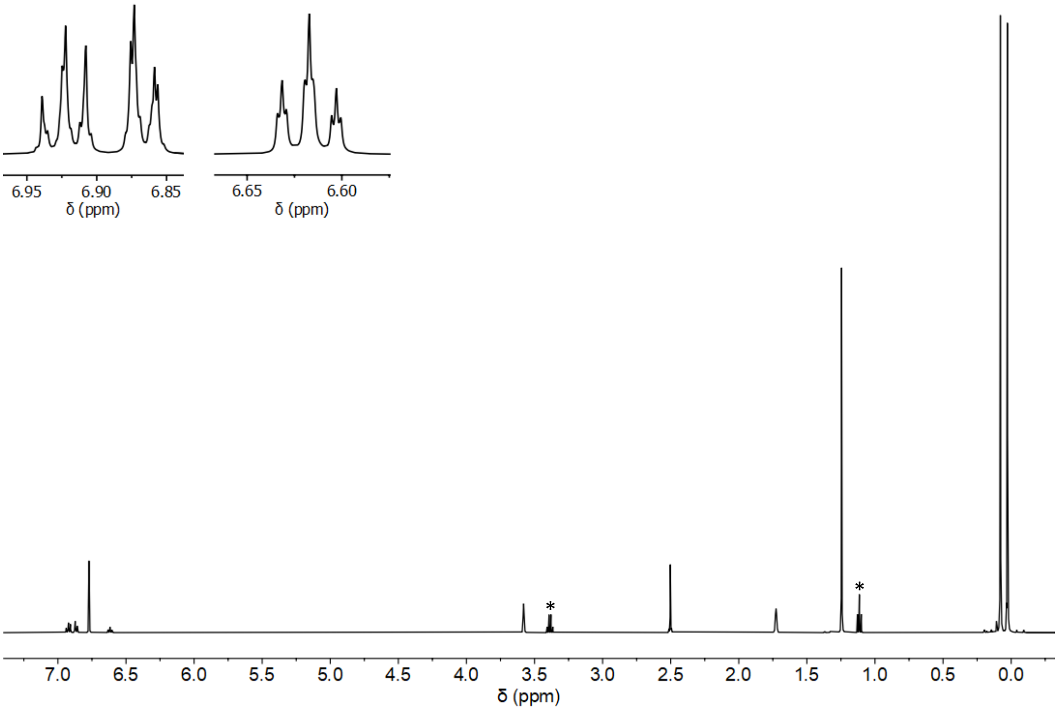

^{1}H NMR (500 MHz) spectrum of complex 2c in THF-d_8 (* = Et_2O).

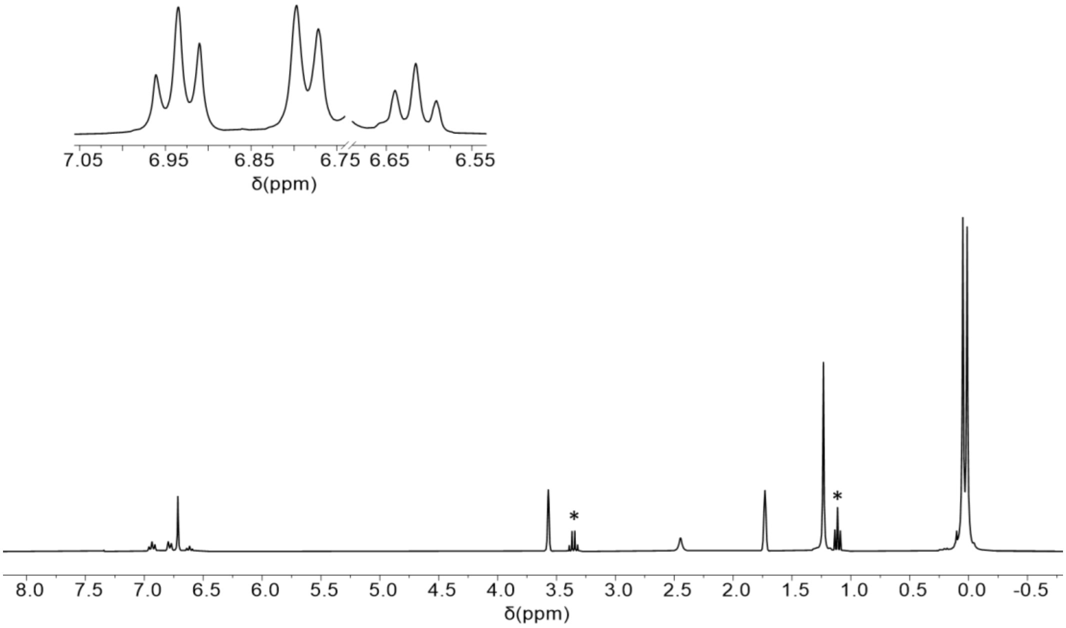

^{1}H NMR (300 MHz) spectrum of complex 2c in THF-d_8 at 193 K (* = Et_2O).

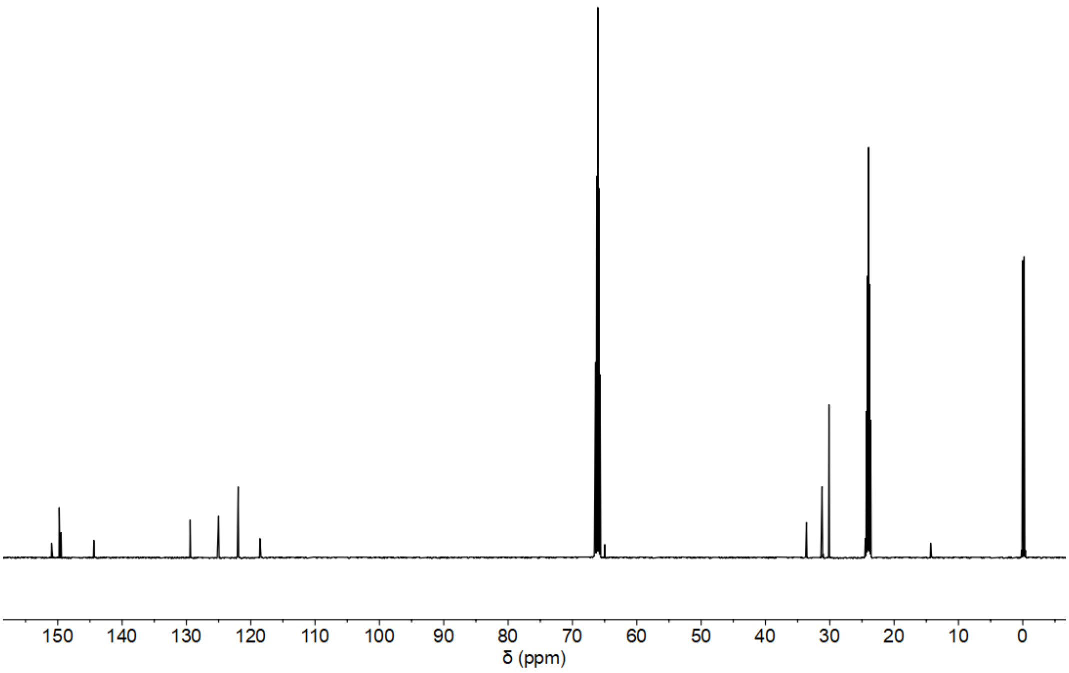

$^{13}C\{^1H\}$ NMR (126 MHz) spectrum of complex 2c in THF-d_8.

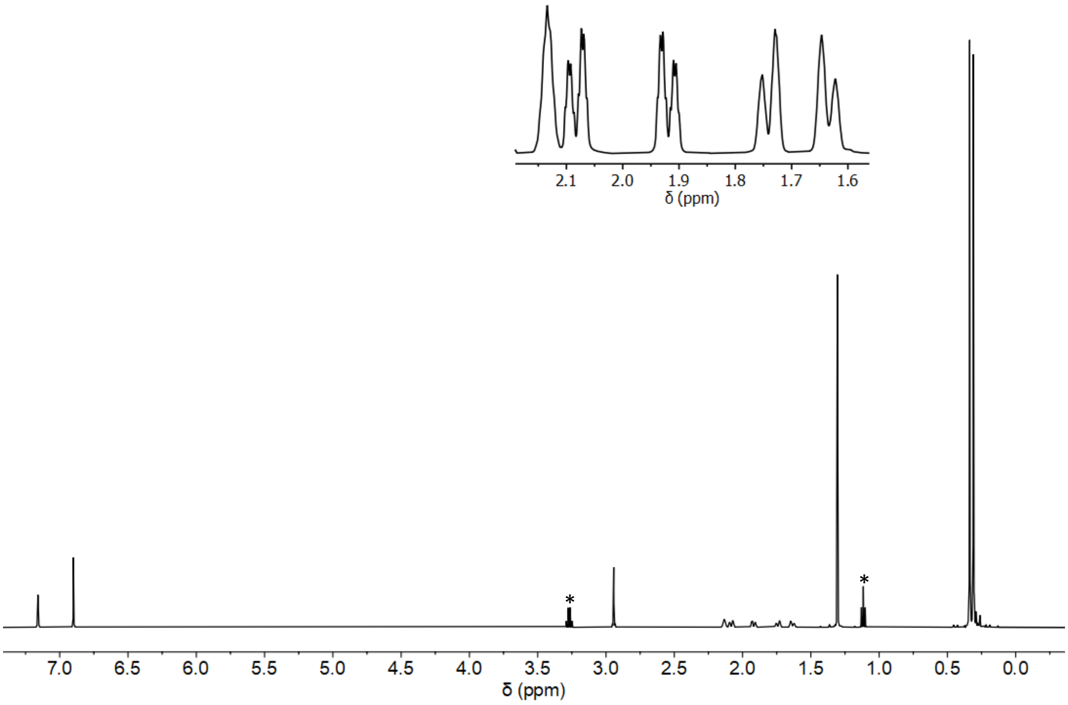

^1H NMR (500 MHz) spectrum of complex 2d in C_6D_6 (* = Et_2O).

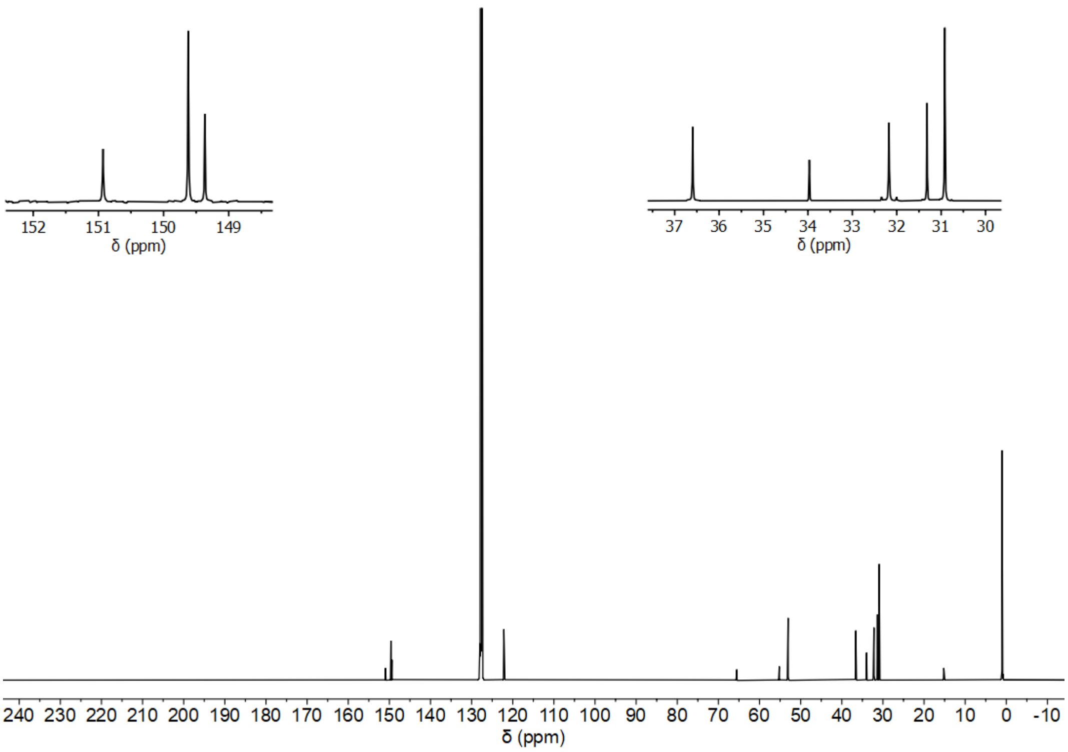

$^{13}C\{^{1}H\}$ NMR (126 MHz) spectrum of complex 2d in C_6D_6.

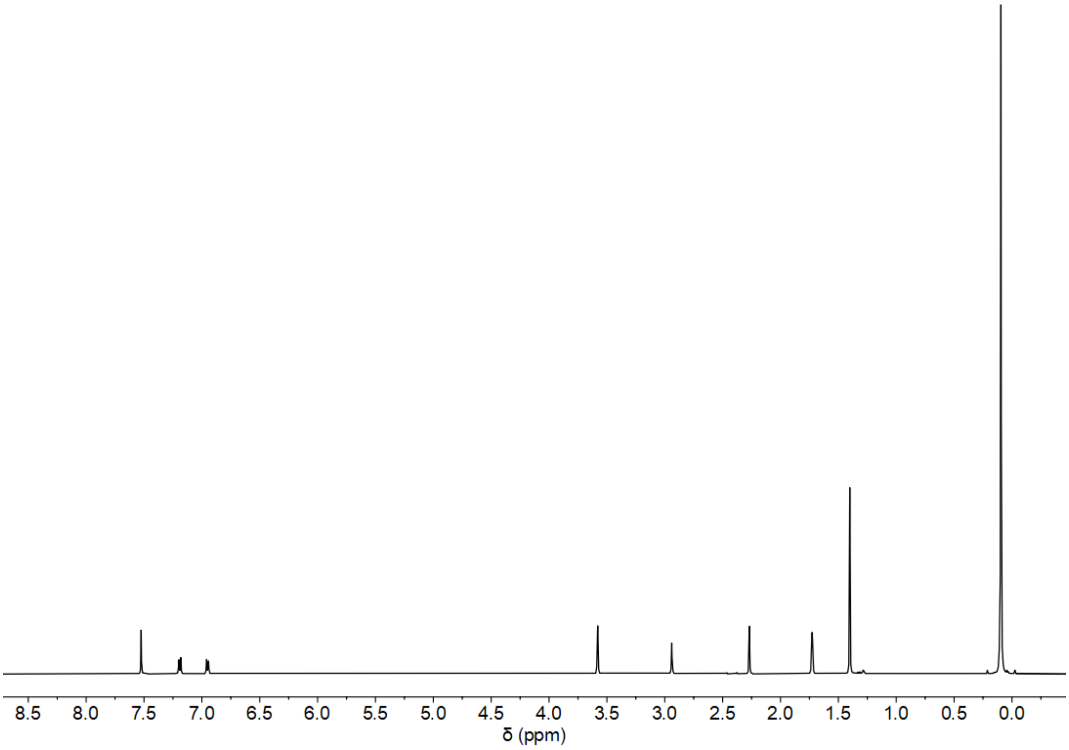

^{1}H NMR (500 MHz) spectrum of complex 4a in THF-d_8.

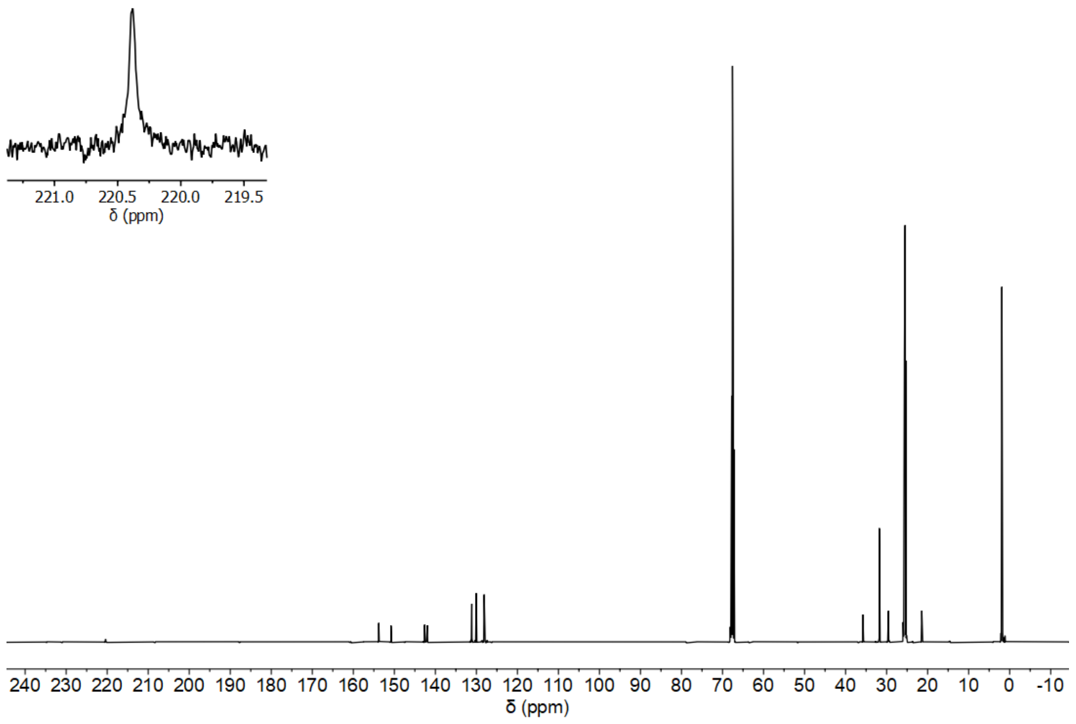

$^{13}C\{^{1}H\}$ NMR (126 MHz) spectrum of complex 4a in THF-d_8.

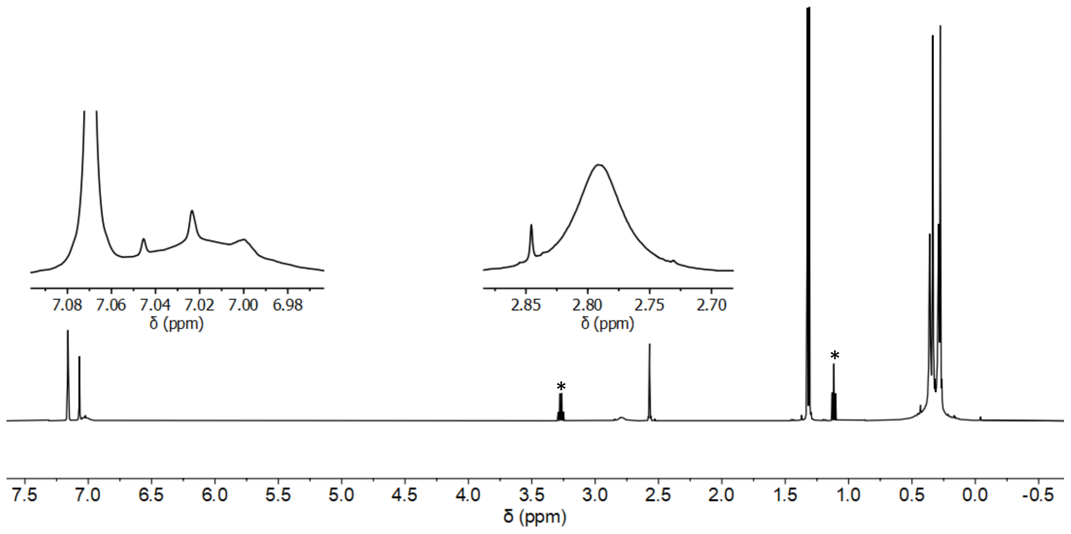

^{1}H NMR (500 MHz) spectrum of complex 5 in C_6D_6 (* = Et_2O).

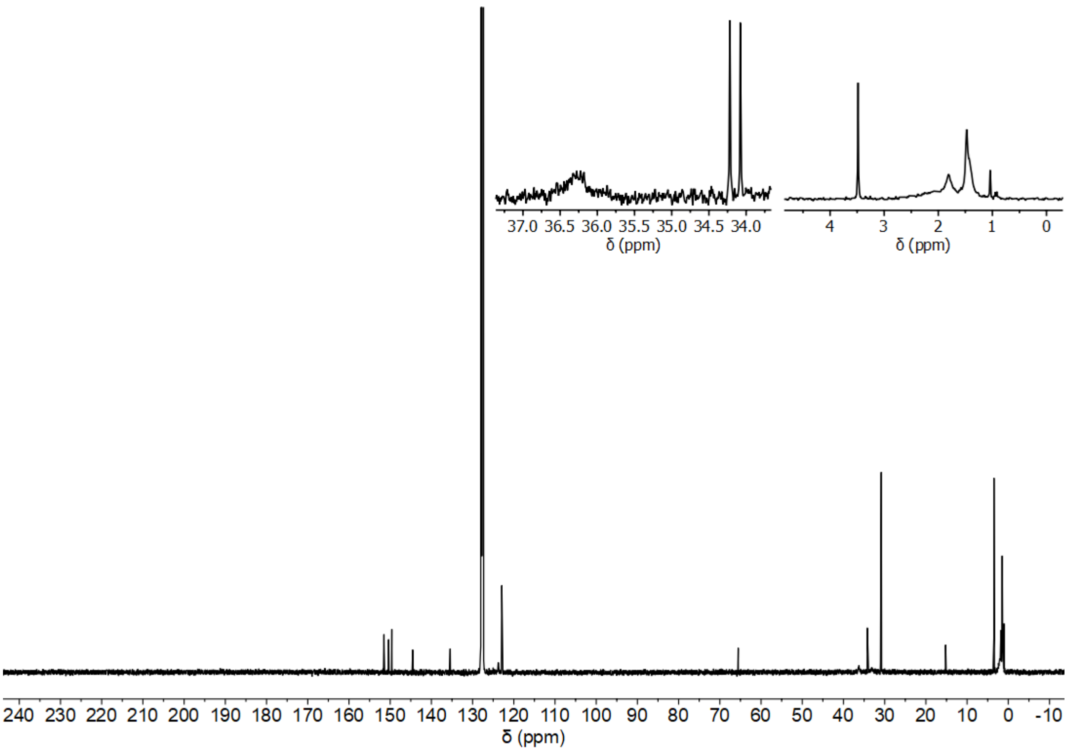

$^{13}C\{^1H\}$ NMR (126 MHz) spectrum of complex 5 in C_6D_6.

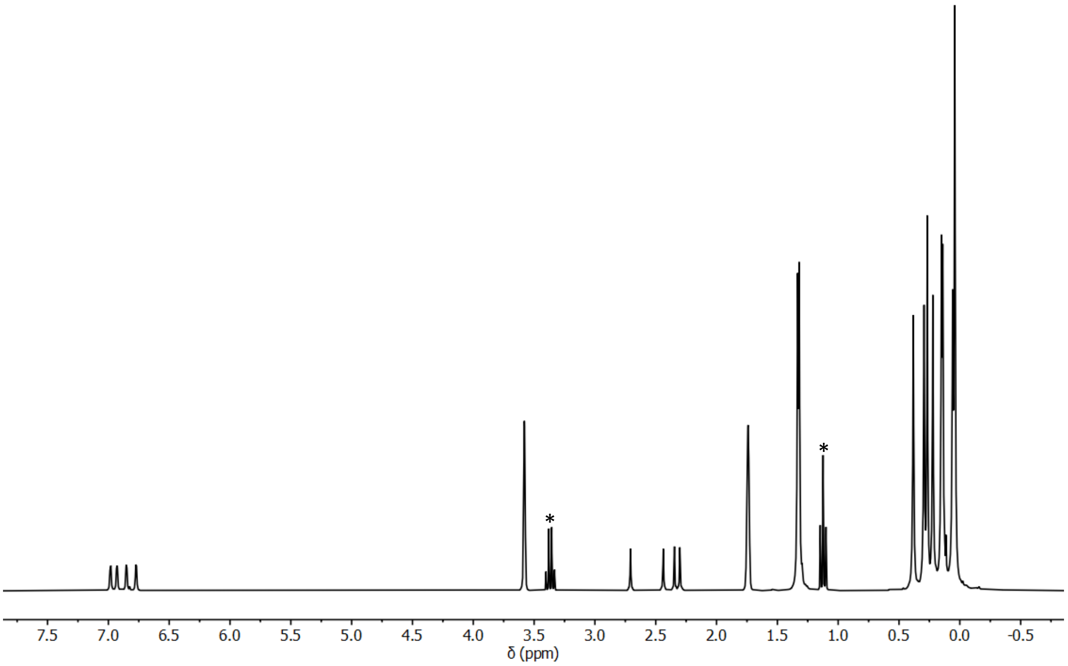

^1H NMR (300 MHz) spectrum of complex 5 in THF-d_8 at 193 K (* = Et_2O).

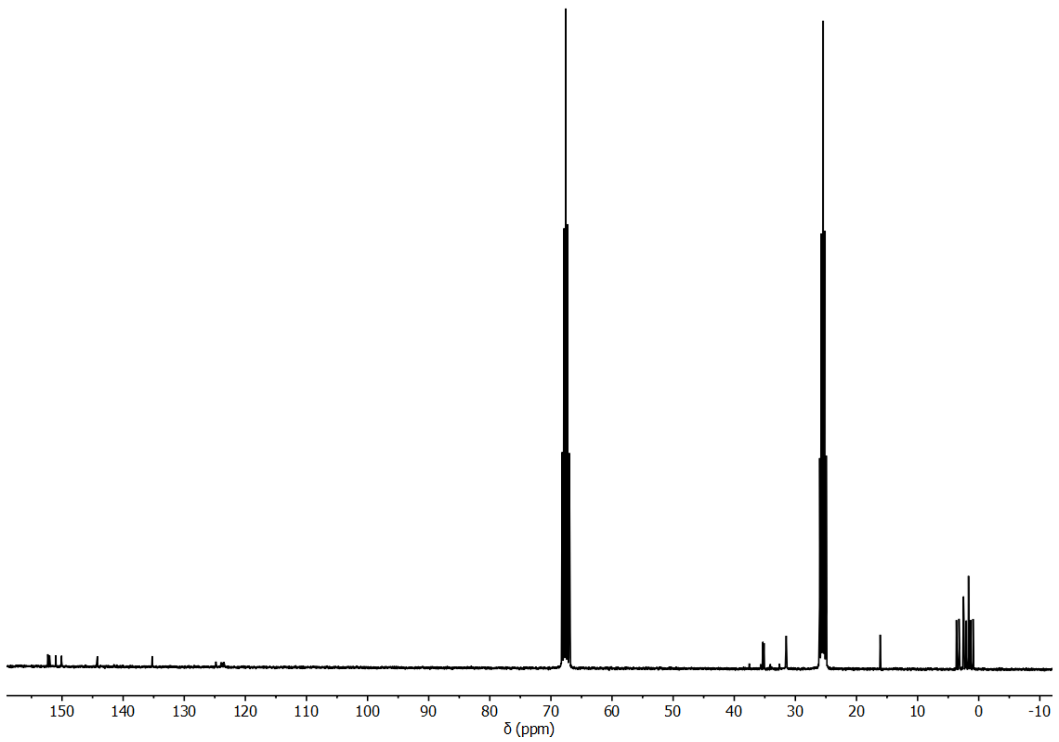

$^{13}C\{^{1}H\}$ NMR (75 MHz) spectrum of complex 5 in THF-d_8 at 193 K.

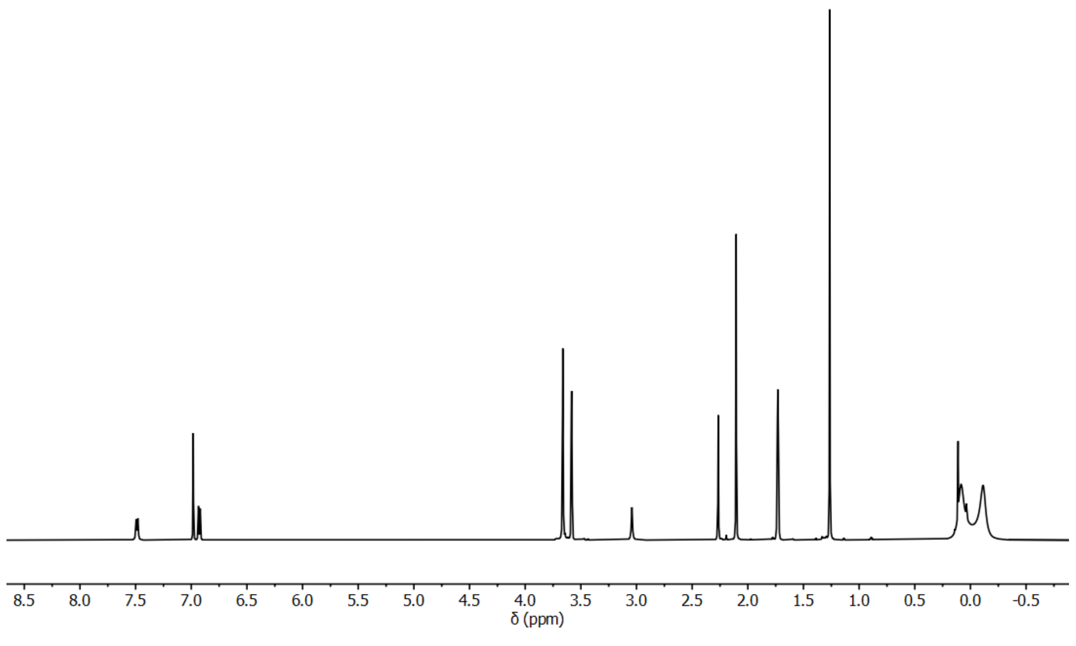

^{1}H NMR (500 MHz) spectrum of complex 6 in THF-d_8.

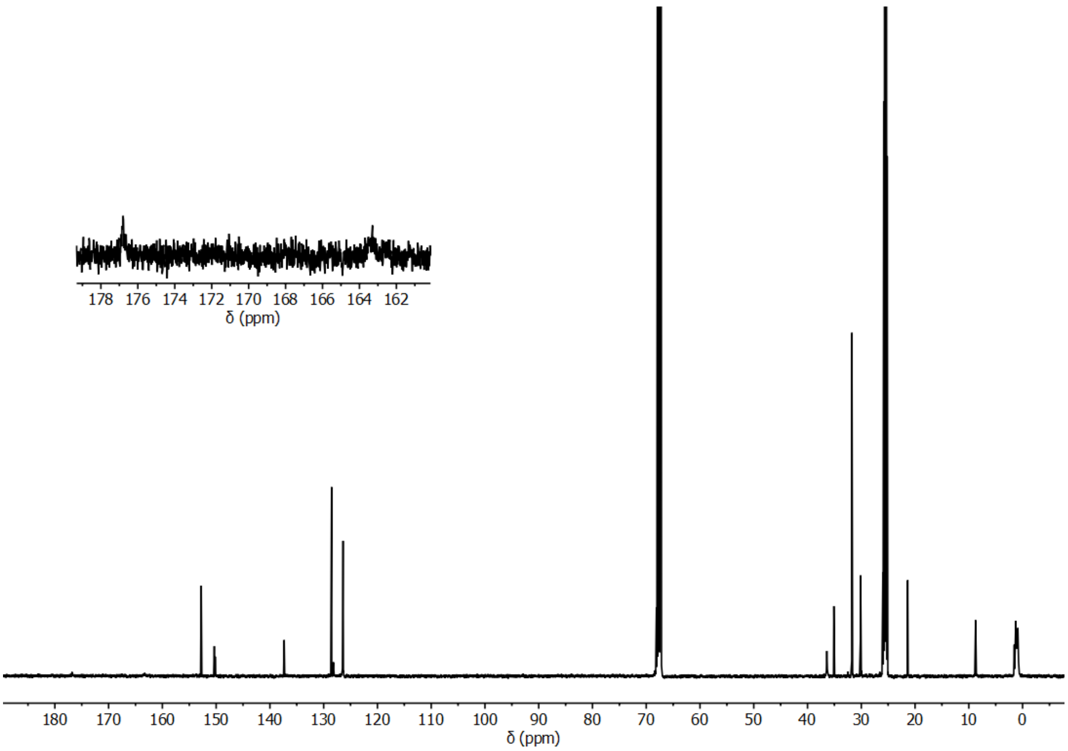

$^{13}C\{^{1}H\}$ NMR (126 MHz) spectrum of complex 6 in THF-d_8.

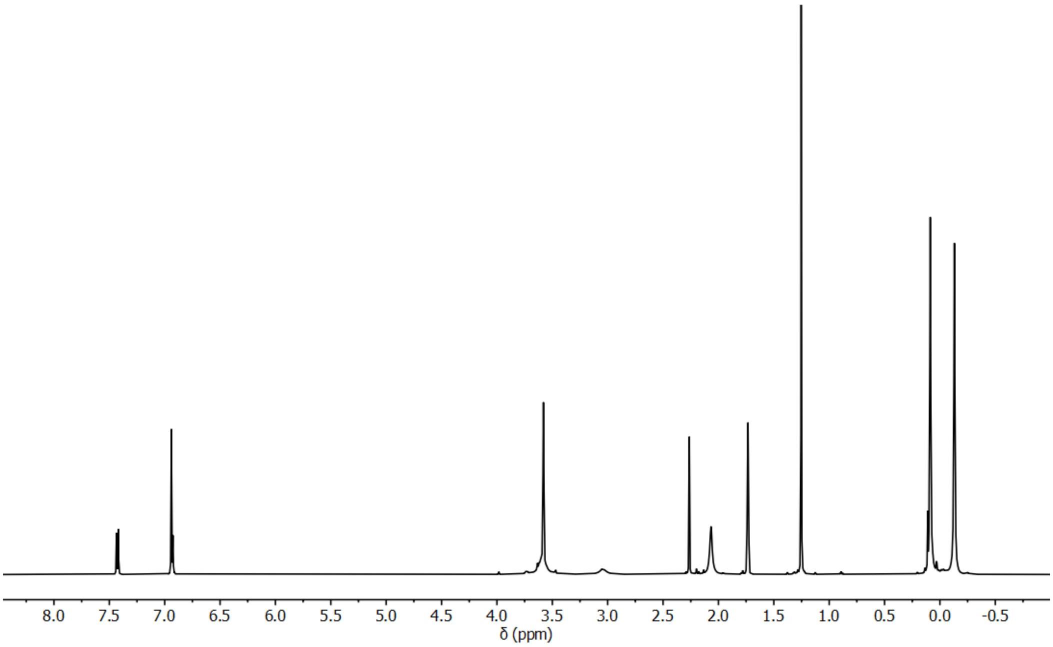

^{1}H NMR (500 MHz) spectra of complex 6 in THF-d_8 at 243 K.

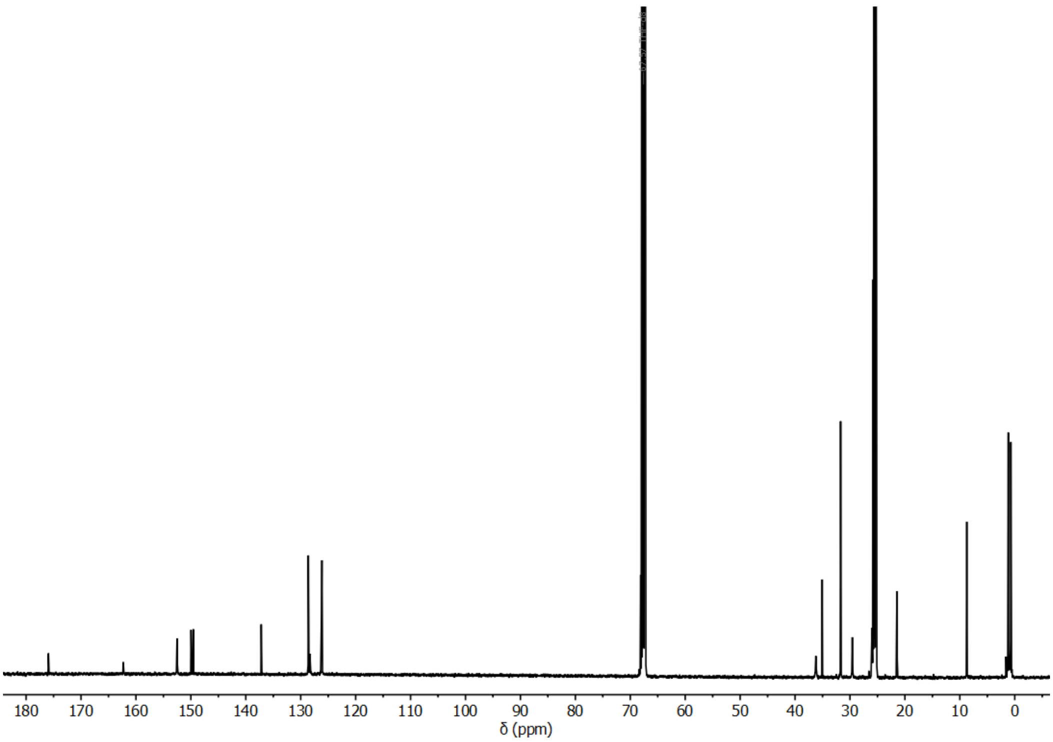

$^{13}C\{^{1}H\}$ NMR (126 MHz) spectrum of complex 6 in THF-d_8 at 243 K.

8. References

1 D. Meleschko, P. Palui, R. M. Gomila, G. Schnakenburg, A. C. Filippou, A. Frontera and A. Bismuto, *Angew. Chem. Int. Ed.*, 2024, **63**, e202405400.

2 B. Twamley, C. D. Sofield, M. M. Olmstead and P. P. Power, *J. Am. Chem. Soc.*, 1999, **121**, 3357–3367.

3 T. Sasamori, Y. Arai, N. Takeda, R. Okazaki, Y. Furukawa, M. Kimura, S. Nagase and N. Tokitoh, *Bulletin of the Chemical Society of Japan*, 2002, **75**, 661–675.

4 R. J. Wright, J. Steiner, S. Beaini and P. P. Power, *Inorganica Chimica Acta*, 2006, **359**, 1939–1946.

5 J. Kouvetakis, A. Haaland, D. J. Shorokhov, H. V. Volden, G. V. Girichev, V. I. Sokolov, and P. Matsunaga, *J. Am. Chem. Soc.*, 1998, **120**, 6738–6744.

6 N. Kuhn and T. Kratz, *Synthesis*, 1993, **1993**, 561–562.

7 F. Neese, *WIREs Comput. Mol. Sci.*, 2022, **12**, e1606.

8 M. D. Hanwell, D. E. Curtis, D. C. Lonie, T. Vandermeersch, E. Zurek and G. R. Hutchison, *J. Cheminform.*, 2012, **4**, 17.

9 J. Tao, J. P. Perdew, V. N. Staroverov and G. E. Scuseria, *Phys. Rev. Lett.*, 2003, **91**, 146401.

10 E. Caldeweyher, C. Bannwarth and S. Grimme, *The Journal of Chemical Physics*, 2017, **147**, 034112.

11 E. Caldeweyher, S. Ehlert, A. Hansen, H. Neugebauer, S. Spicher, C. Bannwarth and S. Grimme, *The Journal of Chemical Physics*, 2019, **150**, 154122.

12 E. Caldeweyher, J.-M. Mewes, S. Ehlert and S. Grimme, *Phys. Chem. Chem. Phys.*, 2020, **22**, 8499–8512.

13 L. Wittmann, I. Gordiy, M. Friede, B. Helmich-Paris, S. Grimme, A. Hansen and M. Bursch, *Phys. Chem. Chem. Phys.*, 2024, **26**, 21379–21394.

14 F. Weigend and R. Ahlrichs, *Phys. Chem. Chem. Phys.*, 2005, **7**, 3297.

15 F. Neese, *J. Comput. Chem.*, 2003, **24**, 1740–1747.

16 G. L. Stoychev, A. A. Auer and F. Neese, *J. Chem. Theory Comput.*, 2017, **13**, 554–562.

17 L. Goerigk and S. Grimme, *J. Chem. Theory Comput.*, 2011, **7**, 291–309.

18 D. Peng, N. Middendorf, F. Weigend and M. Reiher, *The Journal of Chemical Physics*, 2013, **138**, 184105.

19 Y. J. Franzke, R. Treß, T. M. Pazdera and F. Weigend, *Phys. Chem. Chem. Phys.*, 2019, **21**, 16658–16664.

20 S. Kossmann and F. Neese, *J. Chem. Theory Comput.*, 2010, **6**, 2325–2338.

21 G. Knizia, *J. Chem. Theory Comput.*, 2013, **9**, 4834–4843.

22 J. Pipek and P. G. Mezey, *The Journal of Chemical Physics*, 1989, **90**, 4916–4926.

23 P. Palui, S. Ghosh, R. M. Gomila, G. Schnakenburg, A. Frontera and A. Bismuto, *J. Am. Chem. Soc.*, 2025, **147**, 1421–1426.

24 R. F. W. Bader, *Chem. Rev.*, 1991, **91**, 893–928.

25 T. Lu and F. Chen, *J. Comput. Chem.*, 2012, **33**, 580–592.

26 T. Lu, *The Journal of Chemical Physics*, 2024, **161**, 082503.

27 E. D. Glendening, C. R. Landis and F. Weinhold, *J. Comput. Chem.*, 2019, **40**, 2234–2241.

28 T. Ziegler and A. Rauk, *Theoret. Chim. Acta*, 1977, **46**, 1–10.

29 A. Michalak, M. Mitoraj and T. Ziegler, *J. Phys. Chem. A*, 2008, **112**, 1933–1939.

30 M. P. Mitoraj, A. Michalak and T. Ziegler, *J. Chem. Theory Comput.*, 2009, **5**, 962–975.

31 I. Mayer, *J. Comput. Chem.*, 2007, **28**, 204–221.

32 M. Cossi, N. Rega, G. Scalmani and V. Barone, *J. Comput. Chem.*, 2003, **24**, 669–681.

33 E. F. Pettersen, T. D. Goddard, C. C. Huang, G. S. Couch, D. M. Greenblatt, E. C. Meng and T. E. Ferrin, *J. Comput. Chem.*, 2004, **25**, 1605–1612.

34 M. Steinmetz and S. Grimme, *ChemistryOpen*, 2013, **2**, 115–124.

35 J. Koziskova, F. Hahn, J. Richter and J. Kožíšek, *Acta Chimica Slovaca*, 2016, **9**, 136–140.

36 G. M. Sheldrick, *Acta Crystallogr. A Found Crystallogr.*, 2008, **64**, 112–122.

37 SADABS-2016/**2**, Bruker AXS 2016.

38 O. V. Dolomanov, L. J. Bourhis, R. J. Gildea, J. A. K. Howard and H. Puschmann, *J. Appl. Crystallogr.*, 2009, **42**, 339–341.

39 D. Kratzert, *Final Cif*, vol. V113.
